# Diffusion tensor imaging in chronic tension-type headache

**DOI:** 10.3389/fpain.2026.1850836

**Published:** 2026-07-01

**Authors:** M. Teepker, L. Vacik, A. M. Hermsen, K. Menzler, V. Mylius, L. Timmermann, S. Knake, M. Belke

**Affiliations:** 1 Department of Neurology, Philipps-University of Marburg, Marburg, Germany; 2 Hardtwaldklinik 1, Department of Neurology, Bad Zwesten, Germany; 3 Diakonie Kork, Epilepsy Center, Kehl-Kork, Germany; 4 LOEWE Research Cluster for Advanced Medical Physics in Imaging and Therapy (ADMIT), TH-Mittelhessen University of Applied Sciences, Giessen, Germany; 5 Department of Neurology, Center for Neurorehabilitation, Valens, Switzerland

**Keywords:** AD, chronic tension-type headache, DTI, headache, microstructural changes, MRI, NODDI, RD

## Abstract

**Background:**

Tension-type headache (TTH) is the most common primary headache disorder and chronic tension-type headache (CTTH) is difficult to treat. Despite this clinical importance, the pathophysiology especially of CTTH remains unclear. Therefore, the aim of this study was to search for microstructural changes in CTTH to further evaluate the pathogenesis of CTTH.

**Methods:**

Nine female patients suffering from CTTH and ten healthy controls matched by age and gender were included. All participants underwent a magnetic resonance imaging (MRI) scan with a diffusion tensor imaging (DTI) sequence. From the diffusion tensor the Fractional Anisotropy (FA), the Axial Diffusivity (AD), the Radial Diffusivity (RD), the NODDI parameters, the Orientation Dispersion Index (ODI), and the Neurite Density Index (NDI) were calculated.

**Results:**

Microstructural changes were found in the right cerebellum, tectum, right occipital fusiform gyrus, left inferior temporal gyrus, left cingulate gyrus (anterior and posterior devision), right thalamic radiation, right lateral occipital cortex (inferior division), left cerebellum, left insular cortex, left precuneous, left central opercular cortex, left inferior frontal gyrus, left middle temporal gyrus, right middle temporal gyrus, left middle frontal gyrus, right postcentral gyrus, left superior longitudinal fasciculus, and the right paracingulate gyrus.

**Conclusions:**

Patients suffering from CTTH had microstructural changes in multiple brain areas. Those were related to brain regions that belong to or interact with the pain matrix, trigeminal system or association fibers.

## 1 Introduction

Tension-type headache (TTH) is the most prevalent primary headache disorder, with a global prevalence of 26%–38% (1, 2). It is characterized by mild to moderate, bilateral, non-pulsating pain without vegetative or autonomic features, often described as a tight band around the head (3–5). Psychiatric diseases [especially anxiety (6), depression (6) and sleep disturbances (7)] as well pain disorders [e.g., migraine (8), neck (9) or low back pain (10)] are more prevalent in patients suffering from TTH.

TTH is classified as episodic (<15 days/month, ETTH) or chronic (≥15 days/month) (4), with chronic TTH (CTTH) affecting 0.5%–4.8% of the population and posing a significant treatment challenge (11). The prevalence of CTTH is up to 3.0%–3.9% higher in women than in men (12, 13).

In primary headache disorders including TTH, the trigeminovascular system provides the neuroanatomical basis for pain and nociception as trigeminal fibers of the ophthalmic branch innervate meningeal structures and blood vessels known to be the origin of pain. Calcitonin gene-related peptide (CGRP) is a key transmitter in the trigeminal system (14, 15).

Tenderness of the pericranial muscles is evident in some patients indicating a local soft tissue injury or inflammation. By this, a continuous nociceptive input might be generated that is able to induce and maintain central sensitization of second order neurons (at the level of the spinal trigeminal nucleus and dorsal horn) as well as of supraspinal structures (e.g., somatosensory cortex, thalamus or limbic system [for review see (2, 5, 115)]). Whereas peripheral factors seem to play a key role in the pathophysiology of ETTH, central mechanisms such as central sensitization might be responsible for the generation of CTTH. Further central pathophysiological alterations include an impairment of endogenous pain inhibitory systems as demonstrated by Pielsticker et al. in an experimental DNIC (diffuse noxious inhibitory controls)-paradigm in CTTH (116). In addition to this, Schmidt-Wilcke et al. described a gray matter decrease in patients with CTTH using MRI and voxel-based morphometry (VBM). These changes referred to cerebral regions that contribute to the so-called “pain matrix”, e.g., the anterior cingulate cortex (ACC), the insula and parahippocampus bilaterally, as well as the right cerebellum (16). CGRP, an important trigeminal neurotransmitter, plays a crucial role in migraine and rises in the blood during migraine attacks (117, 118) but remains normal in TTH (17). However, Ashina et al. note that due to methodological limitations a role of CGRP in tension-type headache cannot be completely ruled out (5, 18).

In contrast to VBM—an MRI technique to investigate focal differences of gray matter—diffusion tensor imaging (DTI) indicates microstructural changes which have been reported for various psychiatric (19) and neurological diseases (20–22) as well as headache disorders. Our group found microstructural changes in patients suffering from episodic cluster headache in regions that belong to the pain matrix (23). In their review, Rahimi et al. concluded that microstructural abnormalities occur in migraine and are probably related to neuronal damage and plasticity (24). Patients with new daily persistent headache show widely distributed abnormalities in the white (25) and gray matter (26).

In spite of the pathophysiological findings described above and the prevalence of TTH, its pathophysiology—especially of CTTH—remains unclear. Since central mechanisms seem to cause CTTH and gray matter changes are seen in the pain matrix of affected patients, it is possible that microstructural alterations are evident in both white and gray matter and contribute to chronic pain in CTTH and their comorbid diseases. This tempted us to use DTI in order to further evaluate the microstructural integrity in CTTH conducting an exploratory whole brain analysis. We derived several scalar indices from the diffusion tensor to examine gray and white matter across the whole brain. Fractional anisotropy (FA) was calculated as an indicator of microstructural organization and potential lesions (27). To further characterize tissue properties, we also computed axial diffusivity (AD), reflecting diffusion parallel to the main fiber orientation and linked to axonal integrity (28), and radial diffusivity (RD), representing diffusion perpendicular to the main fiber orientation and associated with myelination or glial morphology. In addition, we estimated the neurite density index (NDI) and orientation dispersion index (ODI) from the NODDI model (29), which provide voxel-wise measures of neurite density and the variability of neurite orientations. Notably, NODDI metrics have recently been validated histologically (30) and were reported in new daily persistent headache as a chronic headache disorder, too (26).

## 2 Methods

### 2.1 Subjects

Participants were recruited via wall posters, advertisements in local newspapers and from the outpatient clinic of the Neurological Department, University of Marburg. They were paid for attendance and gave written informed consent before participating in the study. The experimental protocol was approved by the ethics committee of the medical school of the University of Marburg.

Before inclusion, patients were examined by a headache-specialized neurologist and had to document their headaches in a diary over at least four weeks. They had to note the onset and the end of their headaches and to rate the severity of their headache on a numeric rating scale (NRS) from zero (no pain) to ten (maximal pain). By this, the duration of pain in hours and the total sum of pain intensity per hour in one month could be calculated (Table 1).

**Table 1:** Demographics of the CTTH and control group.

Measure	CTTH			Control		
	Mean	Range	Standard deviation	Mean	Range	Standard deviation
Age	37.9	18–63	18.6	37.3	24–62	14.3
BMI	23.7	19.7–31.1	3.8	22.4	19.3–38.4	5.7
Disease duration		1 - > 50				
Days with headache per month	22.9	15–31	6.3			
Hours with headaches per month	253.7	85–394	109.8			
Sum of NRS per hour in 1month	992.8	241–2,190	635.4			

No statistically significant differences were found between the CTTH and control groups for age (Mann–Whitney *U*-test *p* = 0.94) or BMI (Mann–Whitney *U*-test *p* = 0.31).

BMI, body mass index; NRS, numeric rating scale.

Participants had to suffer from CTTH according to the guidelines of the International Headache Society (4) without any abuse of analgetics (i.e., intake of analgetics on less than 10 days per month). A medical headache prophylaxis was allowed if no clinical effects were reported. Furthermore, participants were screened for depression using “Becksches Depressionsinventar II (Beck-Depression-Inventory, BDI-II)” (31).

Exclusion criteria for all participants were: Clinical signs of a relevant depression during the interviews or a BDI-II score of more than 14 points, any other psychiatric or neurological diseases with corresponding medications, organic or traumatic brain lesions or dysfunctions, chronic pain in other localizations and contraindications for an MRI examination (e.g., ferromagnetic implants or claustrophobia).

### 2.2 MRI acquisition

Patients and controls were scanned on a 3 T Trio scanner (Siemens, Erlangen, Germany) at the Center of Brain Imaging in Marburg, Germany. The acquisition protocol comprised the following sequences:

1. 3D T1-weighted Magnetization Prepared Rapid Gradient Echo (MPRAGE) (Field of view (FOV) = 176 × 256 × 256 voxel, 1 mm^3^ isovoxel, repetition-time (TR) = 1.9 s, echo-time (TE) = 0.00252 s, inversion time (TI) = 0.9 s, flip-angle=9°, bandwidth (BW) = 170 Hz/Pixel).
2. Diffusion weighted scan [FOV = 128 × 128 × 64 voxel, 2 × 2 × 2.4 mm^3^ voxel, TR = 10.7 s, TE = 0.104 s, flip-angle=90°, BW = 1,447 Hz/Pixel, 1 image with b = 0 s/mm^2^ (b0) and 30 images with b = 1,000 s/mm^2^].

All images were investigated to be free of motion or ghosting and high frequency and/or wrap-around artifacts at the time of image acquisition.

### 2.3 MRI processing

The MPRAGE scans were transformed into a conform space with 1 mm isovoxel and a FOV of 256 × 256 × 256 Voxel. The MPRAGE scans were analyzed using the Freesurfer image analysis suite version 7.4.1, which is documented and freely available for download online (https://surfer.nmr.mgh.harvard.edu/) (32), as reported previously (33).

Briefly, the processing includes removal of non-brain tissue using a hybrid watershed/surface deformation procedure (34), automated Talairach transformation, segmentation of the subcortical white matter and deep gray matter volumetric structures (35, 36), intensity normalization (37), tessellation of the gray matter/white matter boundary, automated topology correction (38, 39) and surface deformation following intensity gradients (38, 40)⁠. All steps listed above are implemented in the automated processing stream realized with the recon-all script which was used with standard parameters.

Freesurfer morphometric procedures have been demonstrated to show good test-retest reliability across scanner manufacturers and across field strengths (41, 42). The DTI scans were preprocessed using FSL 6.0.7.3 (https://www.fmrib.ox.ac.uk/fsl) (43). For motion and residual eddy current correction each directional volume from the diffusion dataset was registered and resampled to the b0 volume (44)⁠, using the FSL script eddy_correct. The registration matrices were checked for sanity, and a signal-to-noise ratio was calculated for every subject using a python script. After checking, the diffusion tensor was calculated for each voxel using a linear regression fit to the diffusion signal.

The first b0 image of each scan was linearly registered to the anatomical T1 space using a boundary based method (45)⁠. Then brain masks were created from the FreeSurfer segmentation using all cortical, subcortical and white-matter segments. These brain masks were transformed into the diffusion space, using the inverse of the coregistration matrix.

For the evaluation of microstructural changes, the fractional anisotropy (FA) was derived from the diffusion tensor as previously described (27, 46)⁠. Additionally, the axial diffusivity (AD) (*λ*_1_) and the radial diffusivity (RD) (*λ*_2_+λ_3_]/2) (47) were calculated from the 3 eigenvalues (*λ*_1_, *λ*_2_, *λ*_3_) of the diffusion tensor (48–50)⁠. Furthermore, NODDI-DTI (51), a modification of NODDI (29), was used to obtain neurite density index (NDI) and orientation dispersion index (ODI). This was realized using a python script using the formulas from Edwards et al. (51), based on the DTI-NODDI script available at https://github.com/dicemt/DTI-NODDI.

To calculate a voxelwise statistical analysis the FA maps were first linearly and afterwards nonlinearly registered to the MNI 152 standard space (44, 52, 53).

The resulting warpfields were used to transform the FA-, AD-, RD- NDI- and ODI-maps, as well as the brain masks to the standard space, which were used to mask the transformed maps.

Voxelwise cross-subject statistics were carried out as previously described (54)⁠. Briefly the transformed and masked maps of all subjects were concatenated in one 4D nifti file which was used as input for the mri_glmfit tool of Freesurfer, which fits the data into a generalized linear model (GLM). The patients' age and body mass index (BMI) were included as a confounding variable in the GLM.

The results were corrected for multiple comparisons using a permutation-based approach (55)⁠, implemented in the Freesurfer Tool mri_glmfit-sim. Therefore, 12.000 simulations were performed under the null hypothesis, including all confounding variables; this approach is based on the Analysis of Functional NeuroImages (AFNI) null-z simulator (AlphaSim, http://afni.nimh.nih.gov/afni/doc/manual/AlphaSim.pdf). Voxels with a significance of *p* < 0.01 were clustered for each permutation and the maximum cluster size was obtained. Using the results of the permutations a clusterwise *p*-value (CWP) was calculated for each cluster. Clusters with a CWP < 0.05 after correction for multiple testing by the permutation-based approach were accepted as significant.

## 3 Results

Totally, 69 (54 women and 15 men) individuals were screened for inclusion in our study. After screening procedure, 9 women suffering from CTTH—and no men—fulfilled our strict inclusion criteria and could be included (mean age 37.9 ± 18.6 years). The duration of the CTTH disorder ranged from one year to over 50 years. Three subjects were treated prophylactically with amitriptyline, topiramate or gabapentin, respectively, however, they all described no clinical improvement when included in this study. Further detailed characteristics such as accompanying diseases are demonstrated in Table 2. Ten healthy female controls without any medication (mean age 37.3 ± 14.3 years) were matched for age. Demographic details of both groups can be found in Table 1. No statistically significant differences were found between the CTTH and control groups for age (Mann–Whitney *U*-test: *p* = 0.94) or BMI (Mann–Whitney *U*-test: *p* = 0.31). All subjects had a full secondary education. Further tertiary education was not tracked.

**Table 2:** Basic clinical characteristics of the participants suffering from chronic tension-type headache (CTTH). All subjects were female.

Patient no.	Duration of CTTH disease [years]	Prophylactic or further medication	Further Diseases
#1	Over 50	Etoricoxib or acetylsalicylic acid, if necessary No current prophylactic treatment	Keratopathy Sinonasal polyposis with sinusitis
#2	2.5	No current prophylactic treatment Ibuprofene, if necessary	Appendectomy Surgery of Hallux Valgus Herniated vertebral disc in history, no clinical symptoms Arterial hypertension
#3	5	Current prophylactic treatment with topiramate for 4 months without any success so far	None
#4	1	None	None
#5	Over 45	None	Struma
#6	2	Current prophylactic treatment with amitriptyline for 6 months without any success so far	None
#7	8	Current prophylactic treatment with topiramate and gabapentin without any success so far	Slightly increased erythrocyte sedimentation rate, no evidence for an underlying disease
#8	5	None	Tinnitus
#9	Over 15	No current prophylactic treatment Ibuprofene, if necessary	Allergic rhinitis and bronchial asthma

No motion artifacts of the MRI scans were found in the included subjects. The signal-to-noise was higher than 40 in every subject (mean 55 ± 5.5).

**Figure 1:**
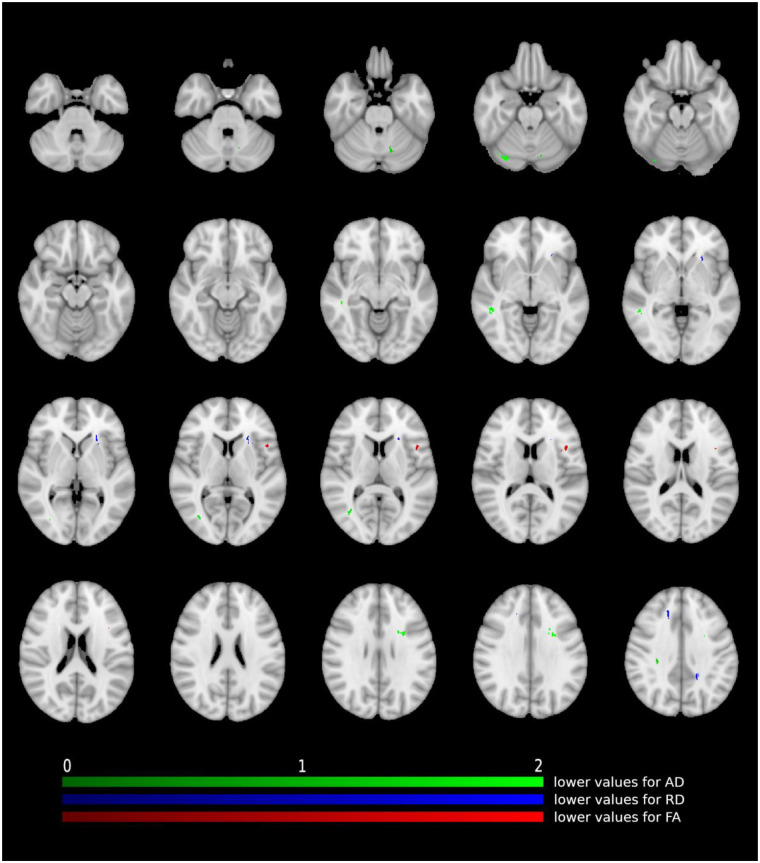
Shown is the negative decadic logarithm of the clusterwise *p*-values. The threshold for the clusters is a clusterwise *p*-value < 0.05 (>1.3 for the negative decadic logarithm). Red marked spots indicate clusters with significantly lower (negative) values for FA: Right Cerebellum^1^ and Left Inferior Frontal Gyrus^1^ (Left Superior Longitudinal Fasciculus^2^). Green marked spots indicate clusters with significantly lower (negative) values for AD: Right Middle Temporal Gyrus^1^ (Right Superior Longitudinal Fasciculus^2^), Right Occipital Fusiform Gyrus^1^, Left Middle Frontal Gyrus^1^ (Left Superior Longitudinal Fasciculus^2^), Right Postcentral Gyrus^1^, Right Lateral Occipital Cortex, inferior division^1^ (Right Inferior Longitudinal Fasciculus^2^) and Left Cerebellum. Blue marked spots indicate clusters with significantly lower (negative) values for RD: Right Right Paracingulate Gyrus^1^ (Right Cingulum^2^), Left Insular Cortex^1^ (Left Inferior fronto-occipital fasciculus^2^) and Left Precuneous^1^ (Left Cingulate Gyrus, posterior division^1,2^). ^1^Harvard-Oxford Cortical Segmentation | ^2^Johns Hopkins University Tracts Atlas.

**Figure 2:**
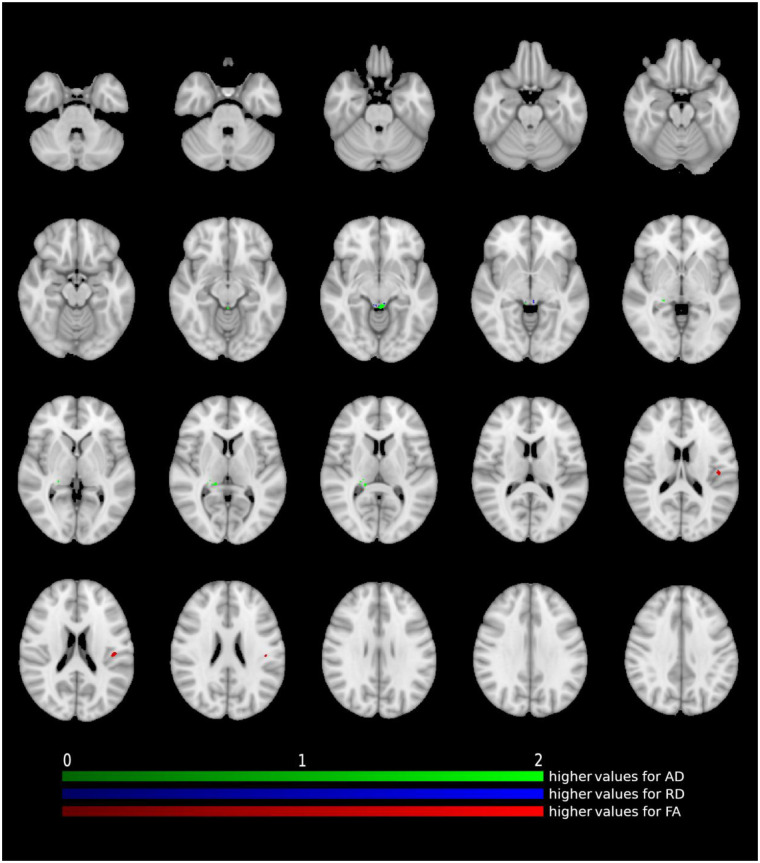
Shown is the negative decadic logarithm of the clusterwise *p*-values. The threshold for the clusters is a clusterwise *p*-value < 0.05 (>1.3 for the negative decadic logarithm). Red marked spots indicate clusters with significantly higher (positive) values for FA: Left Central Opercular Cortex^1^ (Left Superior Longitudinal Fasciculus^2^). Green marked spots indicate clusters with significantly higher (positive) values for AD: Right Anterior thalamic radiation^2^ (Right Thalamus^1^) and Right Tectum^1^. Blue marked spots indicate clusters with significantly higher (positive) values for RD: Right Tectum^1^. ^1^Harvard-Oxford Cortical Segmentation | ^2^Johns Hopkins University Tracts Atlas.

**Figure 3:**
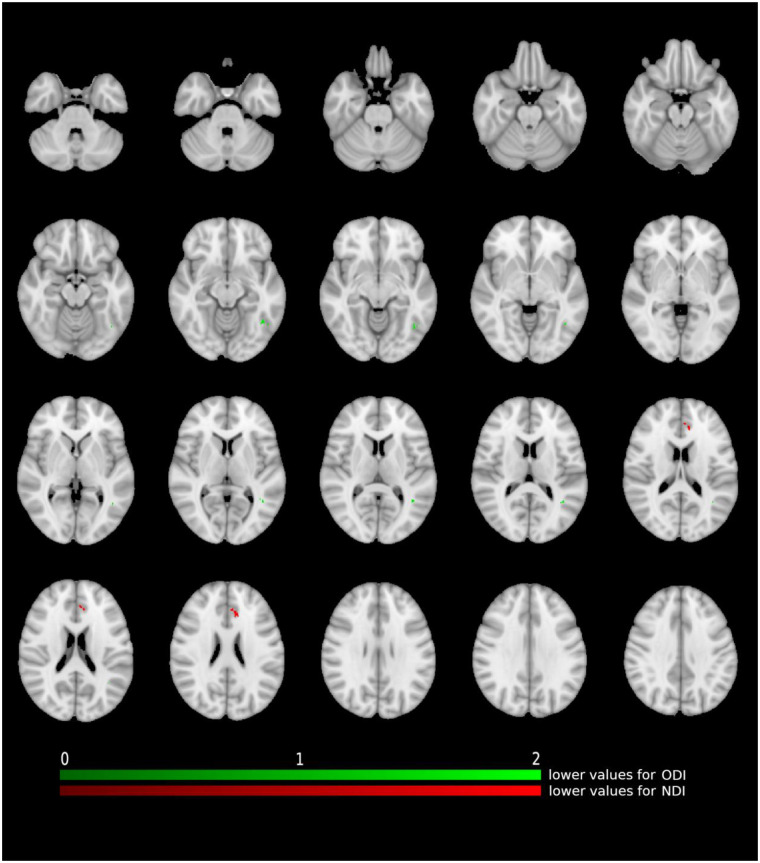
Shown is the negative decadic logarithm of the clusterwise *p*-values. The threshold for the clusters is a clusterwise *p*-value < 0.05 (>1.3 for the negative decadic logarithm). Red marked spots indicate clusters with significantly lower (negative) values for NDI: Left Cingulate Gyrus, anterior devision^1^ (Left Paracingulate Gyrus^1^). Green marked spots indicate clusters with significantly lower (negative) values for ODI: Left Middle Temporal Gyrus^1^ (Left Superior Longitudinal Fasciculus^2^) and Left Inferior Temporal Gyrus, temporooccipital part^1^ (Left Inferior Longitudinal Fasciculus^2^). ^1^Harvard-Oxford Cortical Segmentation | ^2^Johns Hopkins University Tracts Atlas.

**Figure 4:**
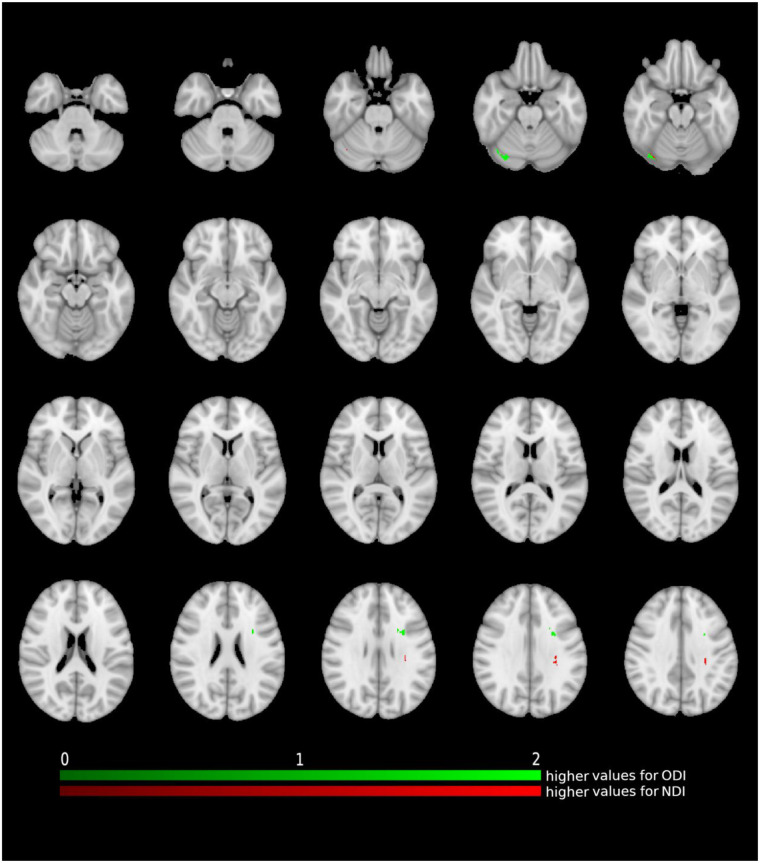
Shown is the negative decadic logarithm of the clusterwise *p*-values. The threshold for the clusters is a clusterwise *p*-value < 0.05 (>1.3 for the negative decadic logarithm). Red marked spots indicate clusters with significantly higher (positive) values for NDI: Right Occipital Fusiform Gyrus^1^ [Right Cerebellum (Crus I)^3^] and Left Superior Longitudinal Fasciculus^2^. Green marked spots indicate clusters with significantly higher (positive) values for ODI: Right Occipital Fusiform Gyrus^1^ [Right Cerebellum (Crus I)^3^] and Left Middle Frontal Gyrus^1^/Left Precentral Gyrus^1^ (Left Superior Longitudinal Fasciculus^2^). ^1^Harvard-Oxford Cortical Segmentation | ^2^Johns Hopkins University Tracts Atlas | ^3^ UCL Cerebellar Atlas.

Using different scalar indices, we were able to find microstructural changes in the right cerebellum, tectum, right occipital fusiform gyrus, left inferior temporal gyrus, left cingulate gyrus (anterior and posterior devision), right thalamic radiation, right lateral occipital cortex (inferior division), left cerebellum, left insular cortex, left precuneous, left central opercular cortex, left inferior frontal gyrus, left middle temporal gyrus, right middle temporal gyrus, left middle frontal gyrus, right postcentral gyrus, left superior longitudinal fasciculus, and the right paracingulate gyrus (for more details see Tables 3-7, Figures 1-4).

**Table 3:** Clusters with significantly higher (positive) and lower (negative) FA values in the CTTH group.

Number	Location	CWP	Volume in mm^3^	MNI 152 Coordinates		
				X	Y	Z
Positive						
1	Left Central Opercular Cortex^1^ Left Parietal Operculum Cortex^1^ Left Superior Longitudinal Fasciculus^2^ (secondary somatosensory cortex)	0.031	171	−45	−20	16
Negative						
1	Right cerebellum^3^ (VIIb and VIIIa)	0.019	180	20	−72	−37
2	Left Inferior Frontal Gyrus, pars opercularis^1^ Left Frontal Operculum Cortex^1^ Left Central Opercular Cortex^1^ Left Superior Longitudinal Fasciculus^2^ (Broca's area BA44 u BA45)	0.026	175	−43	11	10

“Location” indicates the anatomical landmark comprising the majority of voxels of a cluster according to Harvard-Oxford Cortical Segmentation^1^, Johns Hopkins University (JHU) Tracts Atlas^2^ or University College London (UCL) cerebellar atlas^3^. *P*-values are clusterwise *p*-values (CWP) corrected for multiple comparisons. “Volume in mm^3^” denotes the size of a cluster and “MNI152-coordinates” describes the coordinates of the cluster's center of gravity in MNI152-space.

**Table 4:** Clusters with significantly higher (positive) and lower (negative) ODI values in the CTTH group.

Number	Location	CWP	Volume in mm^3^	MNI 152 Coordinates		
				X	Y	Z
Positive						
1	Right Occipital Fusiform Gyrus^1^ Right Lateral Occipital Cortex, inferior division^1^ Right cerebellum (Crus I)^3^ (Visual cortex)	<0.001	277	27	−77	−17
2	Left Middle Frontal Gyrus^1^ Left Precentral Gyrus^1^ Left Superior Longitudinal Fasciculus^2^	<0.001	224	−29	7	25
Negative						
1	Left Inferior Temporal Gyrus, temporooccipital part^1^ Left Lateral Occipital Cortex, inferior division^1^ Left Inferior Longitudinal Fasciculus^2^ (Optic radiation)	0.019	142	−41	−63	−7
2	Left Middle Temporal Gyrus, temporooccipital part^1^ Left Lateral Occipital Cortex, inferior division^1^ Left Superior Longitudinal Fasciculus^2^ (Optic radiation)	0.019	142	−39	−51	6

“Location” indicates the anatomical landmark comprising the majority of voxels of a cluster according to Harvard-Oxford Cortical Segmentation^1^, Johns Hopkins University (JHU) Tracts Atlas^2^ or University College London (UCL) cerebellar atlas^3^. *P*-values are clusterwise *p*-values (CWP) corrected for multiple comparisons. “Volume in mm^3^” denotes the size of a cluster and “MNI152-coordinates” describes the coordinates of the cluster's center of gravity in MNI152-space.

**Table 5:** Clusters with significantly higher (positive) NDI values in the CTTH group.

Number	Location	CWP	Volume in mm^3^	MNI 152 Coordinates		
				X	Y	Z
Positivema						
1	Right Occipital Fusiform Gyrus^1^ Right Cerebellum (Crus I)^3^	<0.001	195	35	−75	−14
2	Left Superior Longitudinal Fasciculus^2^ Left Corticospinal tract^2^	<0.001	213	−31	−21	26
Negative						
1	Left Cingulate Gyrus, anterior devision^1^ Left Paracingulate Gyrus^1^	<0.001	217	5	33	20

“Location” indicates the anatomical landmark comprising the majority of voxels of a cluster according to Harvard-Oxford Cortical Segmentation^1^, Johns Hopkins University (JHU) Tracts Atlas^2^ or University College London (UCL) cerebellar atlas^3^. *P*-values are clusterwise *p*-values (CWP) corrected for multiple comparisons. “Volume in mm^3^” denotes the size of a cluster and “MNI152-coordinates” describes the coordinates of the cluster's center of gravity in MNI152-space.

**Table 6:** Clusters with significantly higher (positive) and lower (negative) AD values in the CTTH group.

Number	Location	CWP	Volume in mm^3^	MNI 152 Coordinates		
				X	Y	Z
Positive						
1	Right Anterior thalamic radiation^2^ Right Thalamus^1^	0.002	152	17	−34	8
2	Right Tectum	0.009	134	−1	−40	−7
Negative						
1	Right Middle Temporal Gyrus, posterior division^1^ Right Superior Longitudinal Fasciculus^2^ (Optic radiation)	<0.001	186	46	−42	−2
2	Left Middle Frontal Gyrus^1^ Left Precentral Gyrus^1^ Left Superior Longitudinal Fasciculus^3^ (Callosal body)	<0.001	185	−29	7	25
3	Right Occipital Fusiform Gyrus^1^ Right Cerebellum (Crus I)^3^	0.002	158	29	−74	−18
4	Right Lateral Occipital Cortex, inferior division^1^ Right Inferior longitudinal fasciculus^2^	0.02	121	34	−72	7
5	Right Postcentral Gyrus^1^ Right Superior Longitudinal Fasciculus ^2^ Right Corticospinal Tract^2^	0.03	116	30	−25	34
6	Left Cerebellum (VI)^3^	0.036	112	−14	−68	−20

“Location” indicates the anatomical landmark comprising the majority of voxels of a cluster according to Harvard-Oxford Cortical Segmentation^1^, Johns Hopkins University (JHU) Tracts Atlas^2^ or University College London (UCL) cerebellar atlas^3^. *P*-values are clusterwise *p*-values (CWP) corrected for multiple comparisons. “Volume in mm^3^” denotes the size of a cluster and “MNI152-coordinates” describes the coordinates of the cluster's center of gravity in MNI152-space.

**Table 7:** Clusters with significantly higher (positive) and lower (negative) RD values in the CTTH group.

Number	Location	CWP	Volume in mm^3^	MNI 152 Coordinates		
				X	Y	Z
Positive						
1	Right Tectum	<0.001	165	6	−34	−4
Negative						
1	Left Insular Cortex^1^ Left Inferior fronto-occipital fasciculus^2^	<0.001	195	−23	23	0
2	Right Paracingulate Gyrus^1^ Right Superior Frontal Gyrus^1^ Right Cingulum^2^	0.007	124	15	26	35
3	Left Precuneous^1^ Left Cingulate Gyrus, posterior division^1,2^	0.009	122	−22	−39	34

“Location” indicates the anatomical landmark comprising the majority of voxels of a cluster according to Harvard-Oxford Cortical Segmentation^1^, Johns Hopkins University (JHU) Tracts Atlas^2^ or University College London (UCL) cerebellar atlas^3^. *P*-values are clusterwise *p*-values (CWP) corrected for multiple comparisons. “Volume in mm^3^” denotes the size of a cluster and “MNI152-coordinates” describes the coordinates of the cluster's center of gravity in MNI152-space.

## 4 Discussion

To our knowledge, this is the first attempt to detect cerebral microstructural changes in patients suffering from CTTH using DTI. Our study has an exploratory approach and showed changes in both—gray and white matter—across different brain regions, many of which are components of or closely connected to the pain matrix and key associative fiber tracts.

### 4.1 Main findings

Notably, most alterations were found in regions that contribute to the ipsilateral superior longitudinal fasciculus (SLF) or the inferior longitudinal fasciculus (ILF), respectively (see Tables 3–7). Our findings of reduced axonal diffusivity (AD) in the SLF or right occipital fusiform cortex may indicate regional axonal loss or damage (28, 56, 57), among other possible interpretations (58). Also, in early demyelination a decrease of AD was evident which was no longer observed in prolonged demyelination (59). We also observed increased radial diffusivity (RD) in the tectum, which could be potentially an indicator for demyelination (48). However, there are several ways to interpret an increase in RD. Another explanation is an increase of the amount of free water in the interstitial space (60) due to glial cell activation (61) which may occur in neuroinflammation or injury (59) or due to neural plasticity (62). Changes in AD and RD reflect alterations in water diffusion properties in the first line, which can indicate microstructural changes of different origins.

There were microstructural changes in the right paracingulate cortex, right cerebellar lesions and changes in the tectum that might include the superior and inferior colliculi. The positive AD and RD values found in our study may reflect axonal or myelin alterations of the superior and inferior colliculi.

### 4.2 Interpretation and comparison with prior studies

SLF and ILF support multisensory integration and connect cortical areas involved in nociceptive processing and modulation. Frontal and parietal brain areas are connected by the SLF, its main functions include language processing or visuospatial attention (63, 64). The ILF ensures connections between amyg­dala, hippocampus and parahippocampal regions. By this, the ILF affects various brain functions such as emotional response, spatial perception or visual memory (56, 65) and might contribute to the pathophysiology of headache disorders by influencing nociceptive pathways (25). Prior studies have reported similar DTI changes of the SLF and ILF in chronic headache disorders, such as new daily persistent headache (NDPH) (25, 26), migraine (24) or chronic migraine with a history of medication abuse (66). In patients suffering from depression or anxiety, microstructural lesions could be found in the SLF and ILF (19, 24), too. Li et al. stated that a disruption of the connection between the ILF and the amygdala might be responsible for panic attacks observed in some patients with NDPH (26).

The ILF functionally connects the parahippocampal cortex with limbic structures implicated in pain perception and emotional regulation (65). DTI alterations of the ILF have also been reported in migraine and are linked to attack frequency (67). As a part of the limbic system, there are projections from parahippocampal brain areas to the cingulum responsible for motivation, emotion and pain perception (68, 69). Parts of the cingulum belong to the pain matrix, a network of cortical and subcortical brain areas modulating nociceptive stimuli (70, 71). Recent studies demonstrated microstructural changes of the cingulum in migraine (66), cluster headache (72) and NDPH (55) suggesting an important role of the cingulum in nociceptive pathways in headache disorders. In line with this we found DTI changes in the right paracingulate cortex and the left cingulate gyrus (anterior and posterior devision/precuneous). These regions are also a part of brain regions known to be disturbed in depressed patients (73, 74). In a recent metaanalysis, Zhong et al. described alterations of the fronto-limbic circuit in drug-naive patients with a major depressive disorder using functional MRI (75). These changes included the precuneus, posterior cingulate gyrus as well as fusiform gyrus in which we found DTI-changes, too.

The thalamus is part of the pain matrix (70), and thalamic DTI alterations have been decribed for headache disorders such as migraine (24) and cluster headache (76). The role of the thalamus in the pathophysiology of TTH has also been discussed (77, 78). Consistent with these findings, we were able to demonstrate microstructural changes in the thalamus in our study.

The detected cerebellar microstructural lesions, particularly in the right hemisphere, align with previous findings in cluster headache (23) or in migraineurs using DTI (79, 80). Beyond motor control, the cerebellum is increasingly recognized as being part of the pain matrix (70), and provides a motoric answer to pain (81). In addition to this, recent studies suggest connections between the cerebellum and brain regions that control cognitive and emotional processing. By this, cerebellar systems can influence nociception in acute and chronic pain (82) as well as in headache disorders such as migraine (83). CGRP and its receptors are present in cerebellar neurons (84). Additionally, our observed changes in the tectum might include the superior and inferior colliculi that are both connected to the trigeminal system (85, 86). Thus, there might be possible CGRP-dependent involvement of midbrain sensory processing structures in CTTH as they are known to contain CGRP-positive neurons (87, 88). Using DTI, microstructural alterations of the superior colliculi were reported for migraine (89), too. The conclusions drawn from these observations are speculative, but they could support the hypothesis of a potential mechanistic link in CTTH, as CGRP is a key neuropeptide of the trigeminal system that plays an important pathophysiological role in primary headache disorders as mentioned above. This has been demonstrated in particular for migraine (15, 90), but not yet for CTTH. In fact, recent studies demonstrated normal CGRP levels in plasma or cerebrospinal fluid (17, 91). Nevertheless, Ashina stated that these findings do not exclude the possibility that abnormalities of CGRP at neuronal or peripheral levels play a role in the pathophysiology of chronic tension-type headache (18).

In line with the findings of Schmidt-Wilcke et al. (16), we observed microstructural changes in the right cerebellum, and the anterior as well as posterior devision of the cingulate gyrus as parts of the pain matrix. However, we could not find lesions in all areas reported in their study. This discrepancy may reflect methodological differences: VBM primarily captures gray-matter volume changes, whereas DTI is particularly sensitive to white-matter microstructure. Using DTI in other headache disorders, some of the microstructural alterations found in our study have also been reported in migraine (24) or NDPH (25, 26), including changes in the SLF, ILF, and middle frontal gyrus. These changes, however, could not be found by Schmidt-Wilcke et al. (16).

### 4.3 Implications

Our findings indicate that changes in microstructure of gray and white matter occur in patients with CTTH.

The microstructural changes in the SLF, ILF, and right cerebellum suggest that, in addition to sensory and nociceptive systems, areas involved in the emotional evaluation of pain, language processing, spatial or visual memory might be also affected in CTTH. In addition to this, we might assume central nociceptive processing abnormalities in CTTH according to changes in the thalamus, paracingulate and cingulate cortex in our study.

Interestingly, we found microstructural abnormalities in central CGRP-containing regions, such as the cerebellum and tectum with the superior and inferior colliculi. This could support the hypothesis that CGRP might play a role in the pathophysiology of CTTH, even though clinical studies did not yield clear results as mentioned above.

### 4.4 Limitations

The main limitation of this study is the small, all-female sample (*n* = 9), which precludes generalization and correlation analyses with clinical variables, e.g., the duration of CTTH, gender or age of the subjects, especially since the age of the participants and their duration of CTTH varied. However, the small sample size is due to our strict inclusion criteria aimed at reducing confounding factors, in particular any comorbidities or analgetic overuse.

As mentioned above, women are more likely to suffer from CTTH than men. This epidemiological factor might—at least in part—explain why only female participants could be included in our study. However, since gender may affect DTI results (92), our conclusions cannot be applied to male CTTH patients. Furthermore, female sex hormones are able to influence pain and nociception via complex mechanisms (93). In migraine, for example, a regulation of serotonin and CGRP levels is discussed (94). In addition to this, gonadal hormones can alter white matter microstructure during menstrual cycle in healthy volunteers (95). Future research should specifically include male participants.

Age and disease duration might influence DTI changes, too, as described in other pain disorders. Chen et al. could demonstrate altered microstructure integrity of the white matter in multiple brain regions in patients suffering from postherpetic neuralgia. Using DTI, these changes depended on the duration of the disease (96). In migraineurs, Chong and Schwedt found alterations of the white-matter integrity. Longer migraine history was positively correlated with greater alterations in tract integrity (97). In our study, the age and the duration of CTTH varied, which might influence our results. However, we included the patients' age as a confounding variable in the GLM.

The need for correction for multiple testing when examining several scalar maps derived from the diffusion tensor remains a matter of debate in the literature (98). As in many previous studies (21, 99, 100), no additional correction was applied in this exploratory approach. This limitation should be addressed in future examinations with larger cohorts. Because of the small sample size, we decided not to conduct a correlation analysis and to focus on the group analysis. A correlation analysis between imaging indices and clinical or behavioral variables would be of great interest for future investigations.

The DTI scan has a rather coarse resolution of 2 × 2 × 2.4 mm^3^ and only one shell was obtained, therefore it was not possible to conduct a full NODDI analysis. We decided to conduct the approximation NODDI-DTI (51), which can be calculated from one shell. But this method is less accurate in comparison to full NODDI.

Three patients were taking prophylactic medication (amitriptyline, gabapentin, topiramate). In patients with depression, it has been shown that antidepressants led to microstructural changes in the brain (101, 102), even after a short treatment period (103). Therefore, we cannot rule out the possibility that amitriptyline, gabapentin, or topiramate may also have influenced our results. However, to our knowledge, there is no consistent evidence that these drugs substantially affect DTI metrics. Furthermore, Schmidt-Wilcke included subjects with a prophylactic treatment, too (16).

### 4.5 Conclusions and perspectives

In conclusion, we found microstructural changes in multiple brain regions in patients suffering from CTTH. They were related to or interact intensively with the pain matrix, trigeminal system or association fibers and may influence pain processing, emotional response as well as language or spatial memory as discussed above. These changes could not only be important in the pathophysiology of pain in CTTH but may explain the increased incidence of anxiety and depression in CTTH as stated above. They may also be associated with neurocognitive dysfunctions as we found microstructural lesions in the left middle frontal gyrus that play a role in working memory and executive functions and error awareness (104–107). Furthermore, the SLF connects Broca's with Wernicke's area suggesting that especially the left SLF might be crucial for language processing. Alterations of the left SLF may therefore be responsible for disturbances in verbal memory and skills as described for NDPH patients (26, 108, 109). Further research, similar to studies conducted on fibromyalgia (104), would be beneficial to determine whether neurocognitive disturbances are present in CTTH.

Zhang et al. (110) investigated differences in gray matter volume in patients suffering from trigeminal neuralgia. They identified three altered fronto-limbic circuits associated with clinical pain duration and emotional state rating. The microstructural abnormalities in frontal regions (e.g., the left middle frontal gyrus, inferior frontal gyrus, right paracingulate and left cingulate gyrus) found in our study might support the hypothesis of disrupted prefrontal-limbic regulation in CTTH as previously described for depression and anxiety. This might have translational implications for targeted therapies such as neuromodulatory interventions. High-frequency, repetitive transcranial magnetic stimulation (rTMS) has demonstrated efficacy in both chronic pain and depression (111, 112). First studies using rTMS or transcranial direct current stimulation (tDCS) were performed in CTTH and showed promising results (113, 114). As mentioned above, our study is small and exploratory in nature. Ultimately, our results cannot determine whether the reported changes are a cause or a consequence of CTTH. In particular, longitudinal studies are needed to address this question. Our results should serve as a basis for further research to confirm our results and to correlate microstructural changes with clinical parameters.

## Data Availability

The original contributions presented in the study are included in the article/Supplementary Material, further inquiries can be directed to the corresponding author.
