# Supplementary material for: Diffusion tensor imaging in chronic tension-type headache
Source: Front Pain Res (Lausanne). 2026 Jul 1;7:1850836. doi: 10.3389/fpain.2026.1850836 (PMC13368757; doi:10.3389/fpain.2026.1850836)

Appendix - Supplementary figures

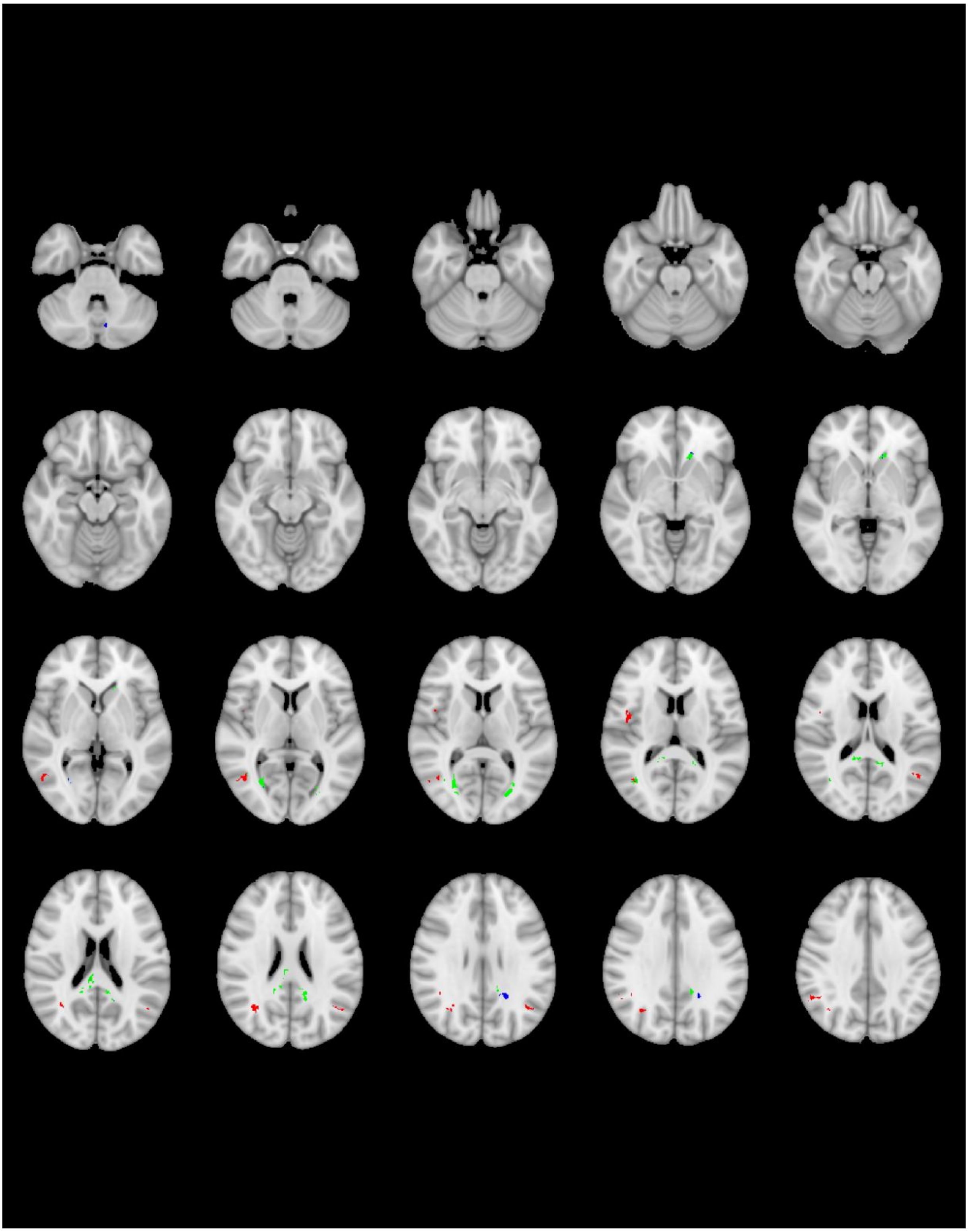

Figure 1 Shown is the negative decadic logarithm of the clusterwise p-values. The threshold for the clusters is a clusterwise p-value < 0.05 (>1.3 for the negative decadic logarithm).
Red marked spots indicate clusters with a positive correlation of the FA with the hours with headache per month.
Green marked spots indicate clusters with a positive correlation of the AD with the hours with headache per month.
Blue marked spots indicate clusters with a positive correlation of the RD with the hours with headache per month.

FA

**Positive Cluster: 1**	
Name	Unknown
CWP	0.00008
Size	292 Voxel
TalX, TalY, TalZ	51.14, -63.12, 4.9
**Linear Regression**	
Slope	0.00053
Intercept	0.06516
R-value	0.61231
P-Value	0.07964
**Spearman Regression**	
Spearman Correlation	0.61667
P-Value	0.07693

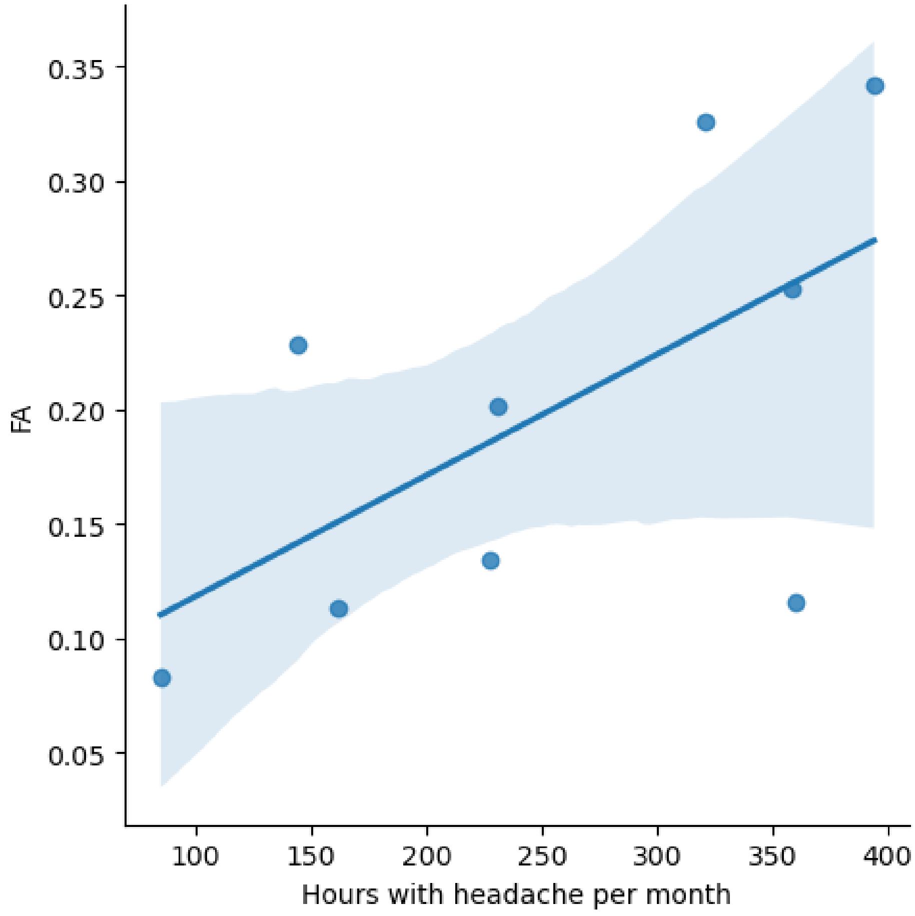

Positive Cluster: 2

Name	Right-Cerebral-White-Matter
CWP	0.00008
Size	262 Voxel
TalX, TalY, TalZ	34.92, -58.97, 23.92
**Linear Regression**	
Slope	0.00029
Intercept	0.39860
R-value	0.47411
P-Value	0.19727
**Spearman Regression**	
Spearman Correlation	0.41667
P-Value	0.26459

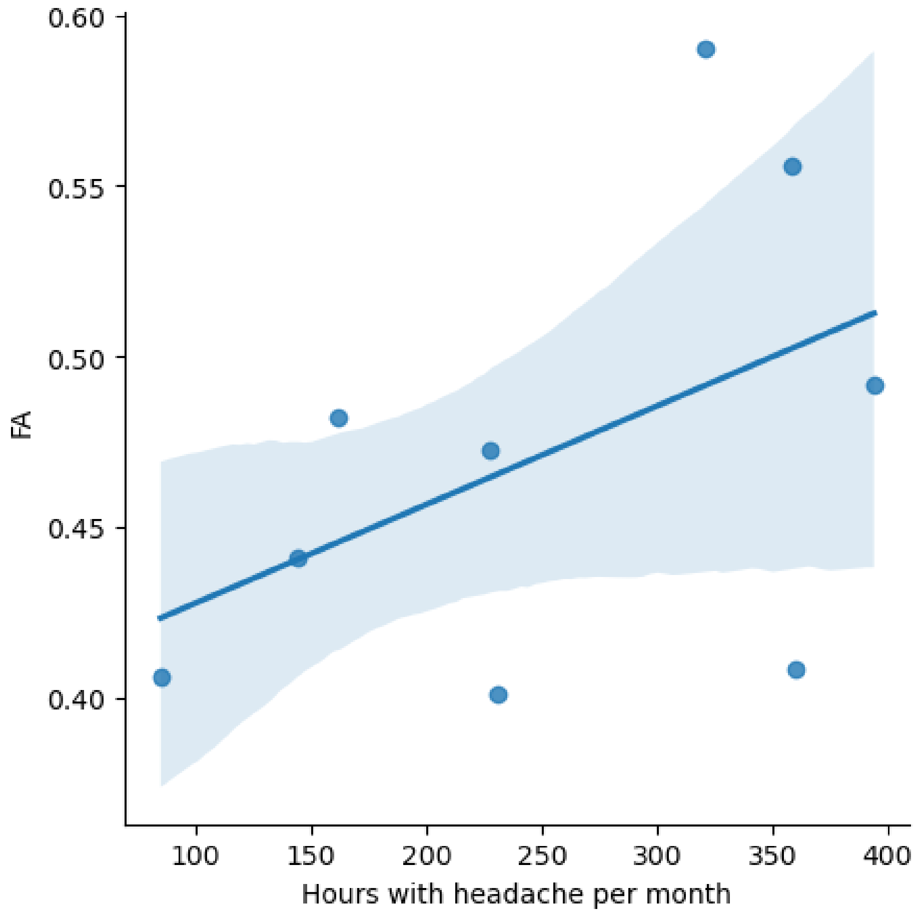

Positive Cluster: 3

Name	Left-Cerebral-White-Matter
CWP	0.00042
Size	218 Voxel
TalX, TalY, TalZ	-17.79, -8.48, 47.06
**Linear Regression**	
Slope	0.00024
Intercept	0.50471
R-value	0.43023
P-Value	0.24772
**Spearman Regression**	
Spearman Correlation	0.41667
P-Value	0.26459

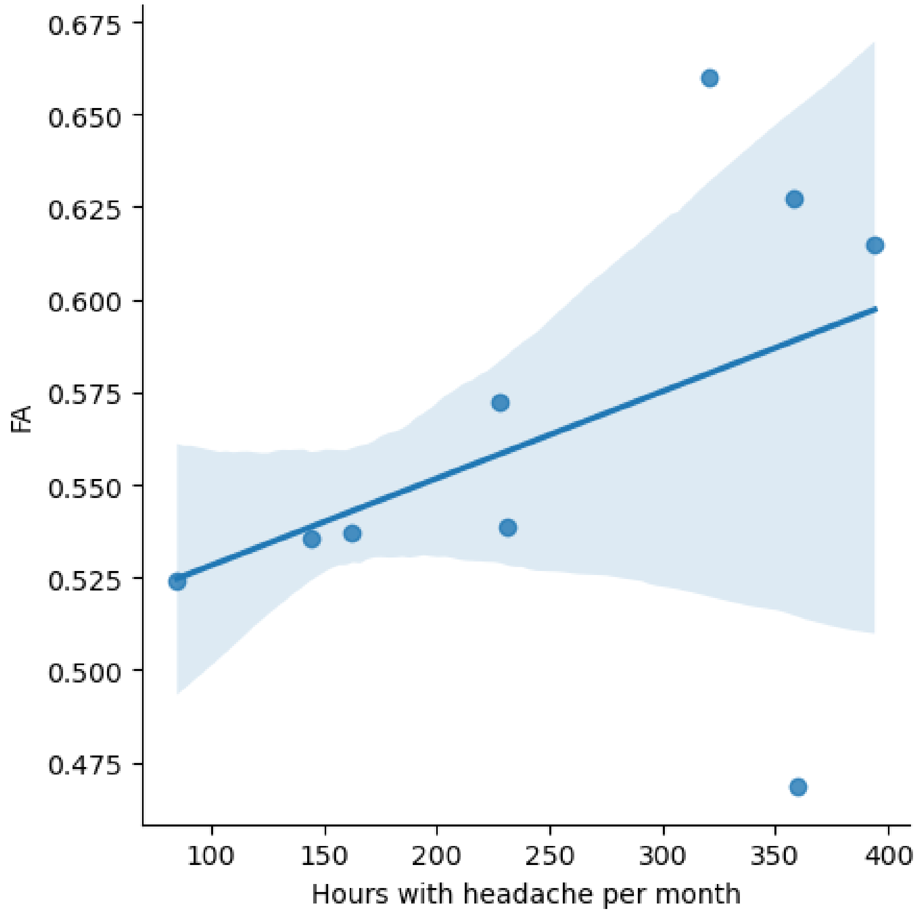

Positive Cluster: 4

Name	Right-Cerebral-White-Matter
CWP	0.00117
Size	204 Voxel
TalX, TalY, TalZ	54.68, -47.13, 32.91
**Linear Regression**	
Slope	0.00032
Intercept	0.10602
R-value	0.45048
P-Value	0.22366
**Spearman Regression**	
Spearman Correlation	0.35000
P-Value	0.35582

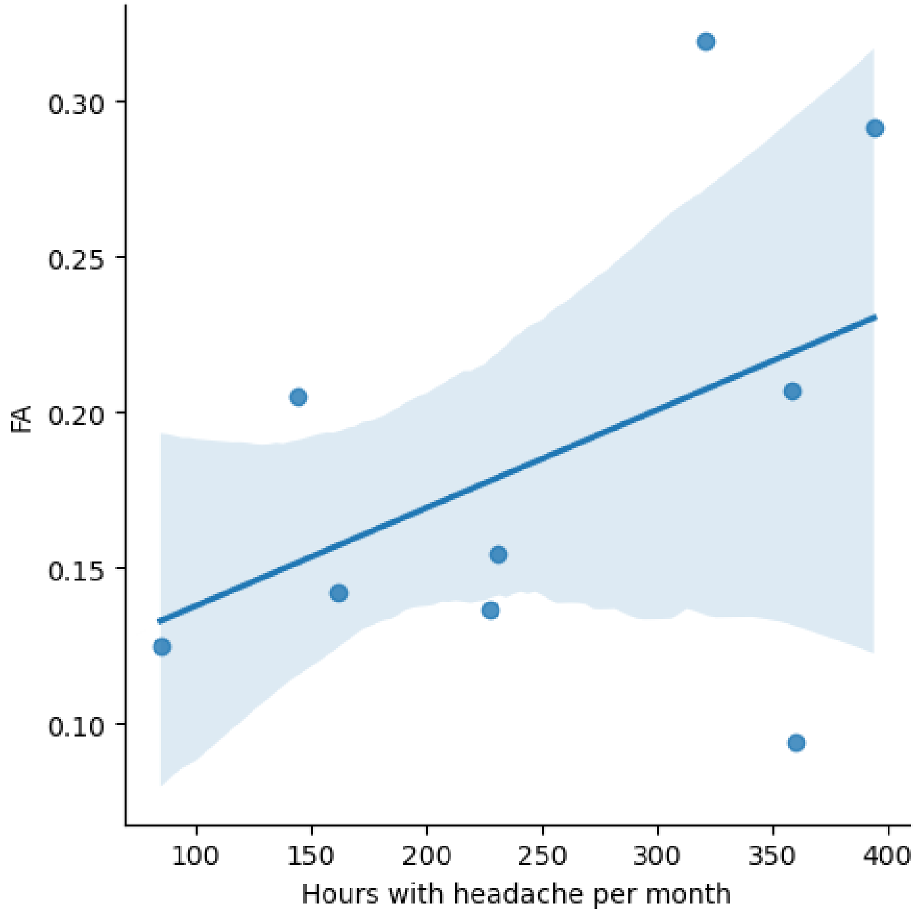

Positive Cluster: 5

Name	Right-Cerebral-White-Matter
CWP	0.01025
Size	166 Voxel
TalX, TalY, TalZ	46.4, -4.37, 13.5
**Linear Regression**	
Slope	0.00023
Intercept	0.07488
R-value	0.55018
P-Value	0.12483
**Spearman Regression**	
Spearman Correlation	0.43333
P-Value	0.24395

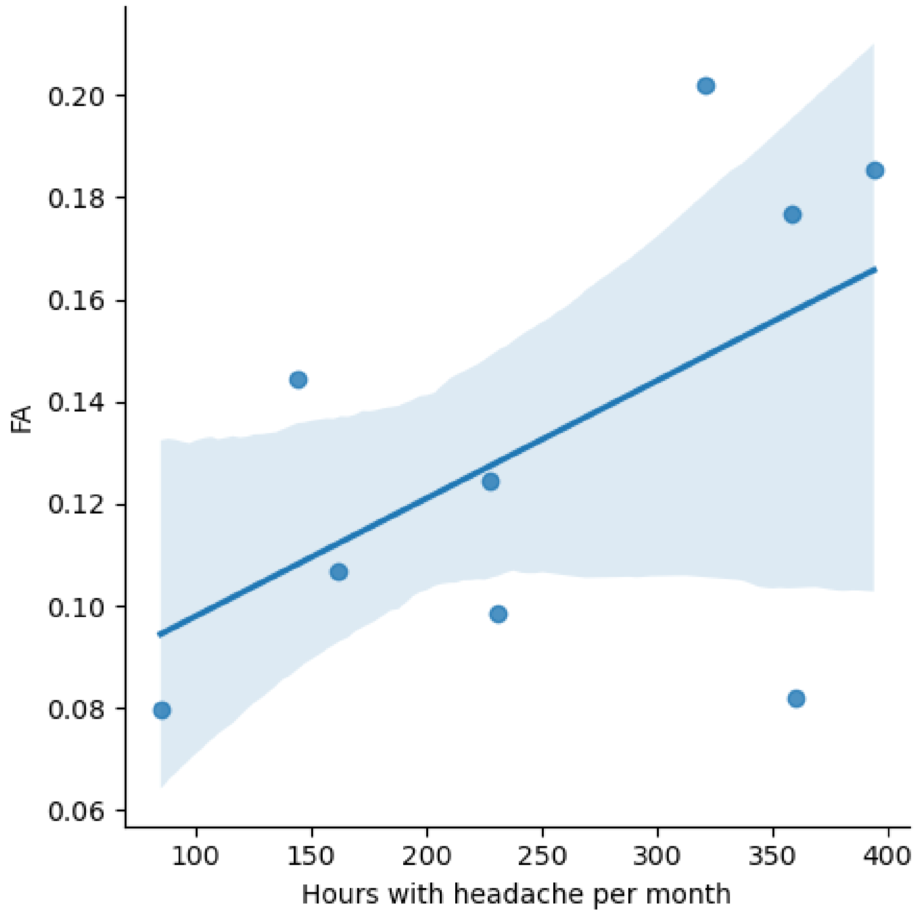

Positive Cluster: 6

Name	Unknown
CWP	0.01025
Size	166 Voxel
TalX, TalY, TalZ	-41.53, -58.75, 25.76
**Linear Regression**	
Slope	0.00040
Intercept	0.04818
R-value	0.49660
P-Value	0.17386
**Spearman Regression**	
Spearman Correlation	0.31667
P-Value	0.40640

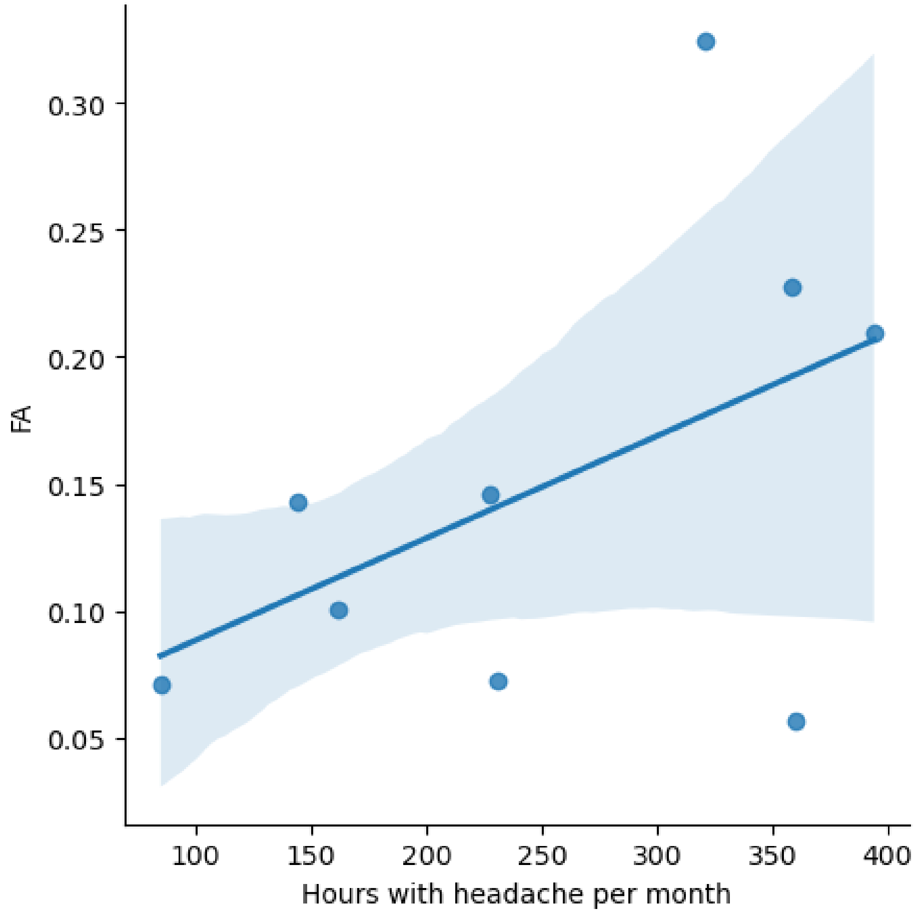

AD

**Positive Cluster: 1**	
Name	Left-Cerebral-White-Matter
CWP	0.00008
Size	317 Voxel
TalX, TalY, TalZ	-15.86, -18.23, 46.57
**Linear Regression**	
Slope	0.00043
Intercept	0.94032
R-value	0.54690
P-Value	0.12755
**Spearman Regression**	
Spearman Correlation	0.36667
P-Value	0.33174

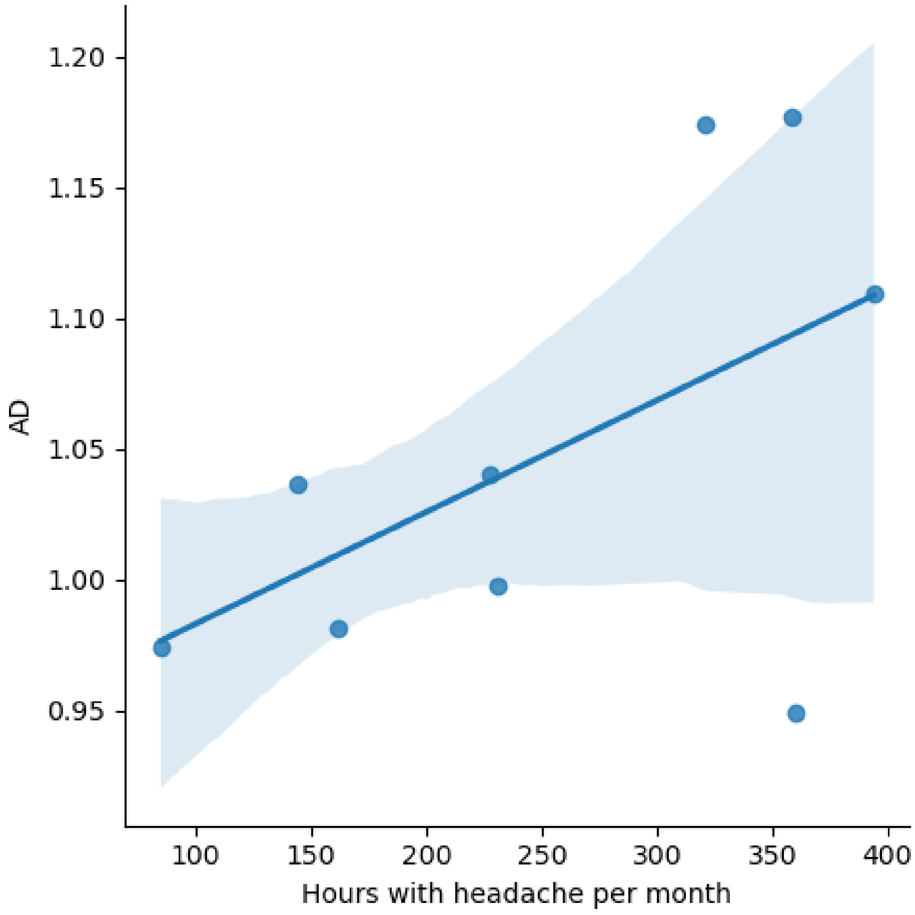

Positive Cluster: 2

Name	Left-Cerebral-White-Matter
CWP	0.00008
Size	281 Voxel
TalX, TalY, TalZ	-14.61, -50.6, 22.06
**Linear Regression**	
Slope	0.00035
Intercept	1.19208
R-value	0.36906
P-Value	0.32835
**Spearman Regression**	
Spearman Correlation	0.36667
P-Value	0.33174

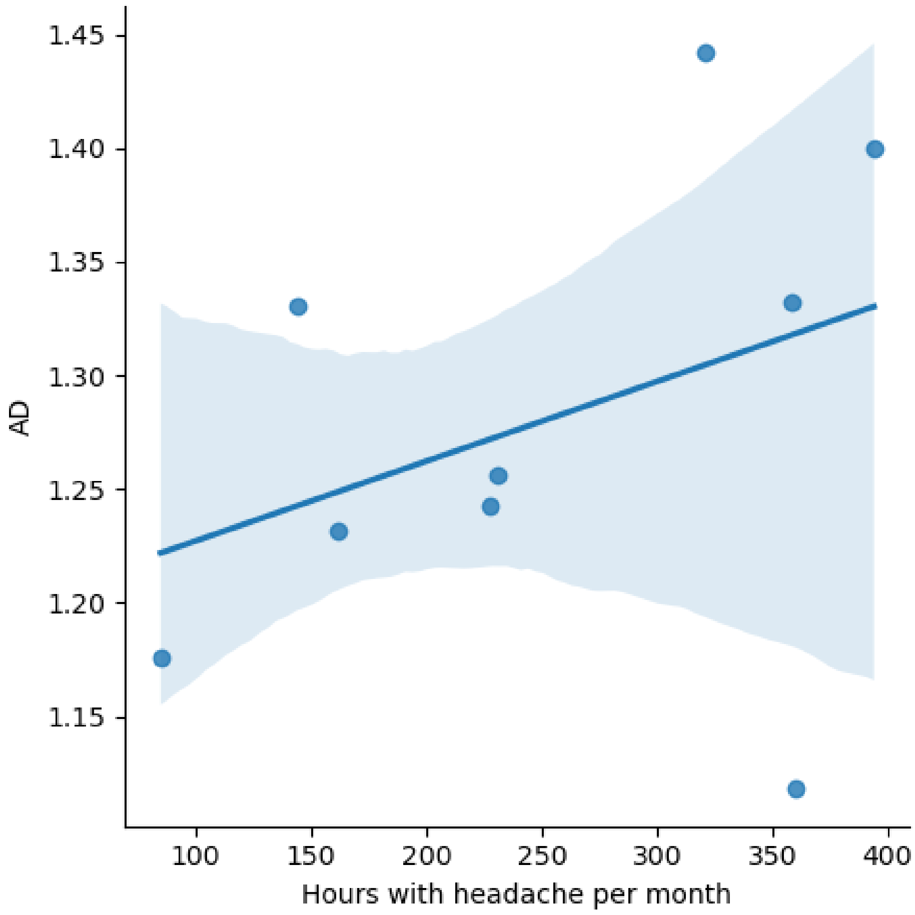

Positive Cluster: 3

Name	Right-Cerebral-White-Matter
CWP	0.00008
Size	244 Voxel
TalX, TalY, TalZ	26.27, -67.52, 6.63
**Linear Regression**	
Slope	0.00276
Intercept	0.78696
R-value	0.62328
P-Value	0.07292
**Spearman Regression**	
Spearman Correlation	0.46667
P-Value	0.20539

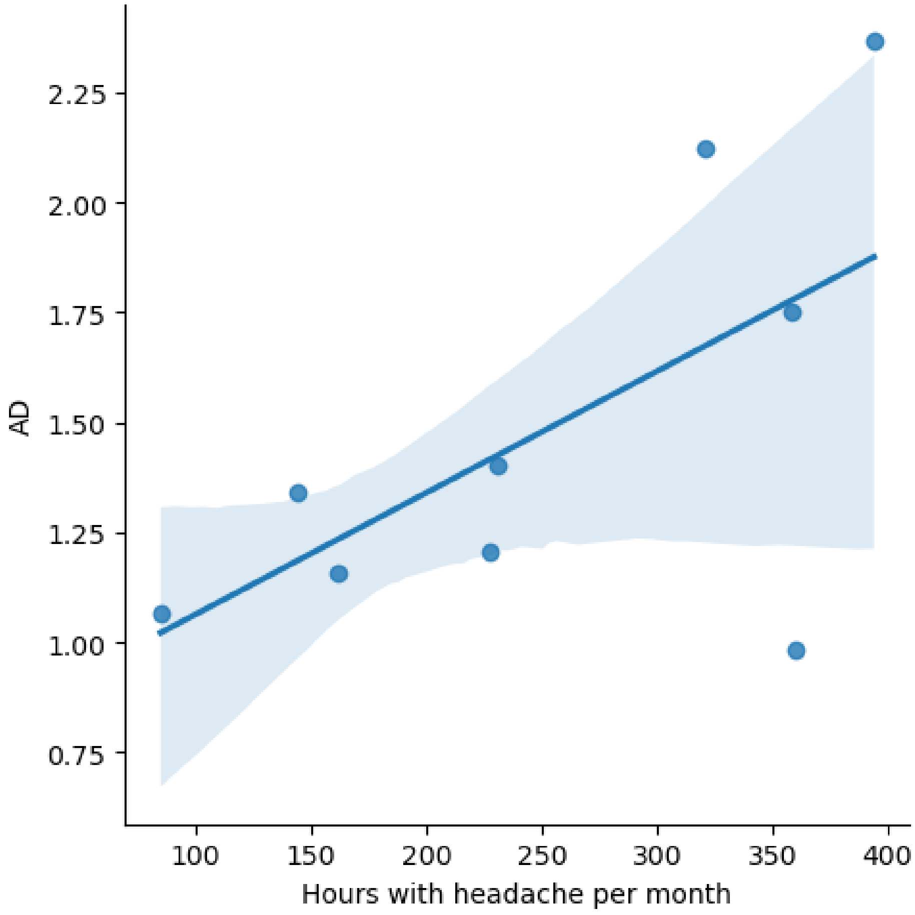

Positive Cluster: 4

Name	Right-Cerebral-White-Matter
CWP	0.00008
Size	198 Voxel
TalX, TalY, TalZ	10.39, -41.59, 15.52
**Linear Regression**	
Slope	0.00075
Intercept	1.37680
R-value	0.58763
P-Value	0.09612
**Spearman Regression**	
Spearman Correlation	0.43333
P-Value	0.24395

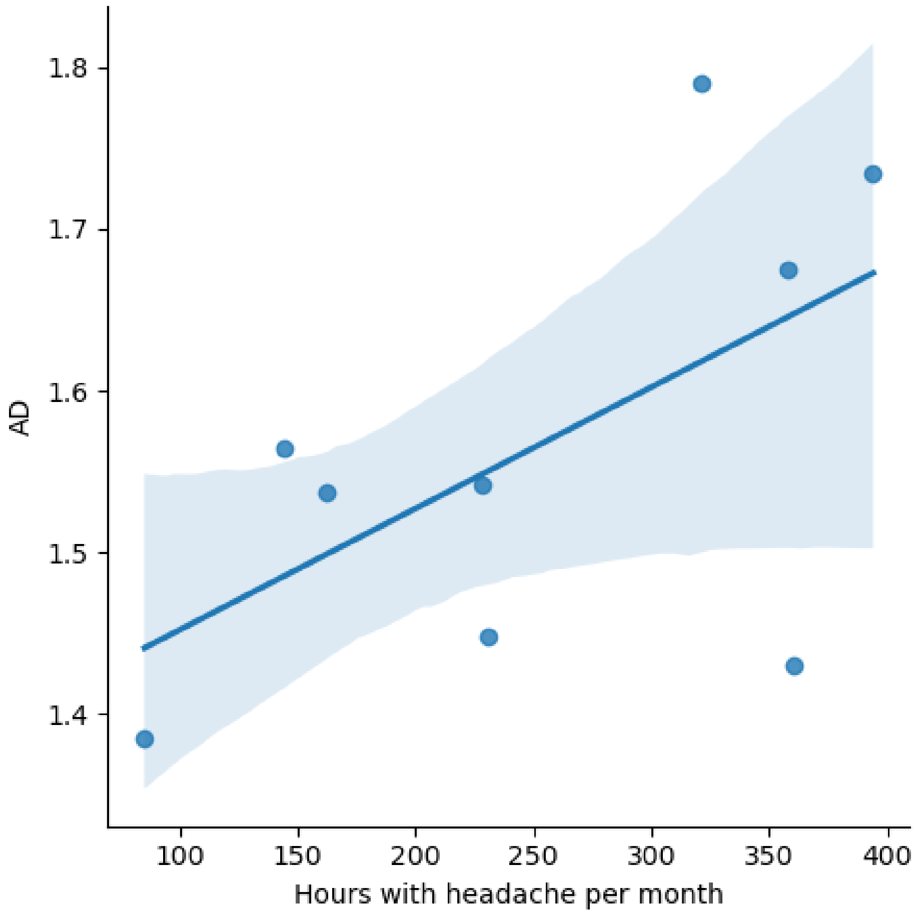

Positive Cluster: 5

Name	Left-Lateral-Ventricle
CWP	0.00033
Size	159 Voxel
TalX, TalY, TalZ	-12.58, 22.6, -6.3
**Linear Regression**	
Slope	0.00186
Intercept	1.35805
R-value	0.40816
P-Value	0.27546
**Spearman Regression**	
Spearman Correlation	0.36667
P-Value	0.33174

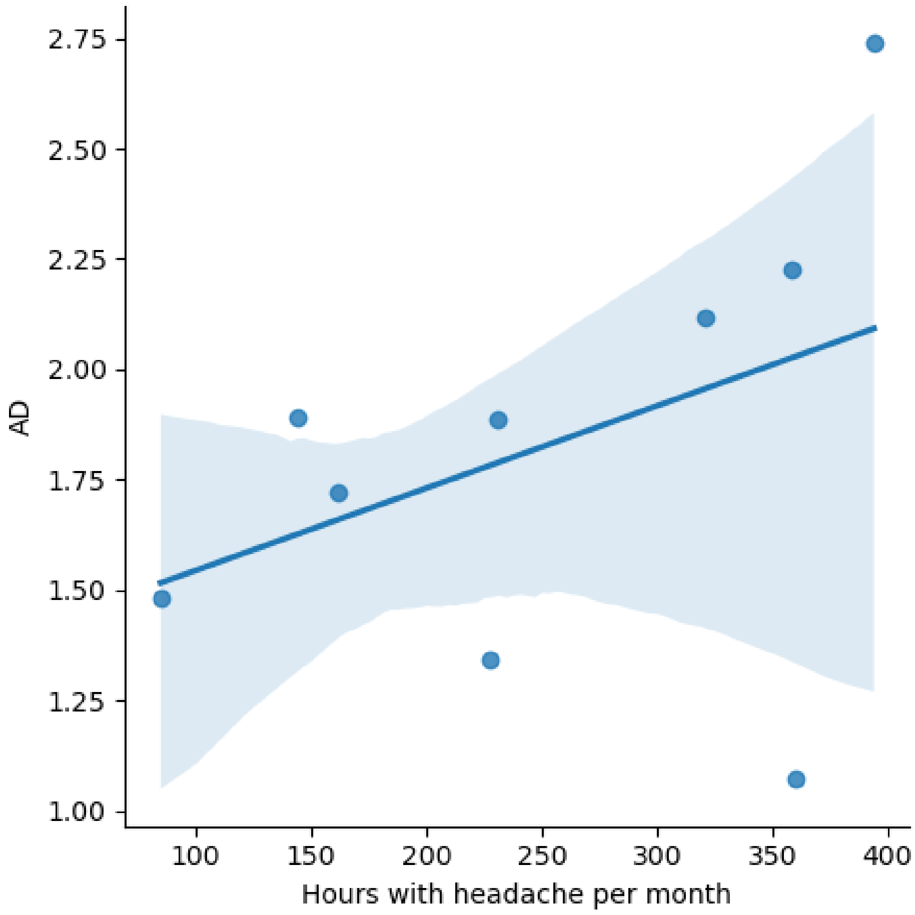

Positive Cluster: 6

Name	Left-Cerebral-White-Matter
CWP	0.00150
Size	140 Voxel
TalX, TalY, TalZ	-21.49, -75.39, 10.07
**Linear Regression**	
Slope	0.00138
Intercept	0.99567
R-value	0.38912
P-Value	0.30062
**Spearman Regression**	
Spearman Correlation	0.25000
P-Value	0.51649

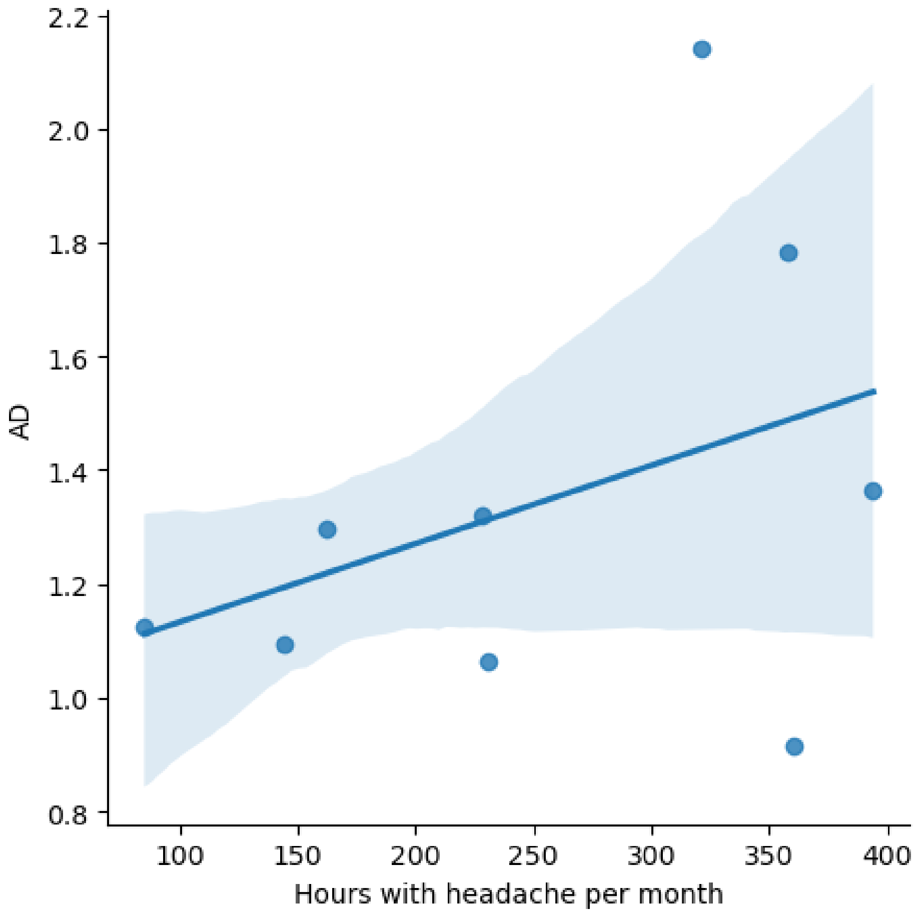

Positive Cluster: 7

Name	Right-Cerebral-White-Matter
CWP	0.01508
Size	111 Voxel
TalX, TalY, TalZ	6.4, -31.61, 19.68
**Linear Regression**	
Slope	0.00083
Intercept	1.43975
R-value	0.64132
P-Value	0.06266
**Spearman Regression**	
Spearman Correlation	0.50000
P-Value	0.17047

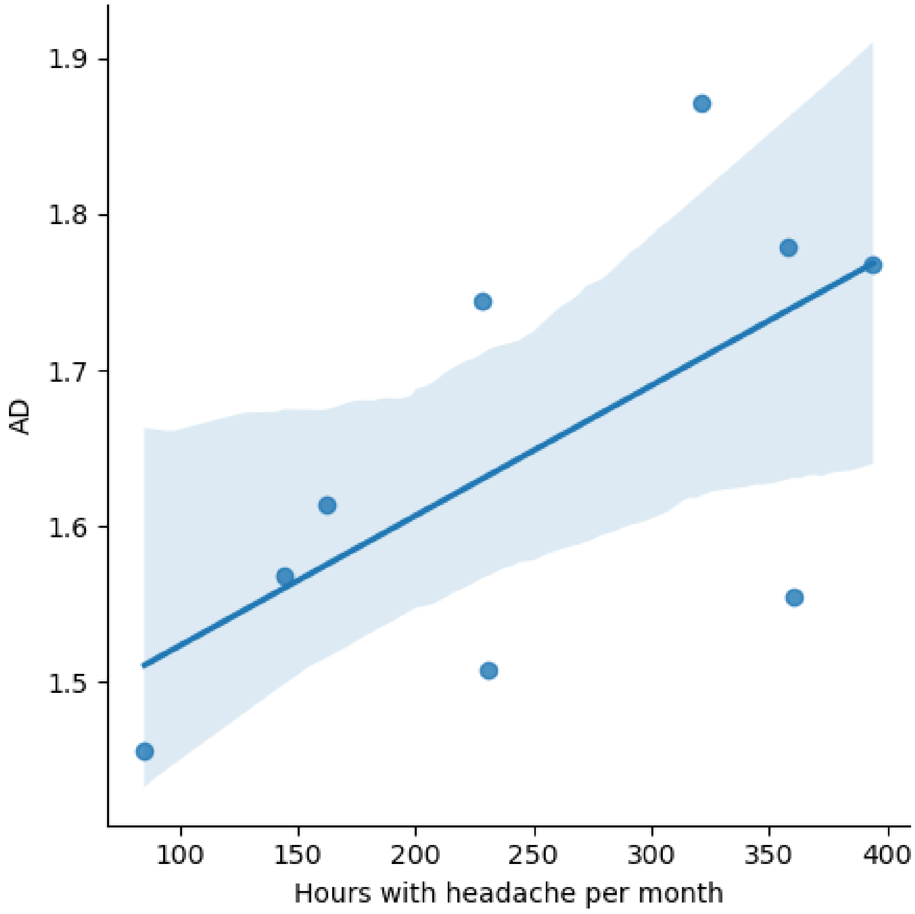

Positive Cluster: 8

Name	Right-Cerebral-White-Matter
CWP	0.02142
Size	107 Voxel
TalX, TalY, TalZ	40.19, -58.9, 8.29
**Linear Regression**	
Slope	0.00050
Intercept	0.86446
R-value	0.53540
P-Value	0.13741
**Spearman Regression**	
Spearman Correlation	0.33333
P-Value	0.38071

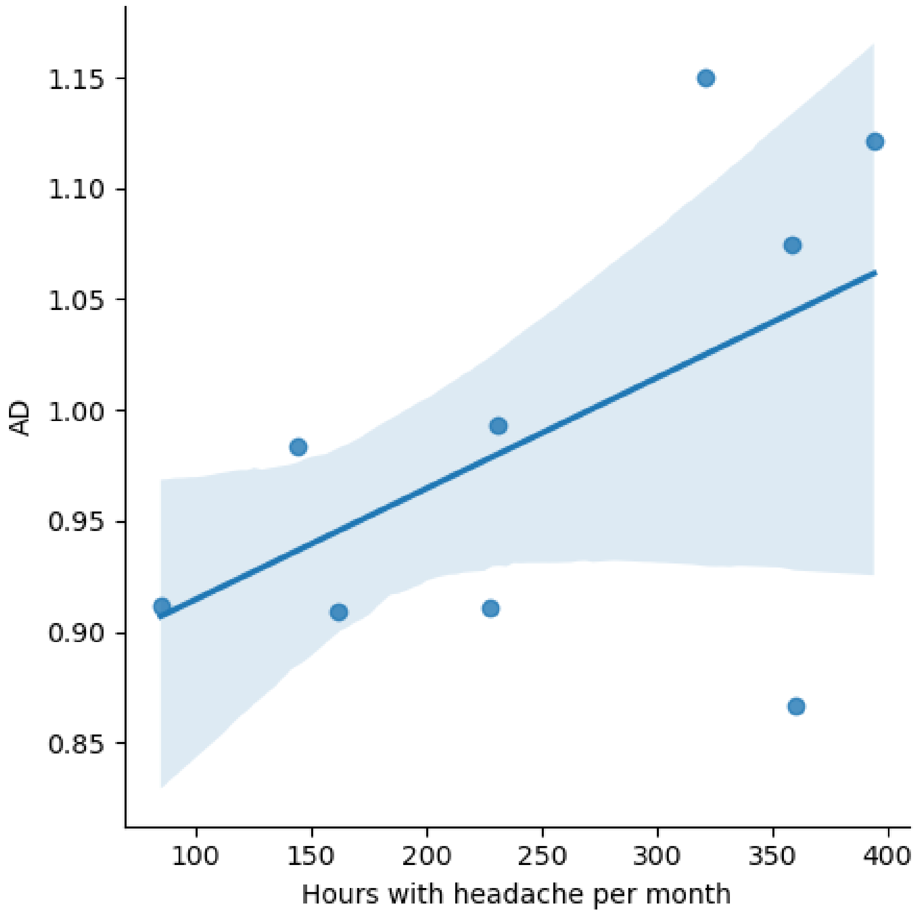

RD

**Positive Cluster: 1**	
Name	Left-Caudate
CWP	0.00008
Size	163 Voxel
TalX, TalY, TalZ	-12.57, 21.59, -7.1
**Linear Regression**	
Slope	0.00175
Intercept	1.00702
R-value	0.42777
P-Value	0.25074
**Spearman Regression**	
Spearman Correlation	0.41667
P-Value	0.26459

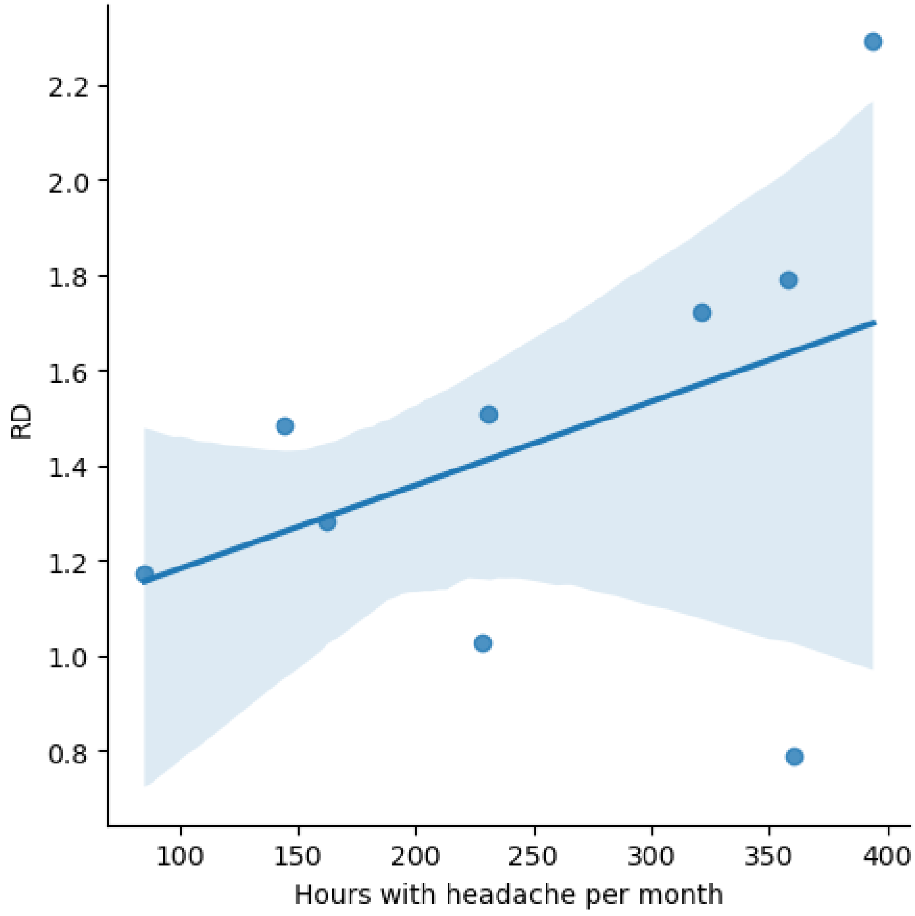

**Positive Cluster: 2**	
Name	Left-Cerebral-White-Matter
CWP	0.00008
Size	156 Voxel
TalX, TalY, TalZ	-21.65, -46.29, 28.26
**Linear Regression**	
Slope	0.00043
Intercept	0.44830
R-value	0.64554
P-Value	0.06040
**Spearman Regression**	
Spearman Correlation	0.51667
P-Value	0.15439

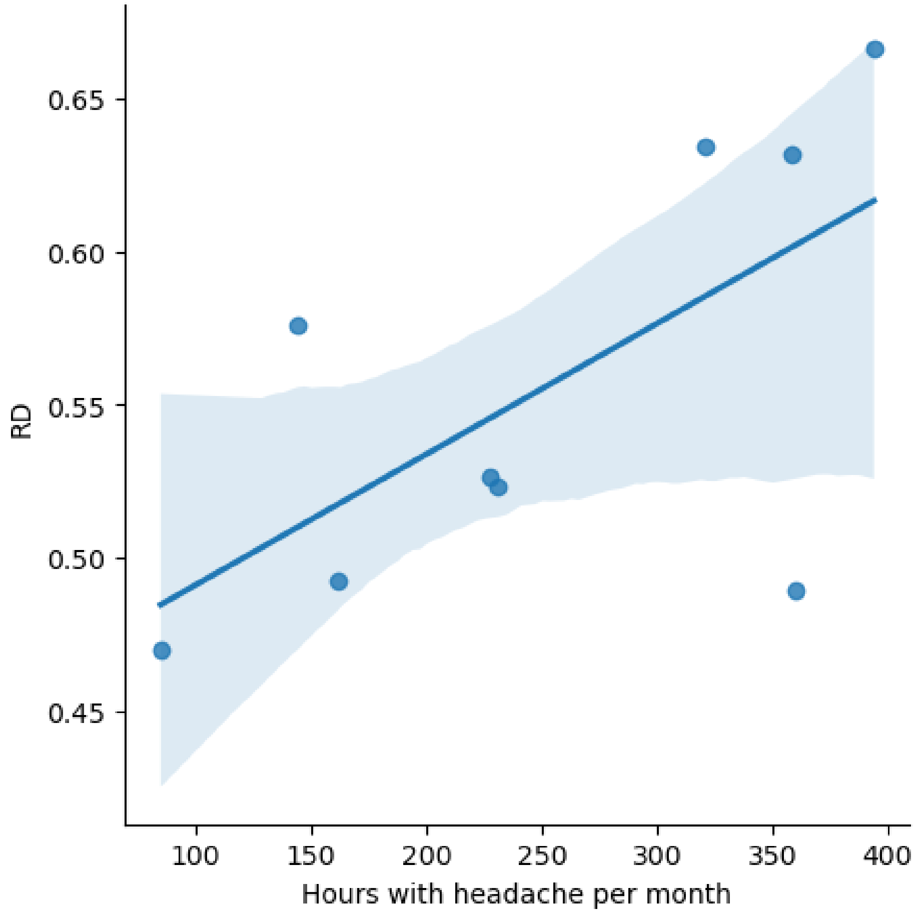

Positive Cluster: 3

Name	Right-Cerebral-White-Matter
CWP	0.00433
Size	113 Voxel
TalX, TalY, TalZ	27.17, -68.26, 11.28
**Linear Regression**	
Slope	0.00209
Intercept	0.39761
R-value	0.53556
P-Value	0.13727
**Spearman Regression**	
Spearman Correlation	0.35000
P-Value	0.35582

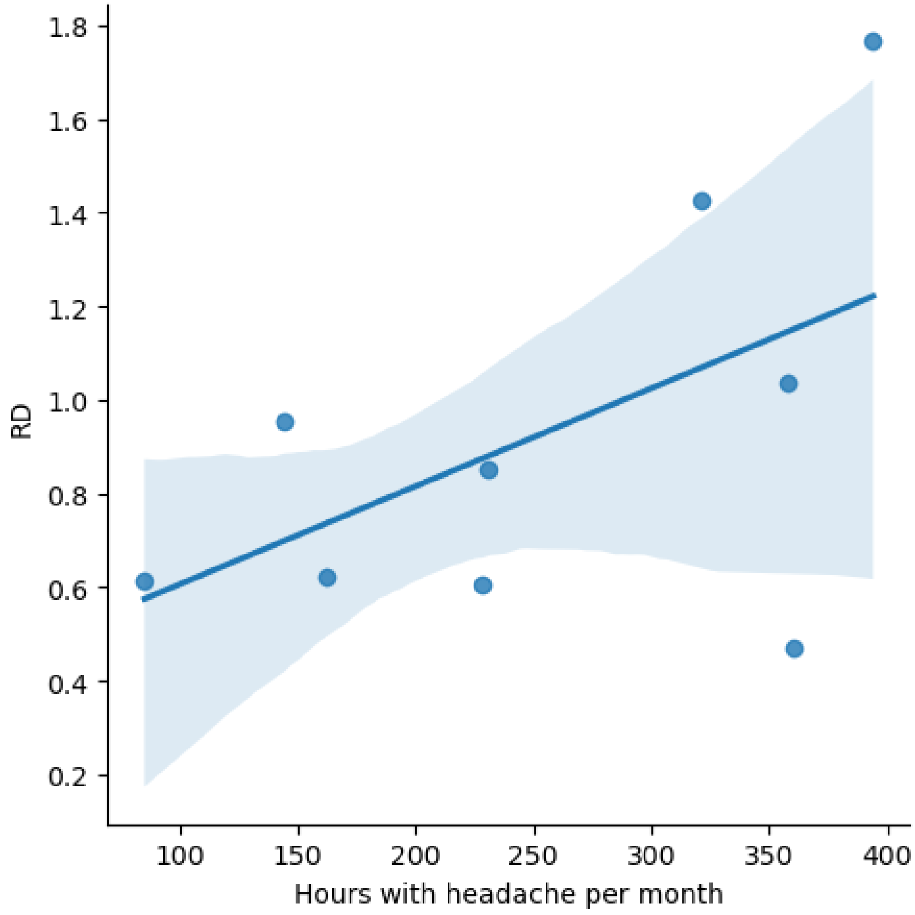

Positive Cluster: 4

Name	Left-Cerebellum-Cortex
CWP	0.02817
Size	94 Voxel
TalX, TalY, TalZ	-7.6, -64.32, -33.8
**Linear Regression**	
Slope	0.00073
Intercept	0.79280
R-value	0.30415
P-Value	0.42620
**Spearman Regression**	
Spearman Correlation	0.23333
P-Value	0.54570

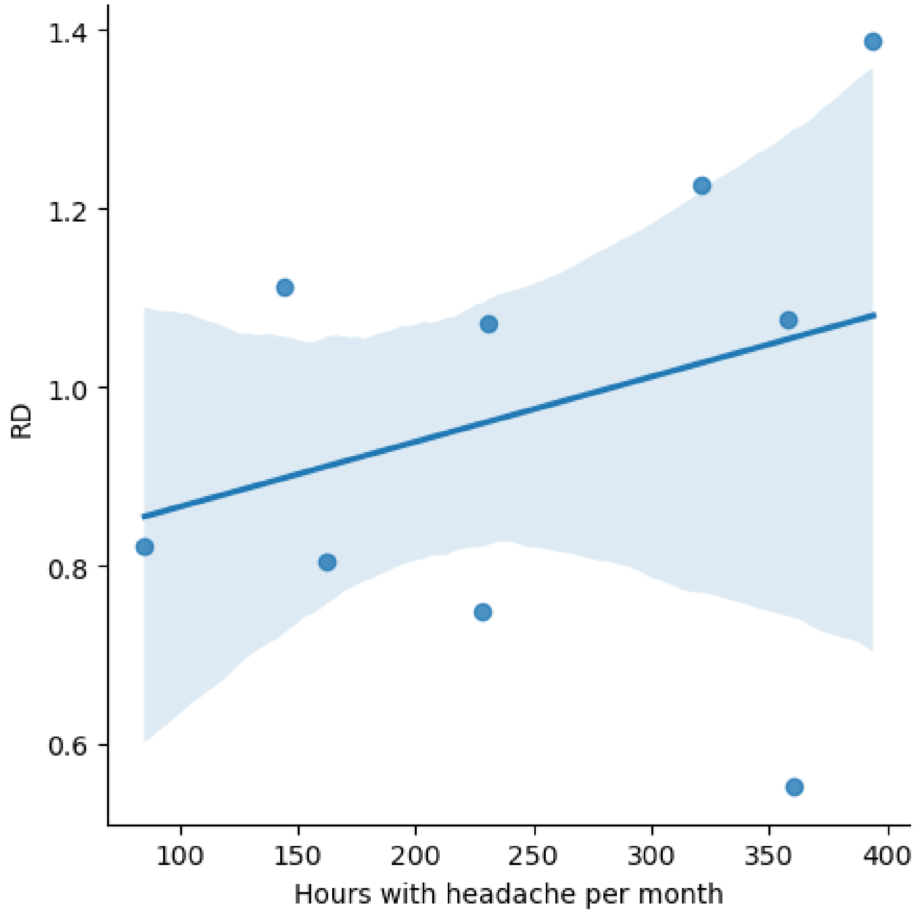

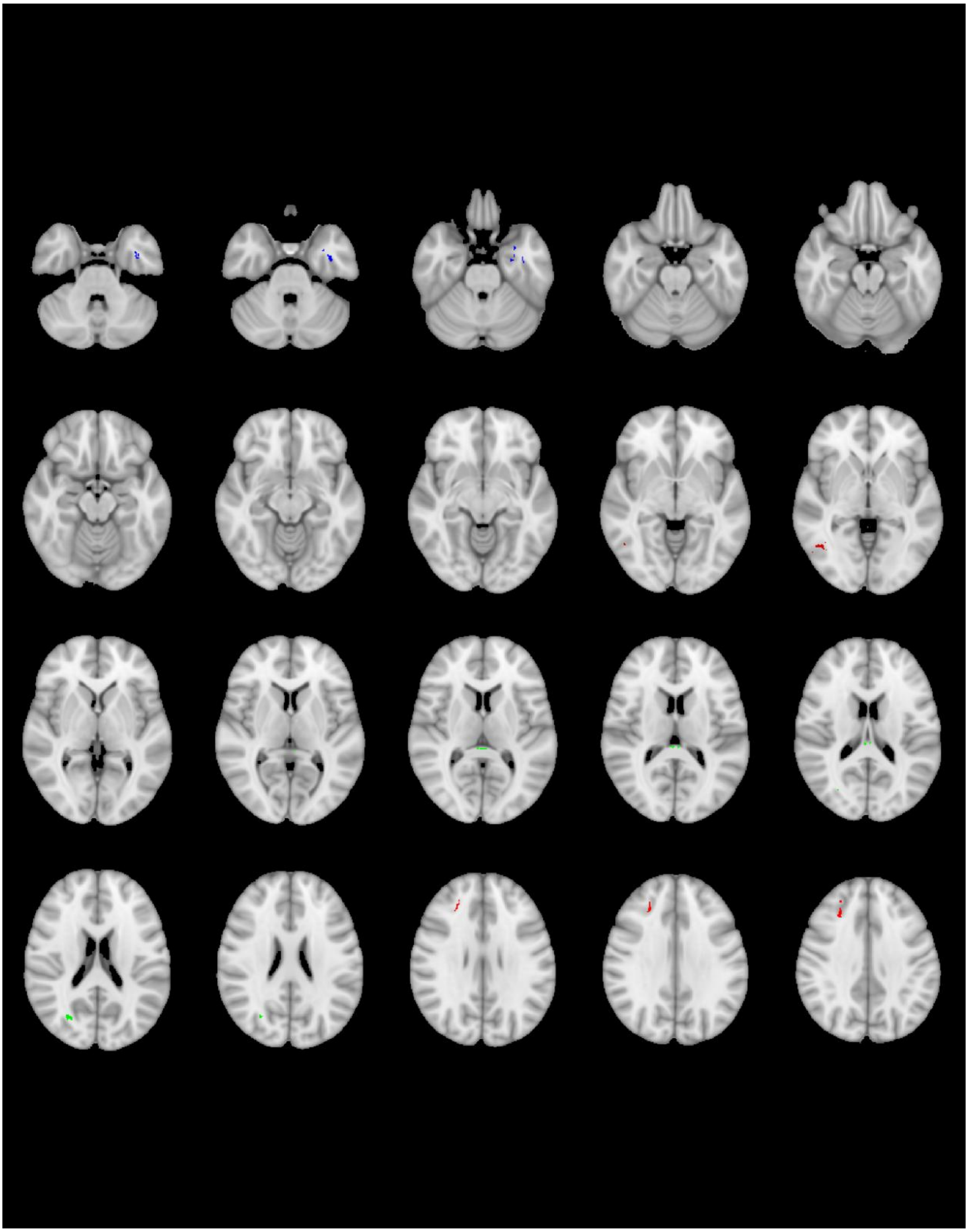

Figure 2 Shown is the negative decadic logarithm of the clusterwise p-values. The threshold for the clusters is a clusterwise p-value < 0.05 (>1.3 for the negative decadic logarithm).
Red marked spots indicate clusters with a negative correlation of the FA with the hours with headache per month.
Green marked spots indicate clusters with a negative correlation of the AD with the hours with headache per month.
Blue marked spots indicate clusters with a negative correlation of the RD with the hours with headache per month.

FA

**Negative Cluster: 1**	
Name	Right-Cerebral-White-Matter
CWP	0.00025
Size	219 Voxel
TalX, TalY, TalZ	26.43, 27.86, 30.4
**Linear Regression**	
Slope	-0.00044
Intercept	0.38002
R-value	-0.39498
P-Value	0.29276
**Spearman Regression**	
Spearman Correlation	-0.35000
P-Value	0.35582

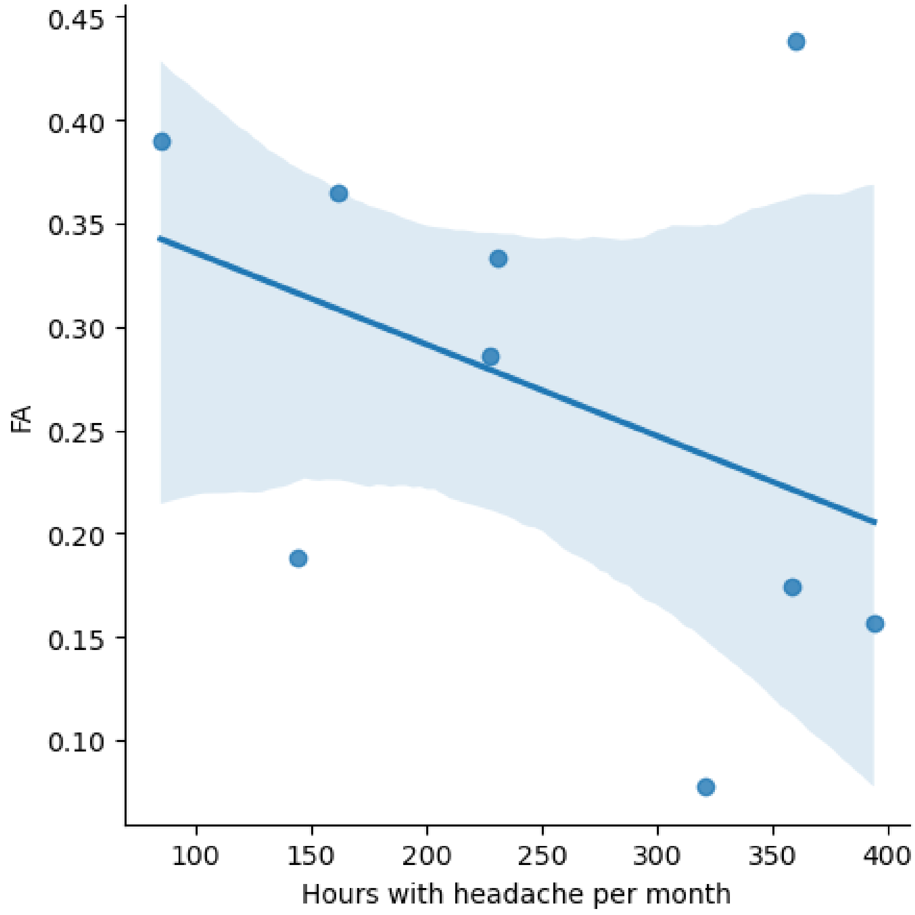

Negative Cluster: 2

Name	ctx-rh-inferiortemporal
CWP	0.03783
Size	143 Voxel
TalX, TalY, TalZ	48.32, -59.52, -1.34
**Linear Regression**	
Slope	-0.00022
Intercept	0.27015
R-value	-0.28673
P-Value	0.45443
**Spearman Regression**	
Spearman Correlation	-0.26667
P-Value	0.48792

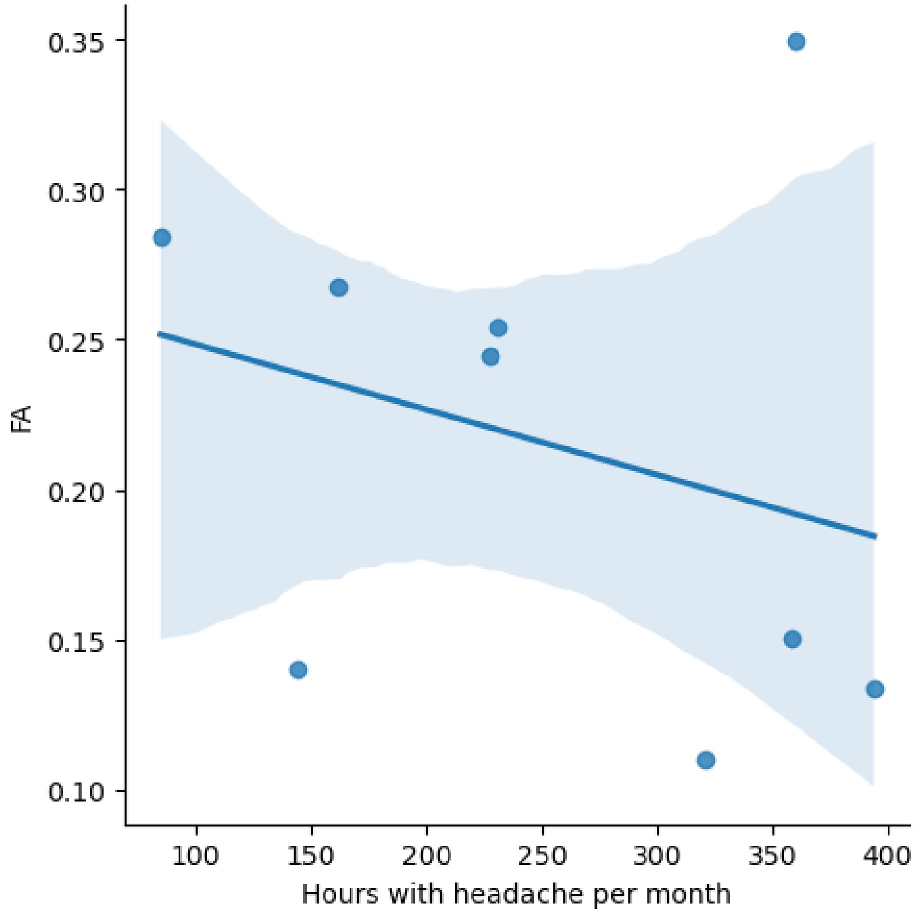

AD

**Negative Cluster: 1**	
Name	CC_Posterior
CWP	0.01008
Size	112 Voxel
TalX, TalY, TalZ	2.55, -29.92, 14.02
**Linear Regression**	
Slope	-0.00175
Intercept	1.65704
R-value	-0.45411
P-Value	0.21949
**Spearman Regression**	
Spearman Correlation	-0.41667
P-Value	0.26459

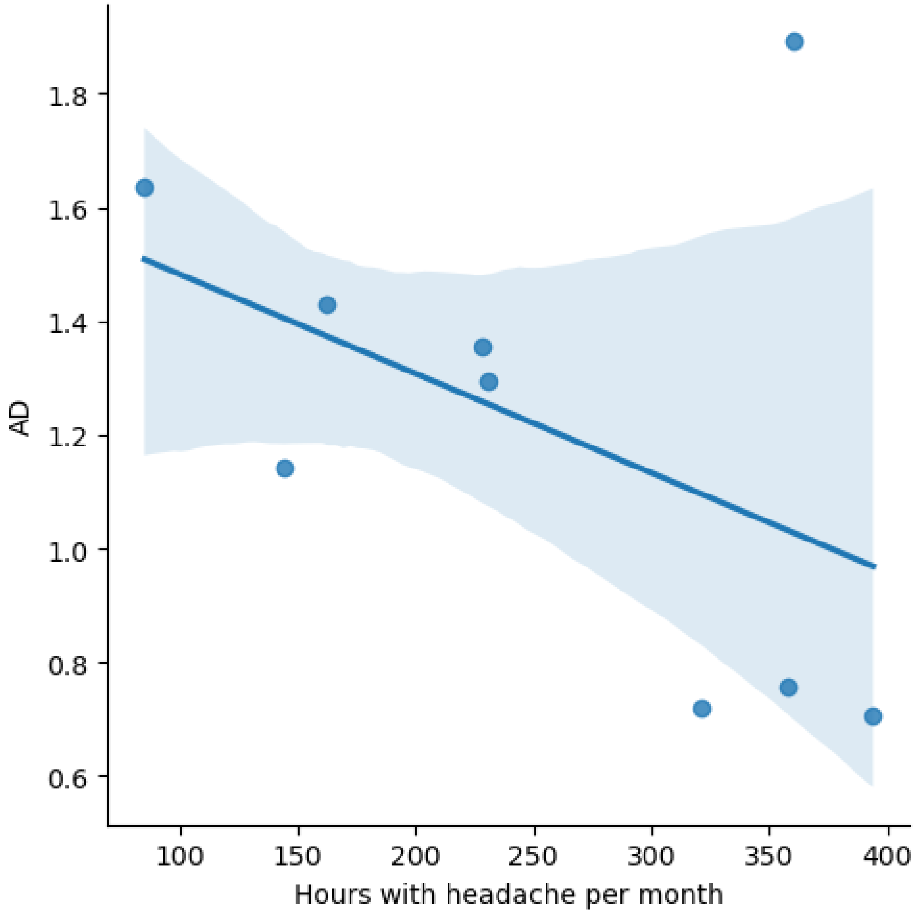

Negative Cluster: 2

Name	Right-Cerebral-White-Matter
CWP	0.01292
Size	109 Voxel
TalX, TalY, TalZ	25.0, -68.71, 20.51
**Linear Regression**	
Slope	-0.00057
Intercept	1.40451
R-value	-0.55597
P-Value	0.12009
**Spearman Regression**	
Spearman Correlation	-0.36667
P-Value	0.33174

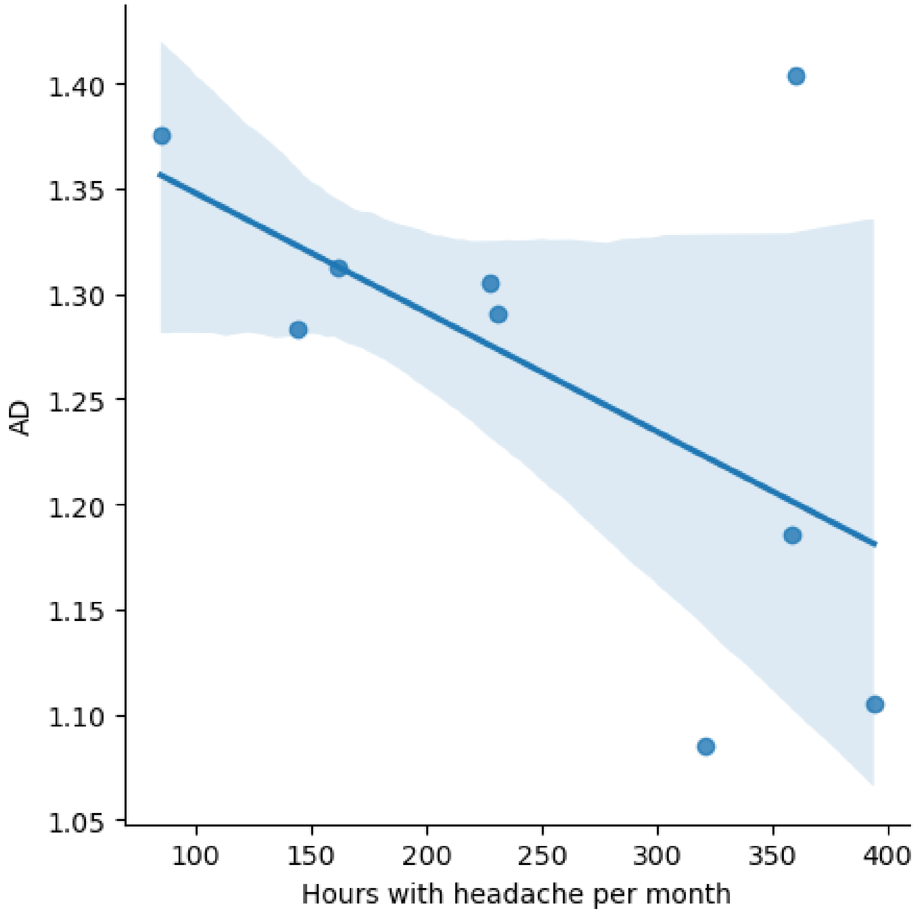

RD

**Negative Cluster: 1**	
Name	Left-Amygdala
CWP	0.00100
Size	126 Voxel
TalX, TalY, TalZ	-28.29, -6.2, -22.97
**Linear Regression**	
Slope	0.00056
Intercept	0.60958
R-value	0.26539
P-Value	0.49009
**Spearman Regression**	
Spearman Correlation	0.43333
P-Value	0.24395

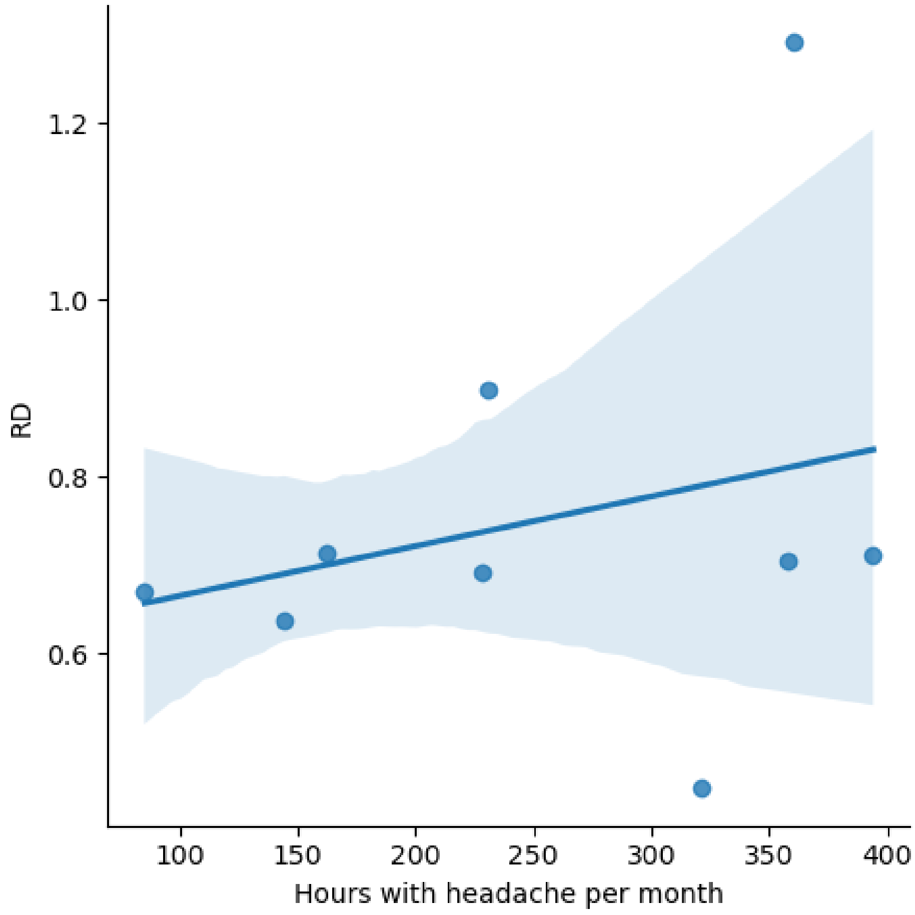

Negative Cluster: 2

Name	Left-Cerebral-White-Matter
CWP	0.00408
Size	111 Voxel
TalX, TalY, TalZ	-34.17, -5.33, -26.45
**Linear Regression**	
Slope	-0.00027
Intercept	0.68660
R-value	-0.22729
P-Value	0.55644
**Spearman Regression**	
Spearman Correlation	-0.30000
P-Value	0.43285

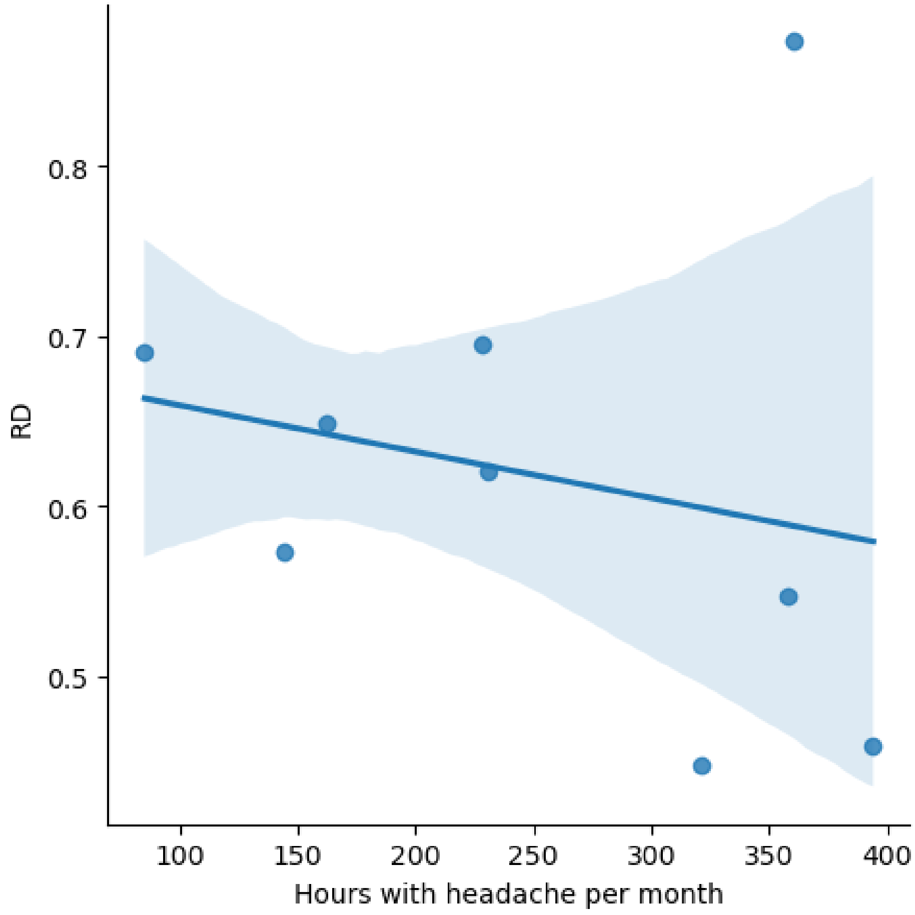

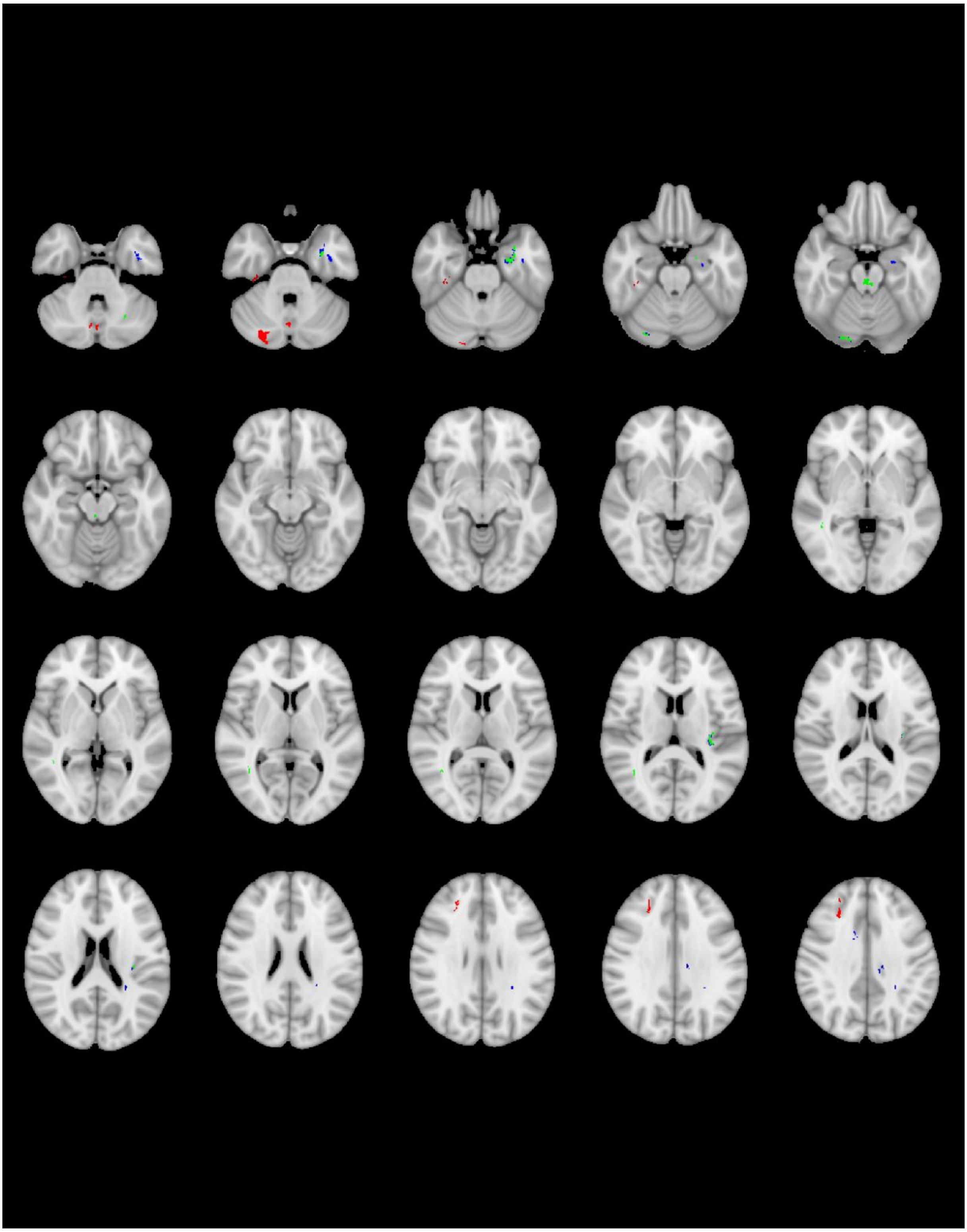

Figure 3 Shown is the negative decadic logarithm of the clusterwise p-values. The threshold for the clusters is a clusterwise p-value < 0.05 (>1.3 for the negative decadic logarithm).
Red marked spots indicate clusters with a positive correlation of the FA with the sum of NRS per hour per month
Green marked spots indicate clusters with a positive correlation of the AD with the sum of NRS per hour per month
Blue marked spots indicate clusters with a positive correlation of the RD with the sum of NRS per hour per month.

FA

**Positive Cluster: 1**	
Name	Right-Cerebellum-Cortex
CWP	0.00008
Size	265 Voxel
TalX, TalY, TalZ	2.13, -66.91, -25.15
**Linear Regression**	
Slope	0.00009
Intercept	0.08722
R-value	0.61812
P-Value	0.07604
**Spearman Regression**	
Spearman Correlation	0.10000
P-Value	0.79797

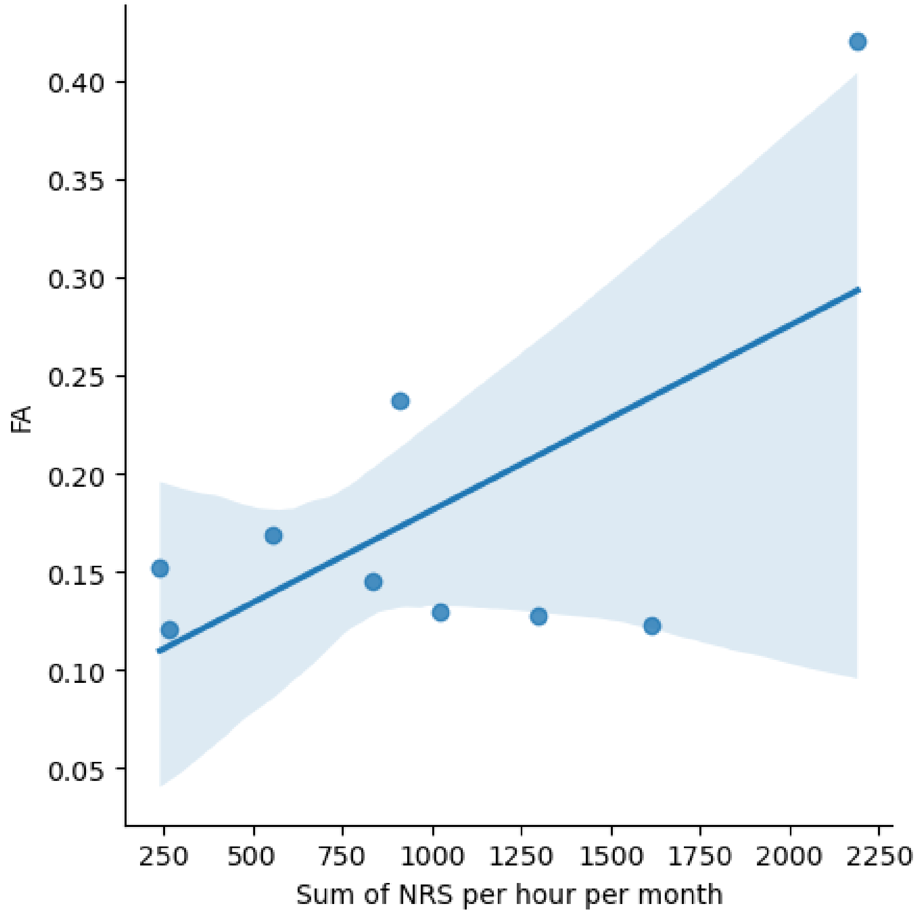

Positive Cluster: 2

Name	Right-Cerebral-White-Matter
CWP	0.00033
Size	218 Voxel
TalX, TalY, TalZ	26.43, 27.86, 30.4
**Linear Regression**	
Slope	0.00004
Intercept	0.21587
R-value	0.23646
P-Value	0.54018
**Spearman Regression**	
Spearman Correlation	0.16667
P-Value	0.66823

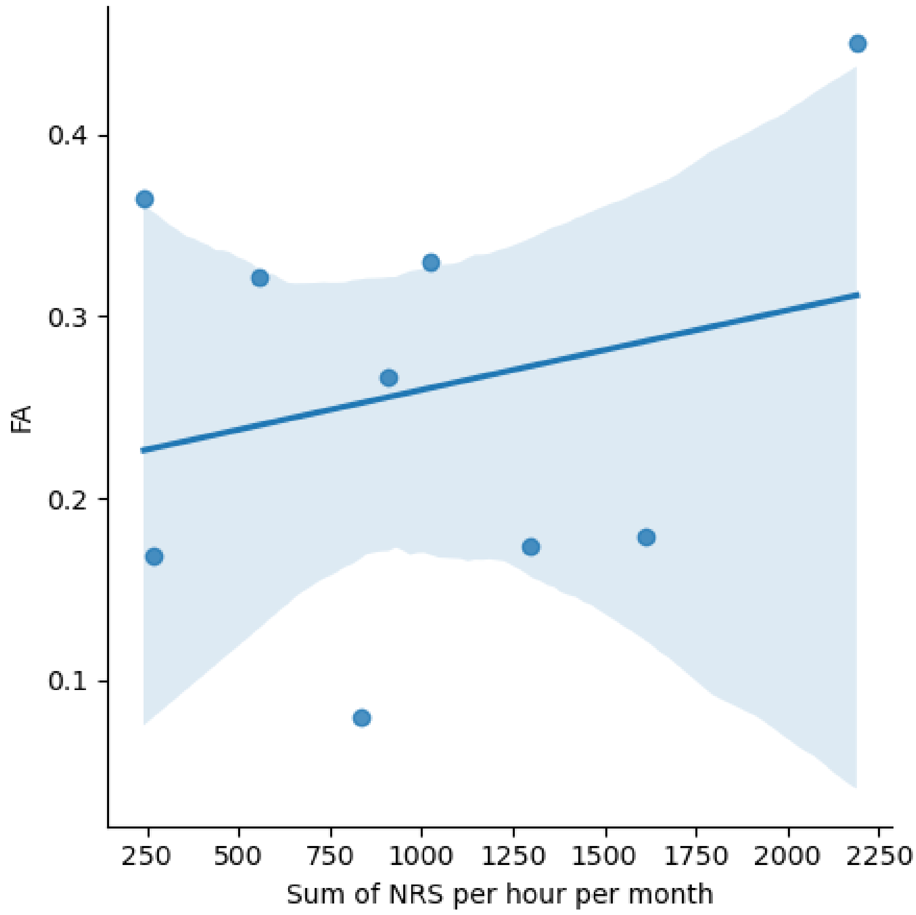

Positive Cluster: 3

Name	Right-Cerebellum-Cortex
CWP	0.00050
Size	210 Voxel
TalX, TalY, TalZ	16.77, -87.23, -19.92
**Linear Regression**	
Slope	0.00008
Intercept	0.13848
R-value	0.56850
P-Value	0.11022
**Spearman Regression**	
Spearman Correlation	0.23333
P-Value	0.54570

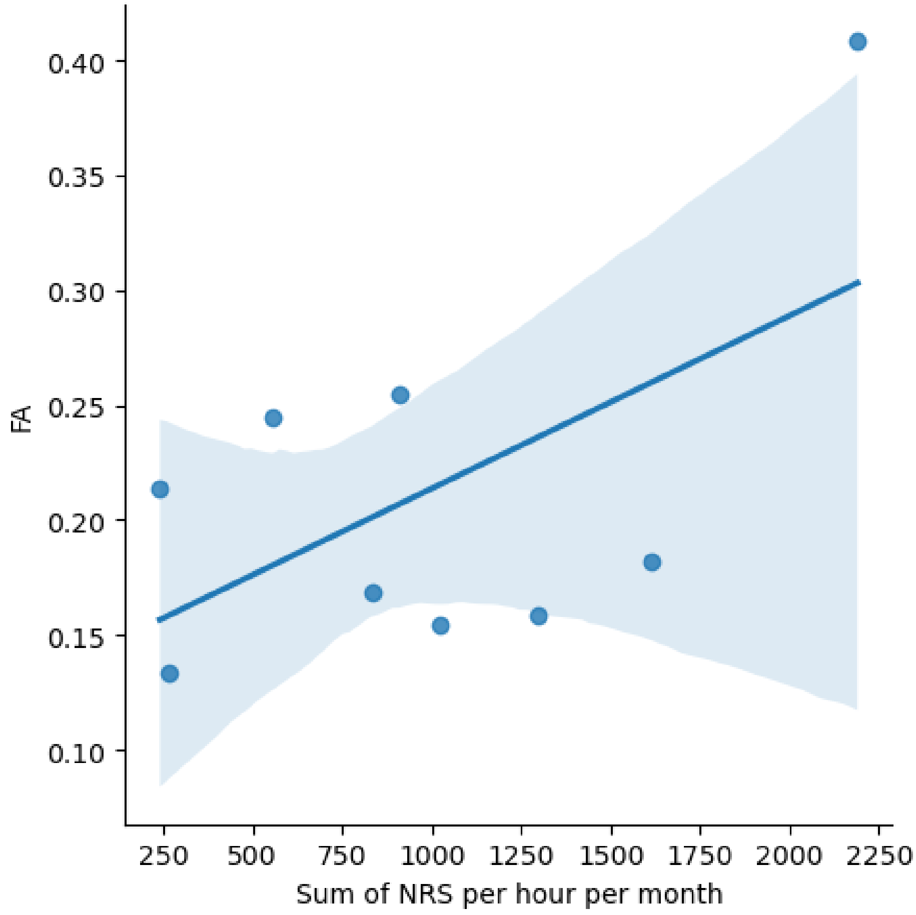

Positive Cluster: 4

Name	Unknown
CWP	0.04767
Size	139 Voxel
TalX, TalY, TalZ	35.12, -30.53, -25.47
**Linear Regression**	
Slope	0.00011
Intercept	0.07017
R-value	0.68333
P-Value	0.04244
**Spearman Regression**	
Spearman Correlation	0.20000
P-Value	0.60590

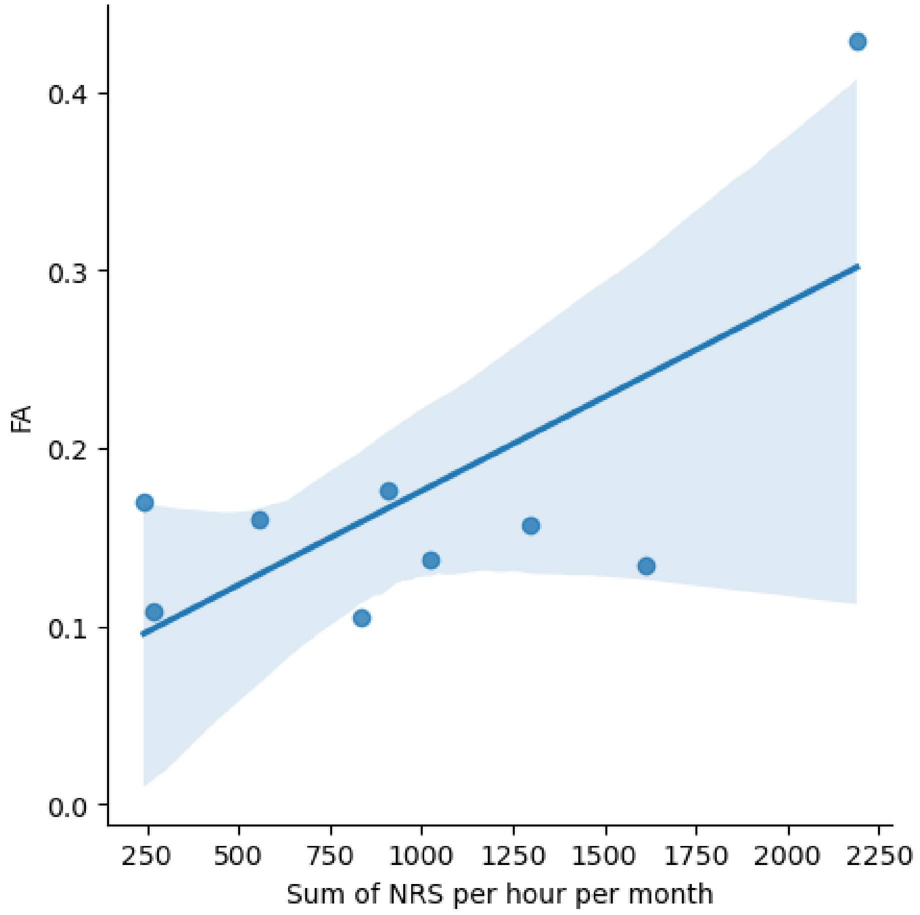

AD

**Positive Cluster: 1**	
Name	Left-Hippocampus
CWP	0.00008
Size	282 Voxel
TalX, TalY, TalZ	-18.43, -10.12, -21.01
**Linear Regression**	
Slope	0.00025
Intercept	0.78470
R-value	0.77149
P-Value	0.01490
**Spearman Regression**	
Spearman Correlation	0.80000
P-Value	0.00963

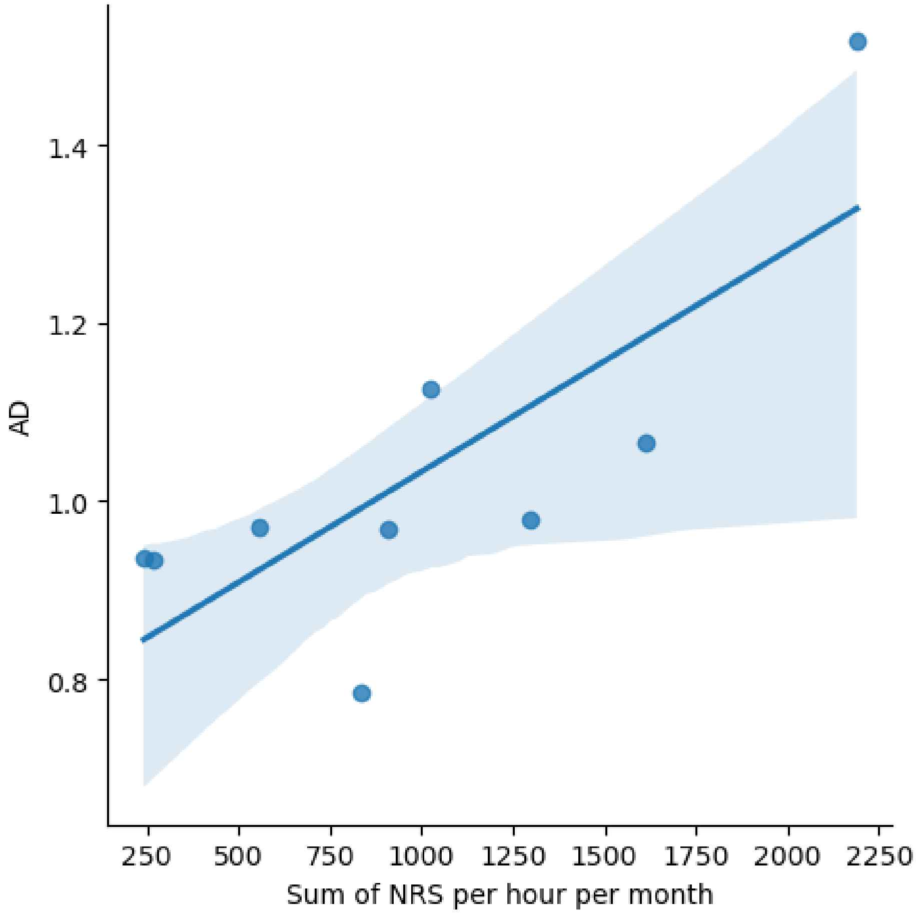

Positive Cluster: 2

Name	Right-Cerebral-White-Matter
CWP	0.00333
Size	125 Voxel
TalX, TalY, TalZ	39.29, -51.24, 6.11
**Linear Regression**	
Slope	0.00004
Intercept	1.16293
R-value	0.31834
P-Value	0.40378
**Spearman Regression**	
Spearman Correlation	0.15000
P-Value	0.70009

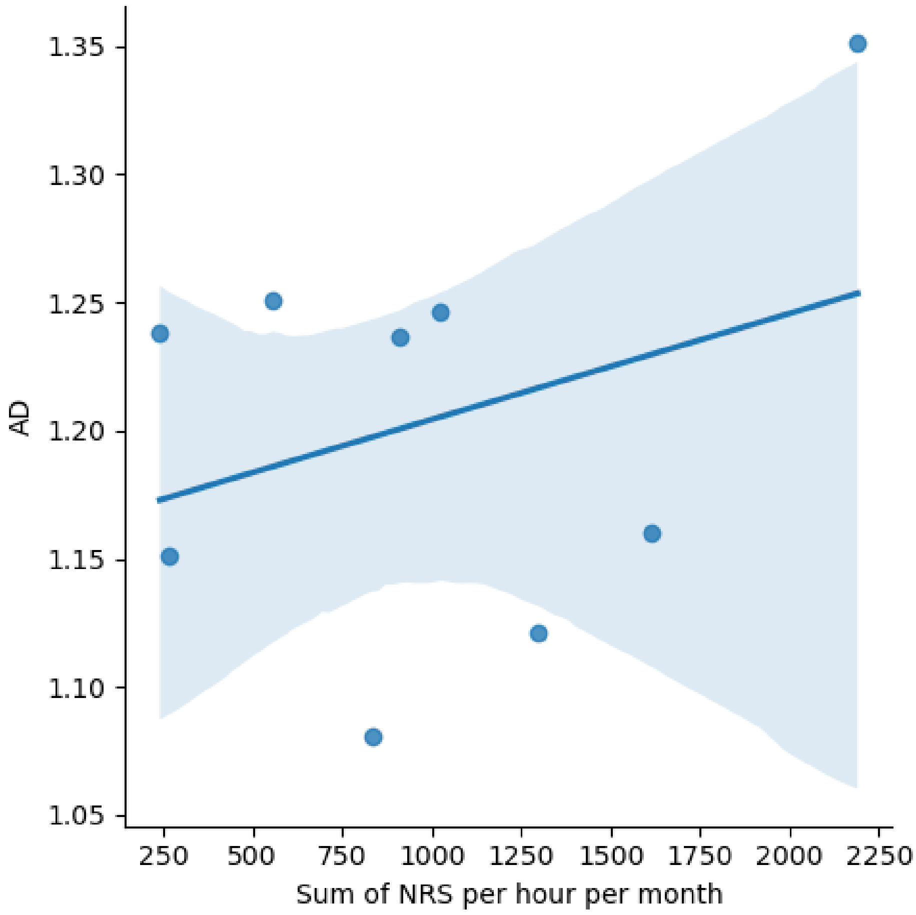

Positive Cluster: 3

Name	Left-Cerebellum-White-Matter
CWP	0.00833
Size	117 Voxel
TalX, TalY, TalZ	-23.6, -64.76, -28.05
**Linear Regression**	
Slope	0.00021
Intercept	0.67963
R-value	0.79929
P-Value	0.00974
**Spearman Regression**	
Spearman Correlation	0.71667
P-Value	0.02982

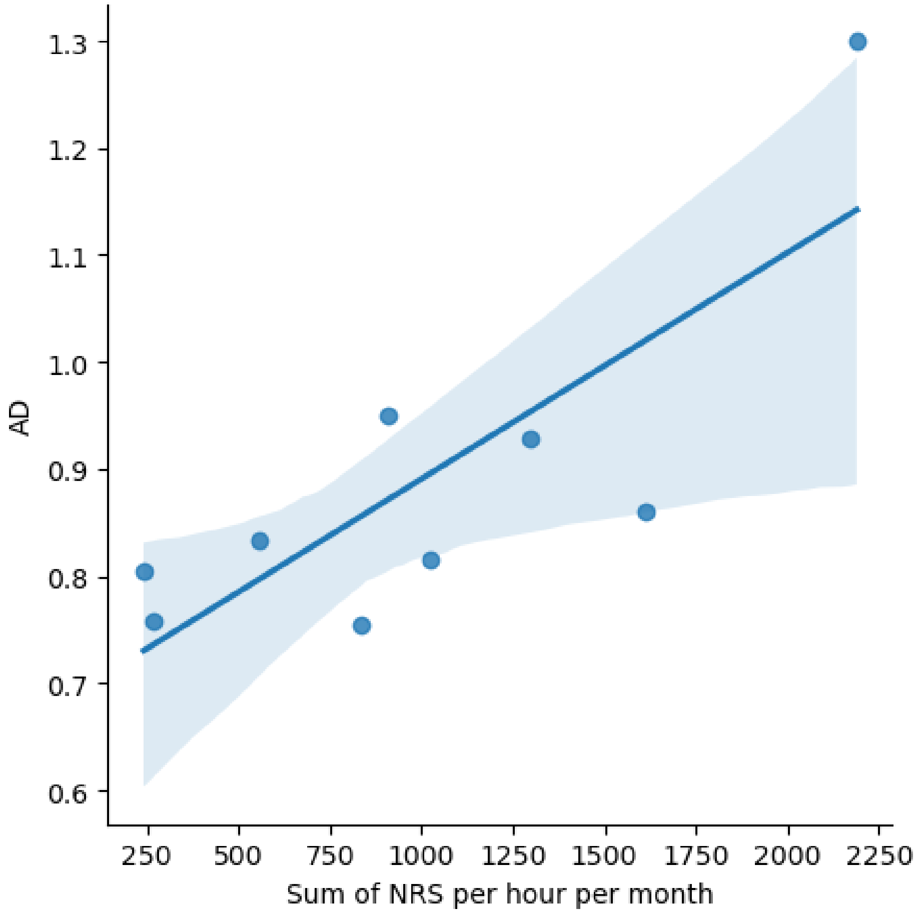

Positive Cluster: 4

Name	Right-Cerebellum-Cortex
CWP	0.01417
Size	110 Voxel
TalX, TalY, TalZ	24.65, -80.29, -15.06
**Linear Regression**	
Slope	0.00025
Intercept	0.83402
R-value	0.71996
P-Value	0.02872
**Spearman Regression**	
Spearman Correlation	0.51667
P-Value	0.15439

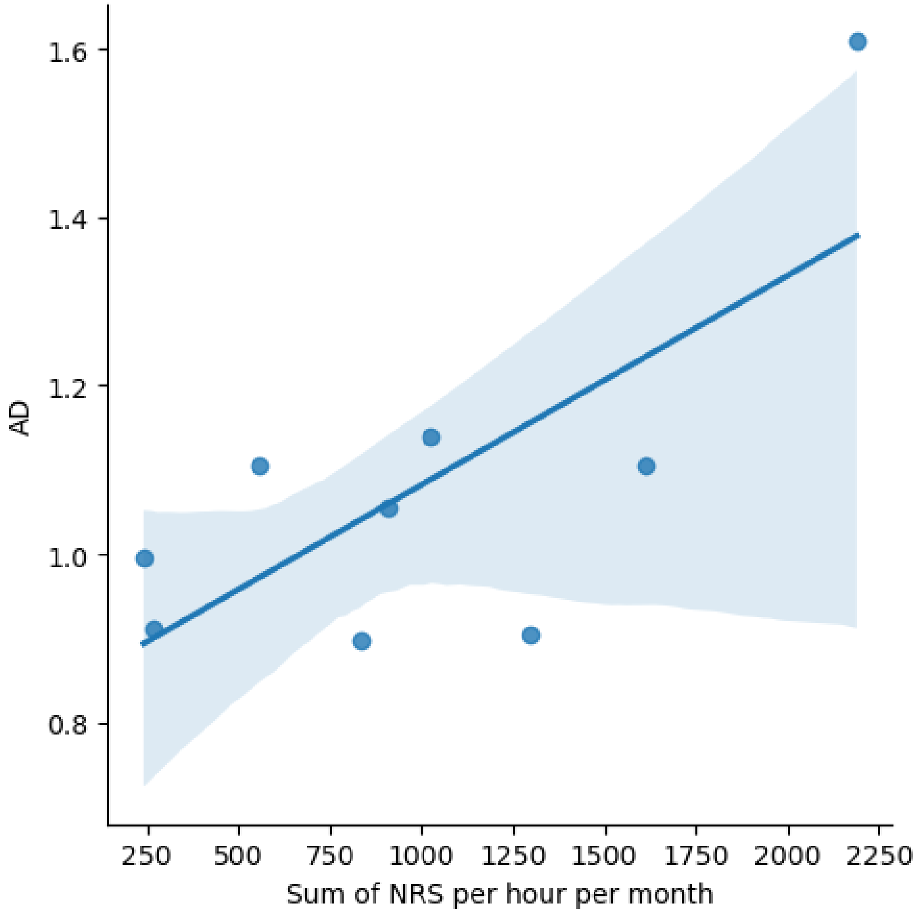

Positive Cluster: 5

Name	ctx-lh-insula
CWP	0.01517
Size	109 Voxel
TalX, TalY, TalZ	-32.06, -21.84, 10.5
**Linear Regression**	
Slope	0.00017
Intercept	0.82893
R-value	0.72868
P-Value	0.02596
**Spearman Regression**	
Spearman Correlation	0.56667
P-Value	0.11163

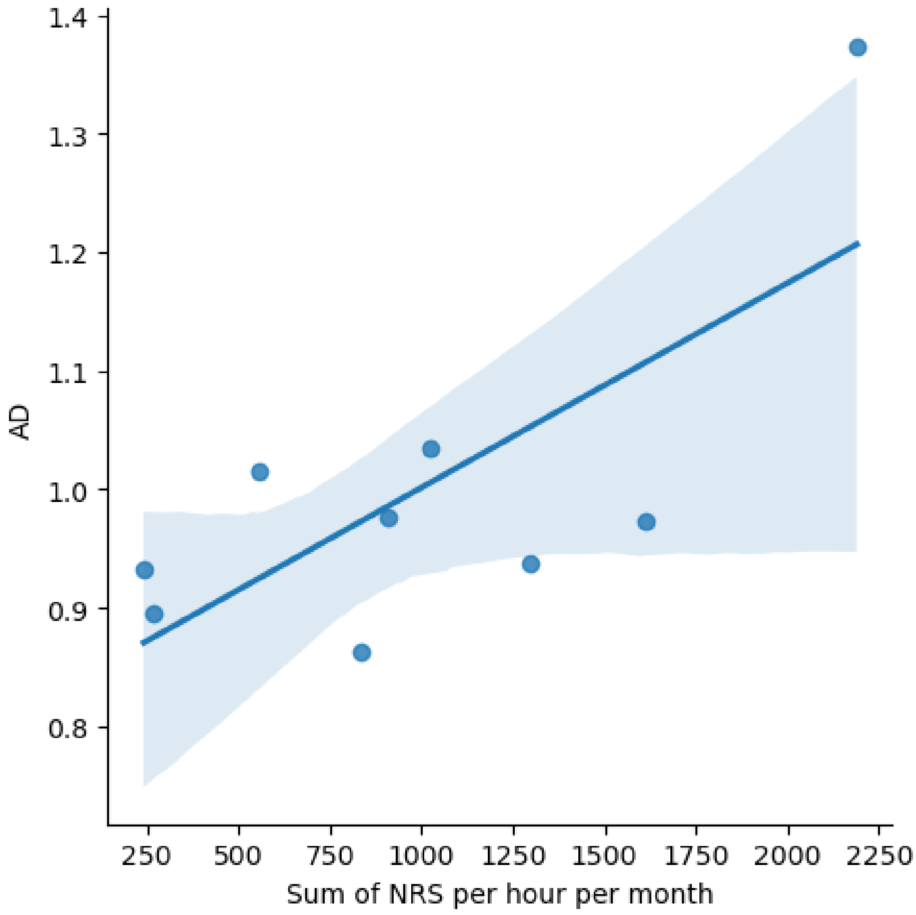

Positive Cluster: 6

Name	Brain-Stem
CWP	0.01517
Size	109 Voxel
TalX, TalY, TalZ	2.18, -30.55, -16.56
**Linear Regression**	
Slope	0.00040
Intercept	0.96490
R-value	0.80277
P-Value	0.00920
**Spearman Regression**	
Spearman Correlation	0.85000
P-Value	0.00370

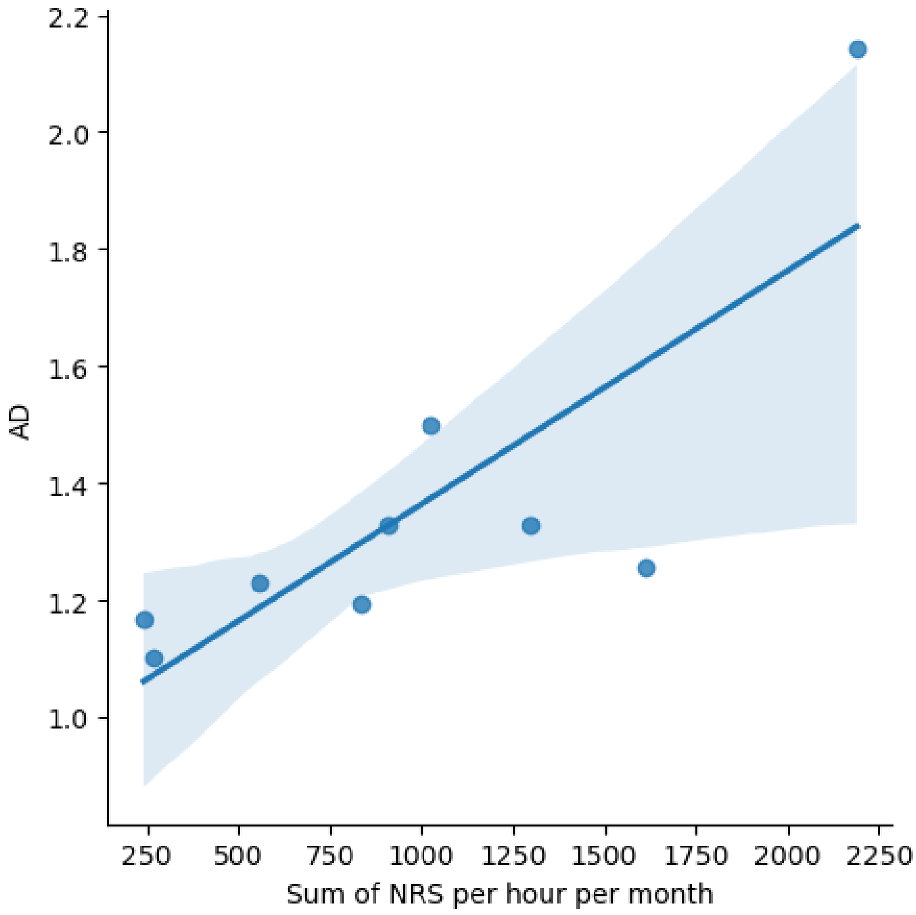

Positive Cluster: 7

Name	Right-Cerebral-White-Matter
CWP	0.03700
Size	99 Voxel
TalX, TalY, TalZ	20.19, 1.08, 36.05
**Linear Regression**	
Slope	0.00003
Intercept	0.97892
R-value	0.30602
P-Value	0.42321
**Spearman Regression**	
Spearman Correlation	0.30000
P-Value	0.43285

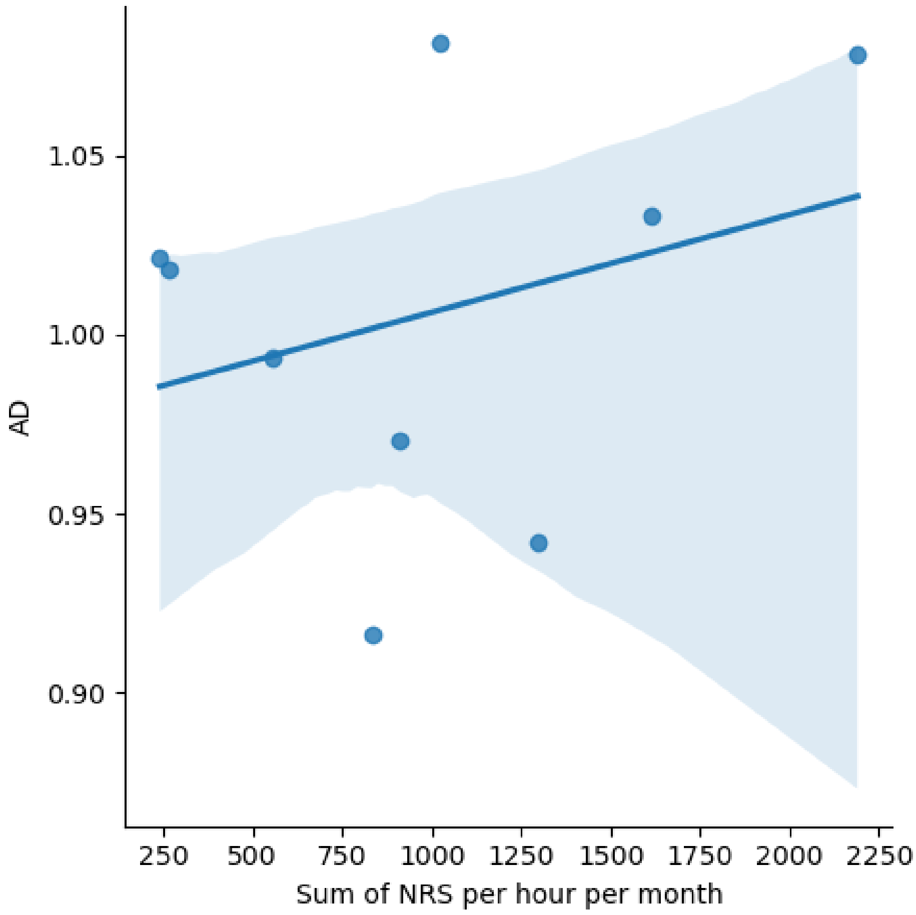

RD

**Positive Cluster: 1**	
Name	Left-Amygdala
CWP	0.00008
Size	438 Voxel
TalX, TalY, TalZ	-28.29, -6.2, -22.97
**Linear Regression**	
Slope	0.00023
Intercept	0.55708
R-value	0.75250
P-Value	0.01930
**Spearman Regression**	
Spearman Correlation	0.80000
P-Value	0.00963

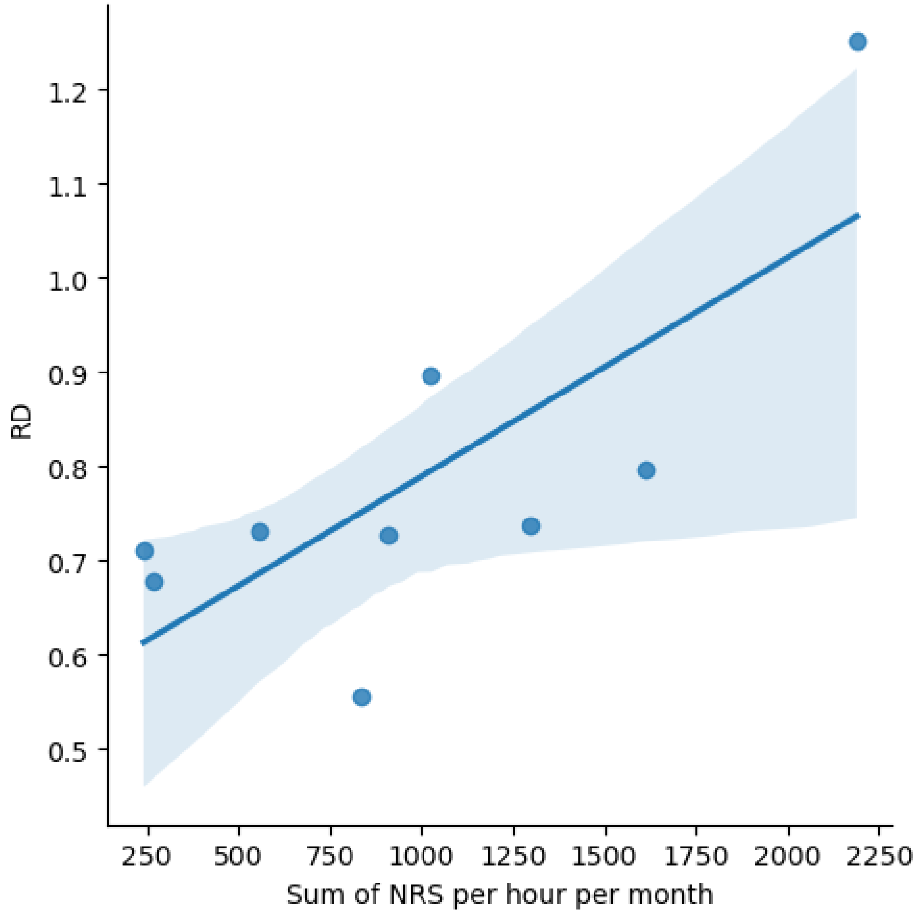

Positive Cluster: 2

Name	Left-Cerebral-White-Matter
CWP	0.00008
Size	146 Voxel
TalX, TalY, TalZ	-34.17, -5.33, -26.45
**Linear Regression**	
Slope	0.00014
Intercept	0.52302
R-value	0.52152
P-Value	0.14988
**Spearman Regression**	
Spearman Correlation	0.05000
P-Value	0.89835

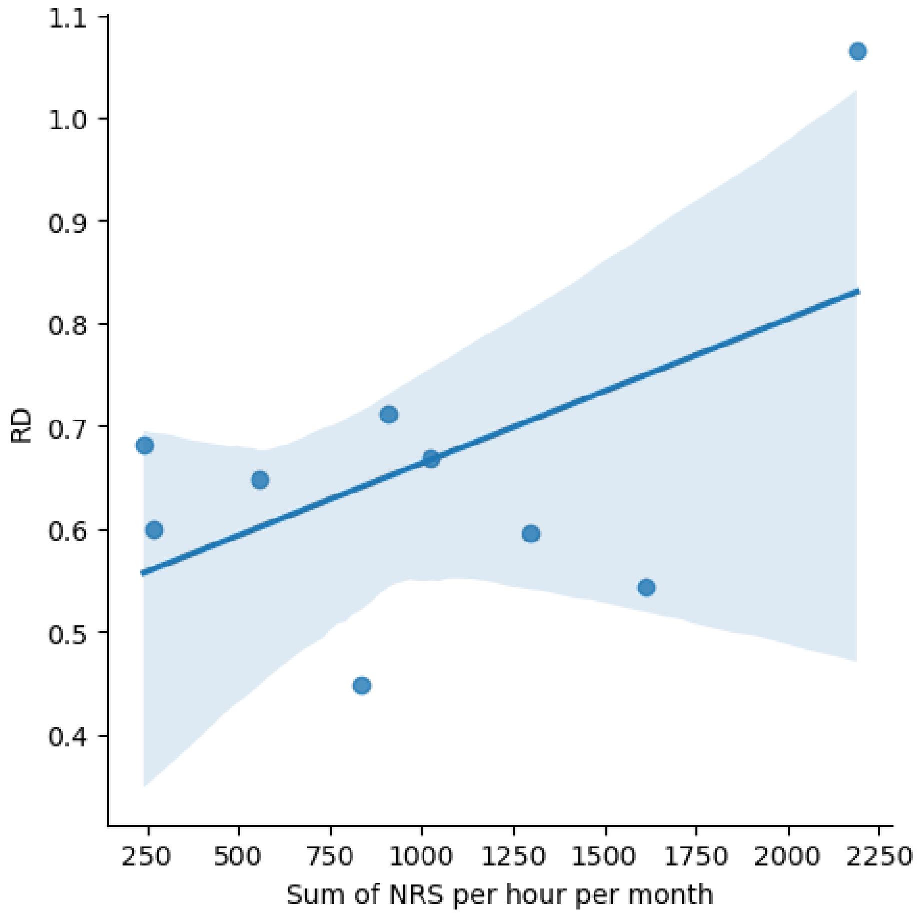

Positive Cluster: 3

Name	ctx-lh-insula
CWP	0.00017
Size	135 Voxel
TalX, TalY, TalZ	-33.18, -22.46, 16.98
**Linear Regression**	
Slope	0.00017
Intercept	0.54174
R-value	0.79344
P-Value	0.01071
**Spearman Regression**	
Spearman Correlation	0.76667
P-Value	0.01594

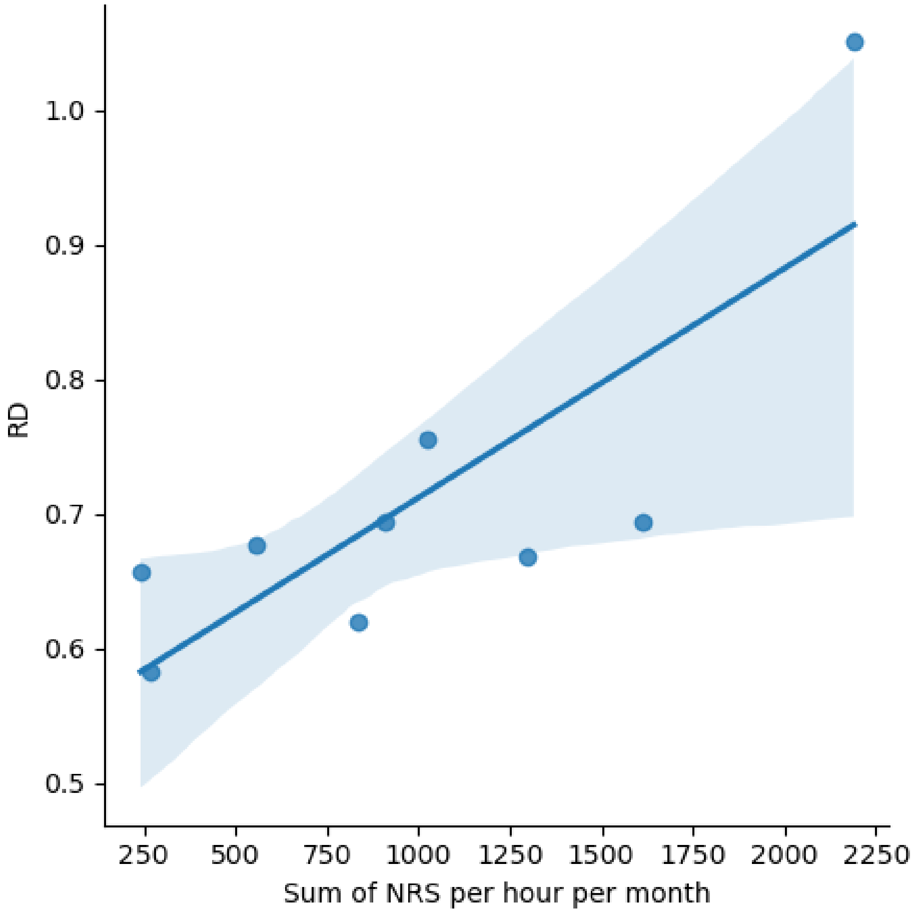

Positive Cluster: 4

Name	Right-Cerebral-White-Matter
CWP	0.00367
Size	114 Voxel
TalX, TalY, TalZ	12.35, 3.89, 32.14
**Linear Regression**	
Slope	0.00009
Intercept	0.52500
R-value	0.57809
P-Value	0.10301
**Spearman Regression**	
Spearman Correlation	0.26667
P-Value	0.48792

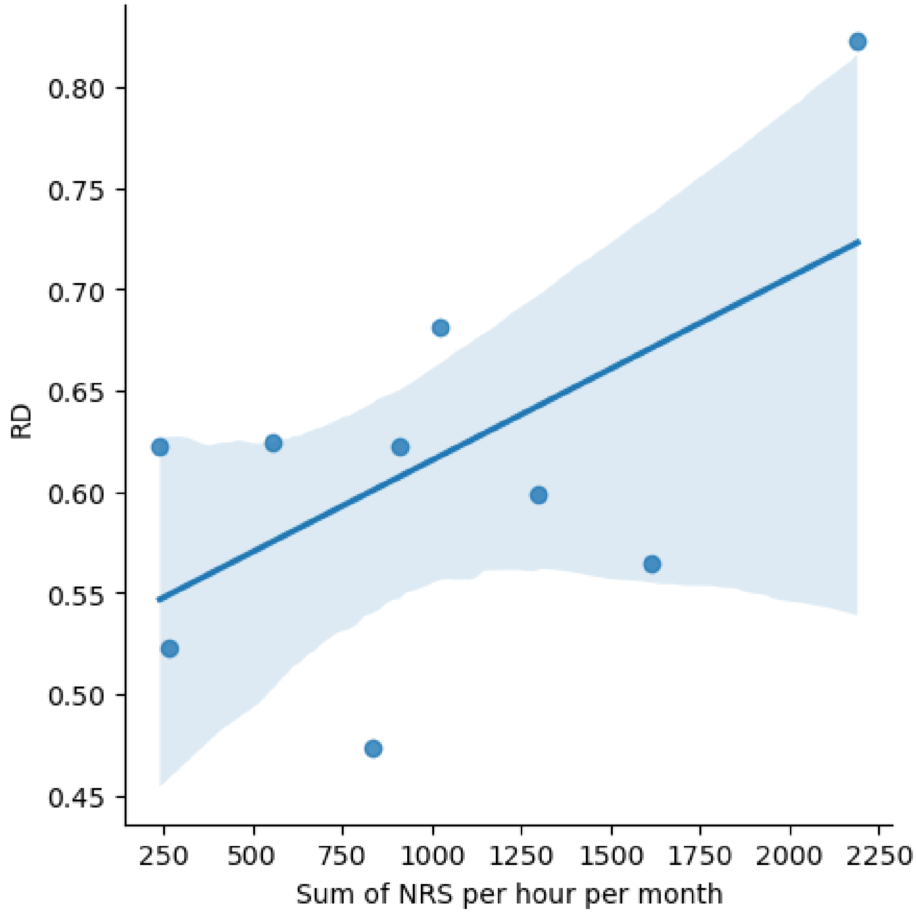

Positive Cluster: 5

Name	Left-Cerebral-White-Matter
CWP	0.00717
Size	107 Voxel
TalX, TalY, TalZ	-12.72, 0.02, 44.0
**Linear Regression**	
Slope	0.00019
Intercept	0.50309
R-value	0.56415
P-Value	0.11359
**Spearman Regression**	
Spearman Correlation	0.30000
P-Value	0.43285

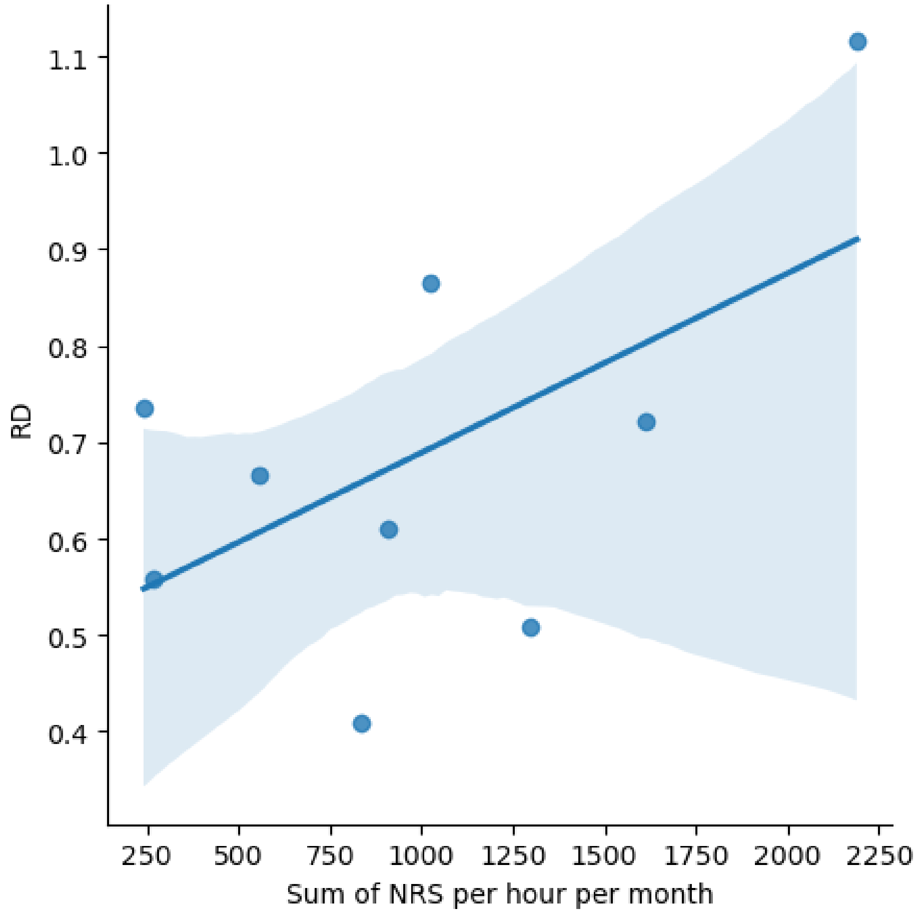

Positive Cluster: 6

Name	Left-Cerebral-White-Matter
CWP	0.03817
Size	91 Voxel
TalX, TalY, TalZ	-26.39, -40.9, 18.74
**Linear Regression**	
Slope	0.00013
Intercept	0.40738
R-value	0.80235
P-Value	0.00926
**Spearman Regression**	
Spearman Correlation	0.73333
P-Value	0.02455

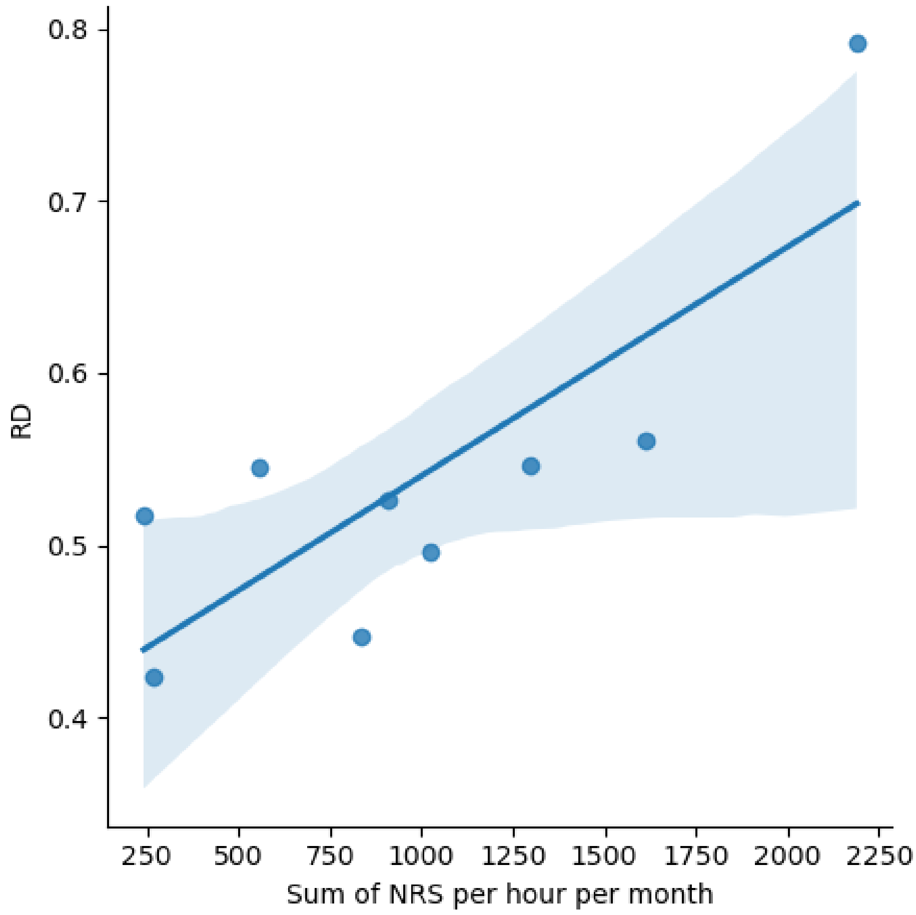

Positive Cluster: 7

Name	Right-Cerebellum-Cortex
CWP	0.04225
Size	90 Voxel
TalX, TalY, TalZ	24.65, -80.29, -15.06
**Linear Regression**	
Slope	0.00025
Intercept	0.60692
R-value	0.71877
P-Value	0.02912
**Spearman Regression**	
Spearman Correlation	0.46667
P-Value	0.20539

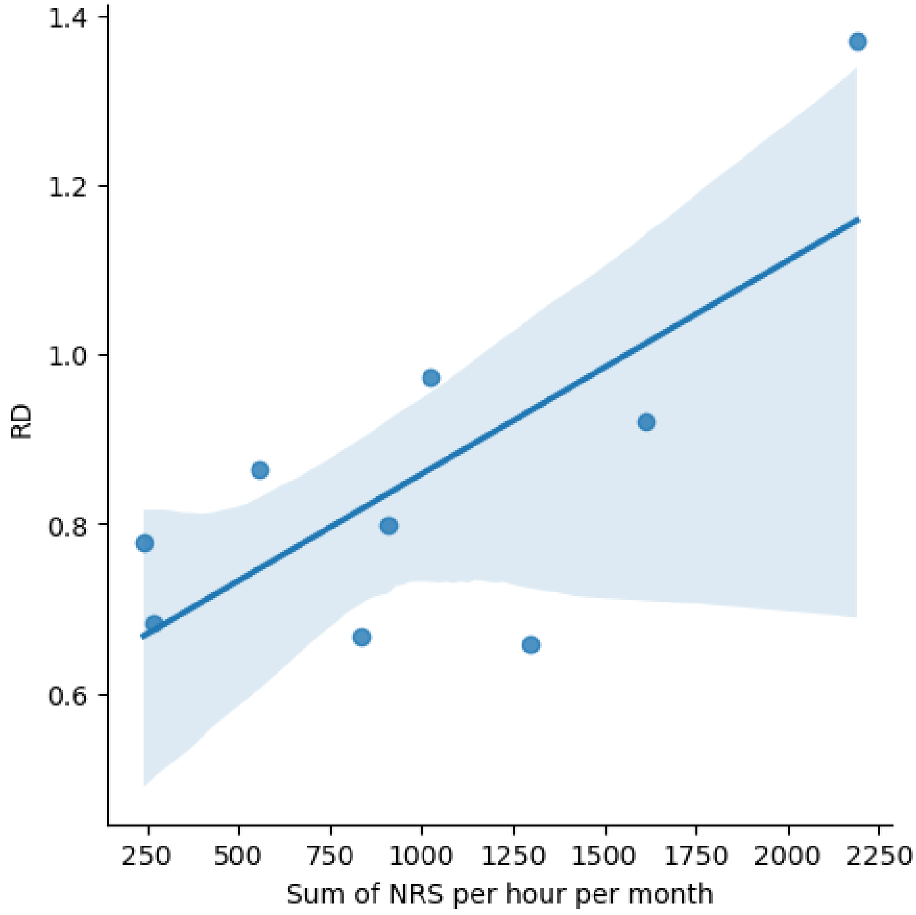

**Positive Cluster: 8**	
Name	Left-Cerebral-White-Matter
CWP	0.04967
Size	88 Voxel
TalX, TalY, TalZ	-12.58, -20.12, 30.06
**Linear Regression**	
Slope	0.00008
Intercept	0.36654
R-value	0.74349
P-Value	0.02167
**Spearman Regression**	
Spearman Correlation	0.63333
P-Value	0.06709

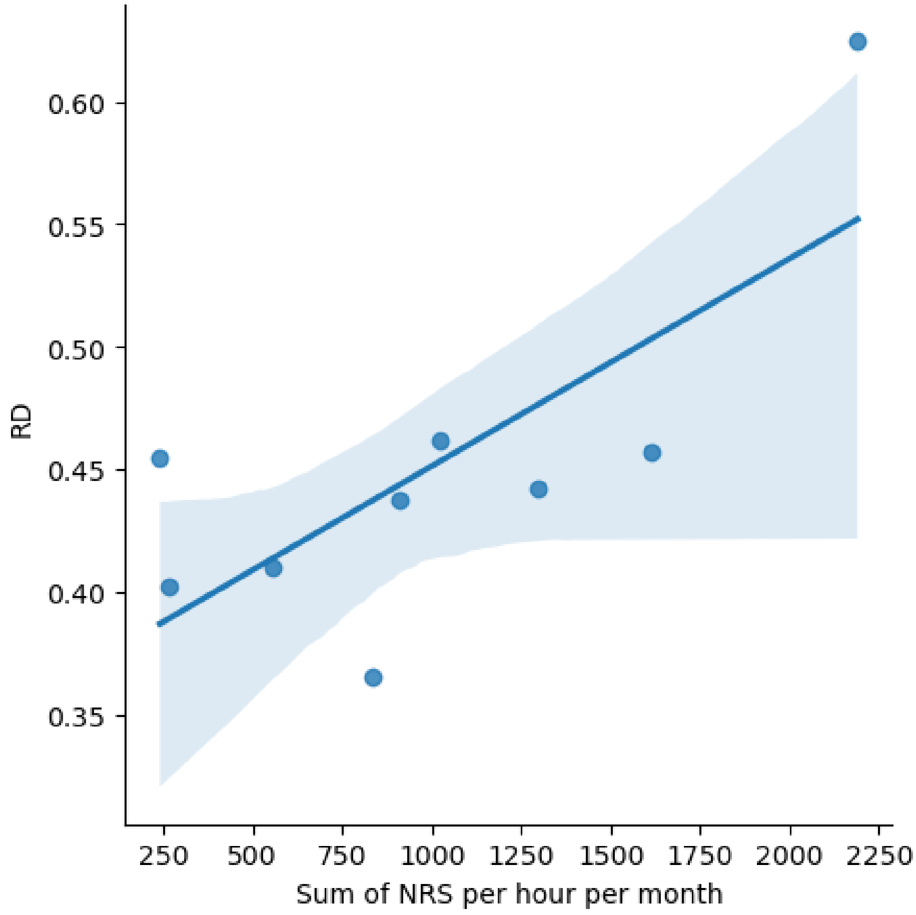

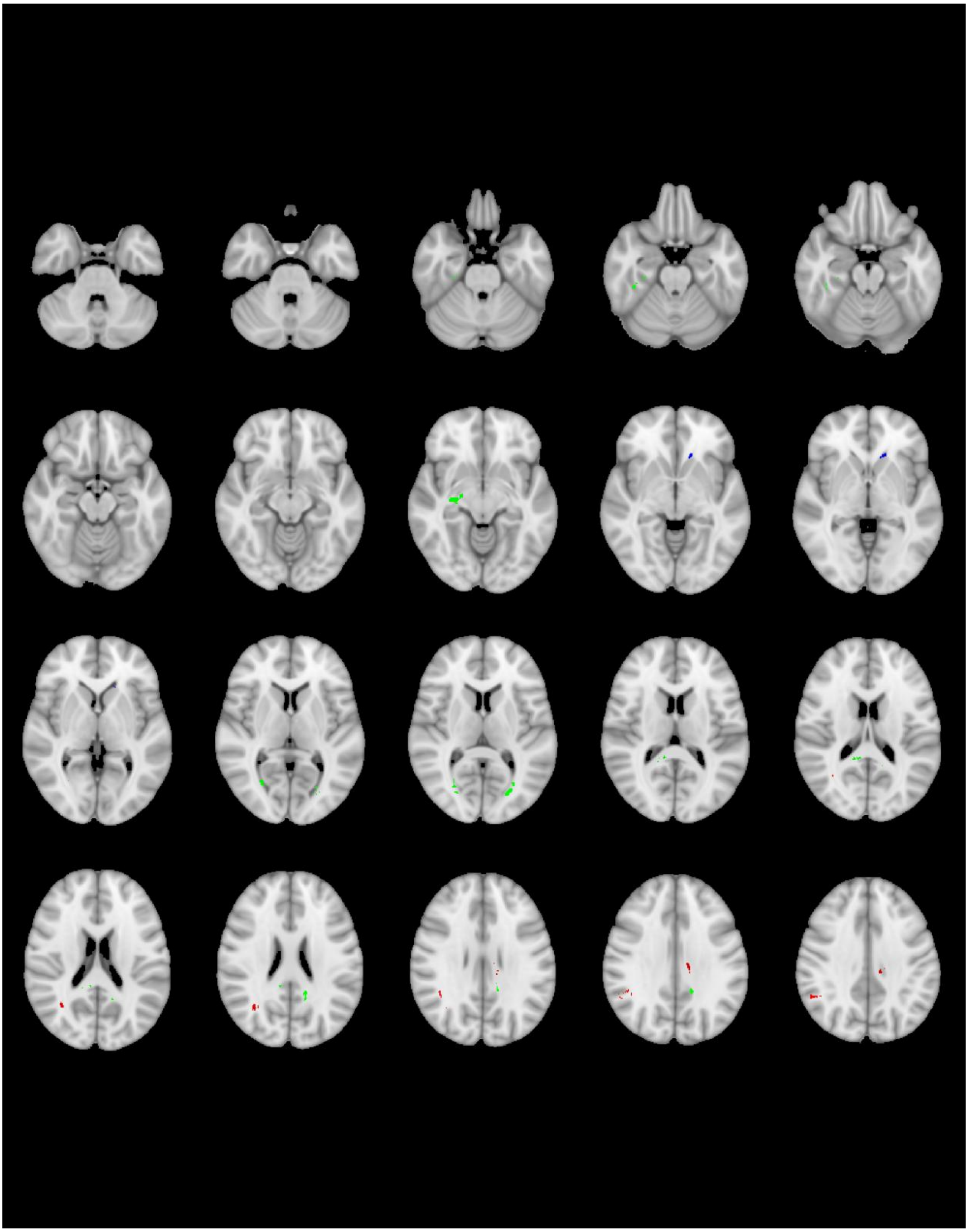

Figure 4 Shown is the negative decadic logarithm of the clusterwise p-values. The threshold for the clusters is a clusterwise p-value < 0.05 (>1.3 for the negative decadic logarithm).
Red marked spots indicate clusters with a negative correlation of the FA with the sum of NRS per hour per month
Green marked spots indicate clusters with a negative correlation of the AD with the sum of NRS per hour per month
Blue marked spots indicate clusters with a negative correlation of the RD with the sum of NRS per hour per month.

FA

Negative Cluster: 1

Name	Left-Cerebral-White-Matter
CWP	0.00542
Size	178 Voxel
TalX, TalY, TalZ	-17.79, -8.48, 47.06
**Linear Regression**	
Slope	-0.00002
Intercept	0.56168
R-value	-0.19617
P-Value	0.61297
**Spearman Regression**	
Spearman Correlation	-0.01667
P-Value	0.96605

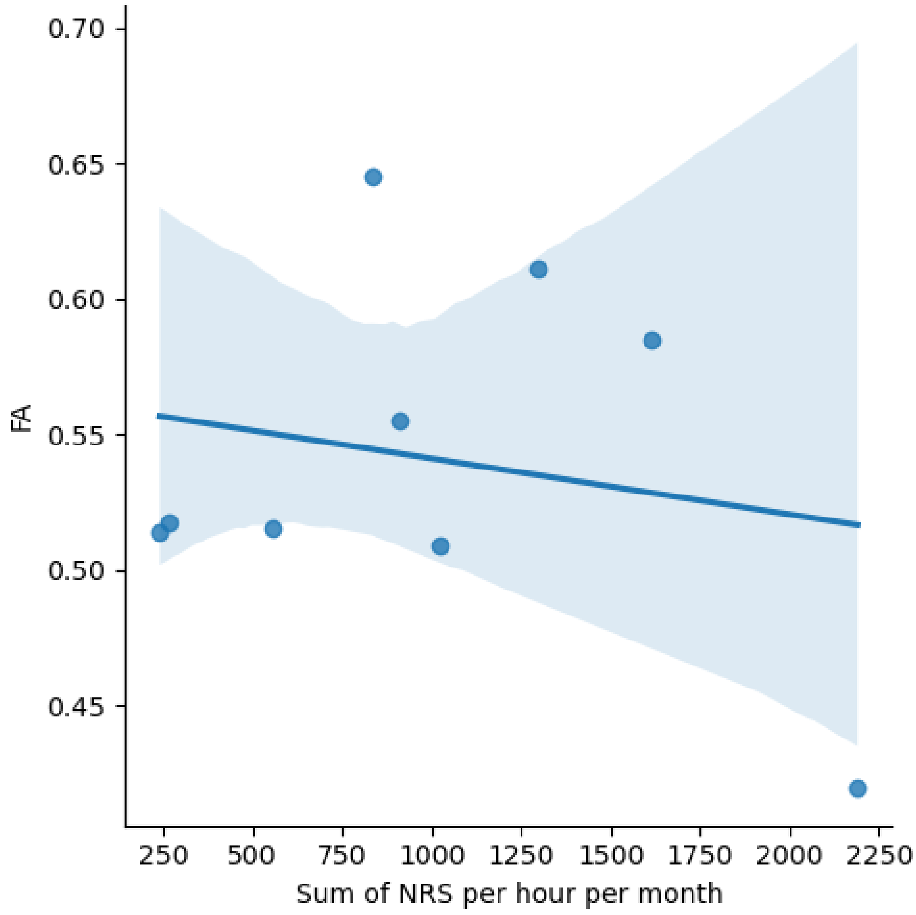

Negative Cluster: 2

Name	Right-Cerebral-White-Matter
CWP	0.01442
Size	160 Voxel
TalX, TalY, TalZ	54.68, -47.13, 32.91
**Linear Regression**	
Slope	-0.00004
Intercept	0.24068
R-value	-0.31328
P-Value	0.41171
**Spearman Regression**	
Spearman Correlation	-0.13333
P-Value	0.73237

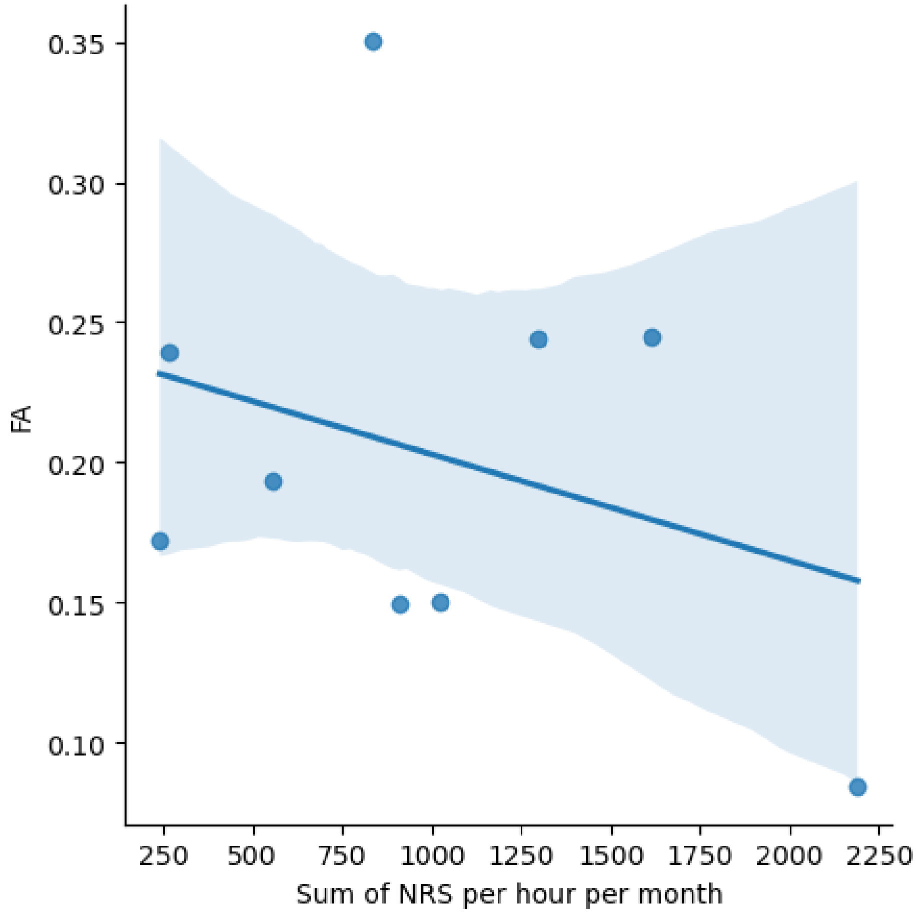

**Negative Cluster: 3**	
Name	Right-Cerebral-White-Matter
CWP	0.02283
Size	151 Voxel
TalX, TalY, TalZ	33.97, -58.09, 22.03
**Linear Regression**	
Slope	-0.00001
Intercept	0.52378
R-value	-0.07860
P-Value	0.84071
**Spearman Regression**	
Spearman Correlation	-0.11667
P-Value	0.76501

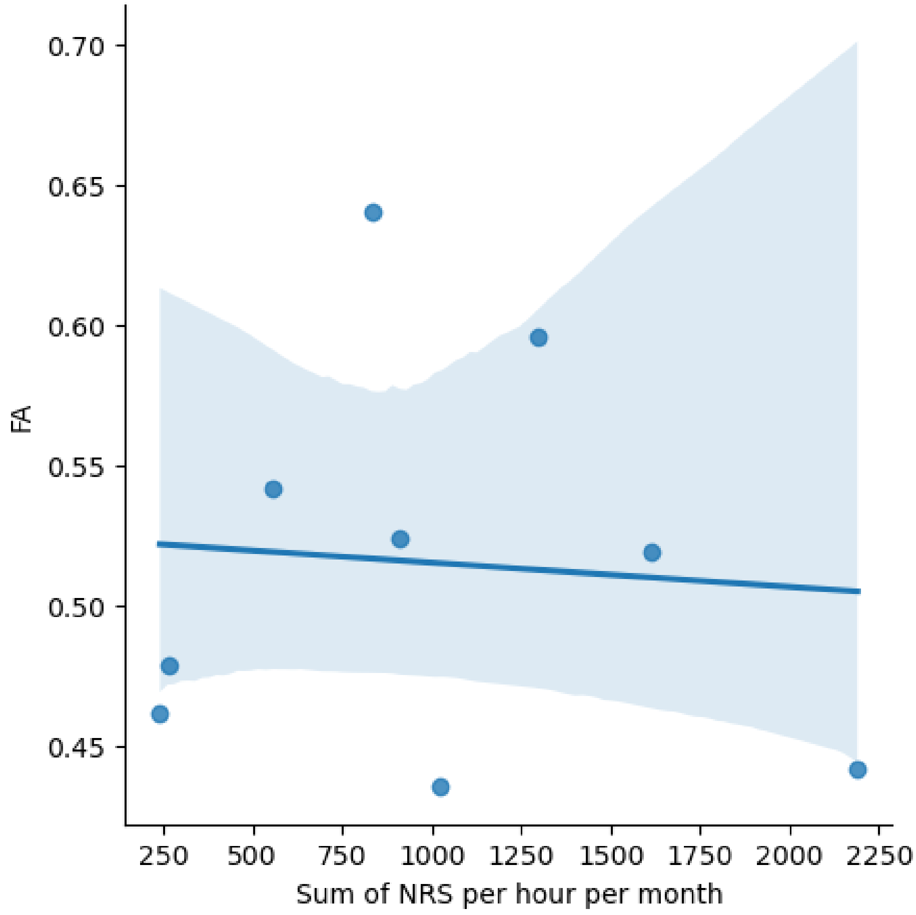

Negative Cluster: 4

Name	Left-Cerebral-White-Matter
CWP	0.03667
Size	143 Voxel
TalX, TalY, TalZ	-11.59, -21.1, 30.11
**Linear Regression**	
Slope	-0.00007
Intercept	0.68440
R-value	-0.70004
P-Value	0.03575
**Spearman Regression**	
Spearman Correlation	-0.48333
P-Value	0.18747

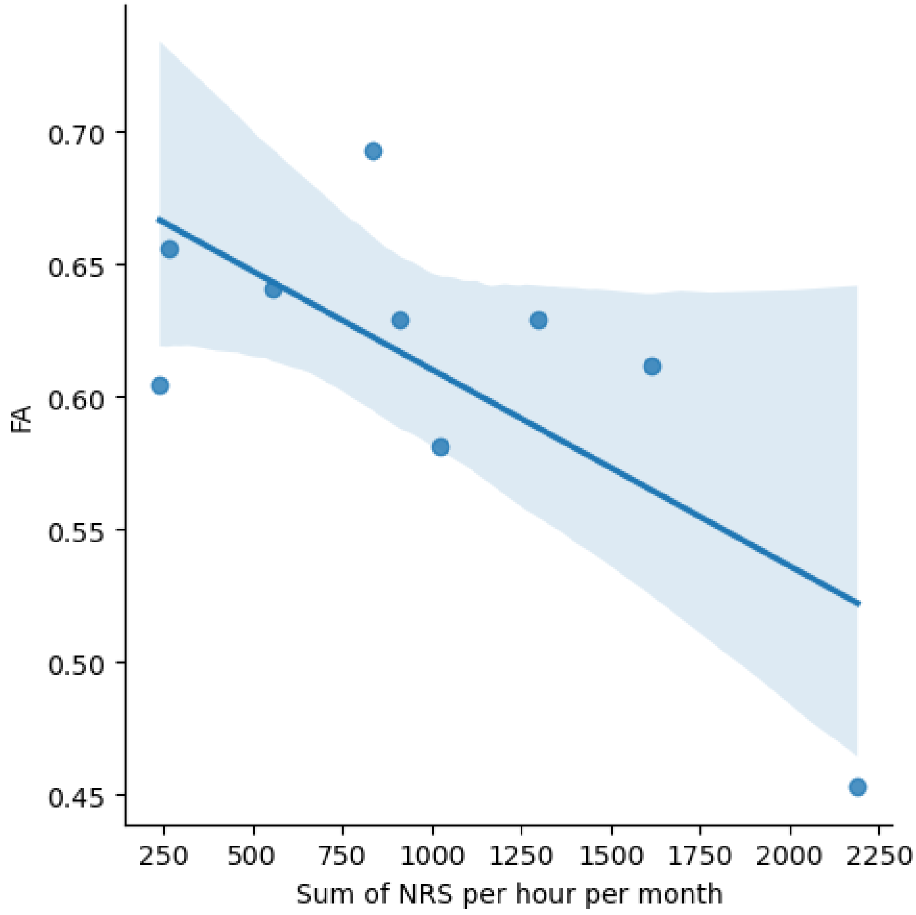

AD

**Negative Cluster: 1**	
Name	Left-Cerebral-White-Matter
CWP	0.00008
Size	203 Voxel
TalX, TalY, TalZ	-15.86, -18.23, 46.57
**Linear Regression**	
Slope	-0.00004
Intercept	1.07165
R-value	-0.24869
P-Value	0.51877
**Spearman Regression**	
Spearman Correlation	-0.16667
P-Value	0.66823

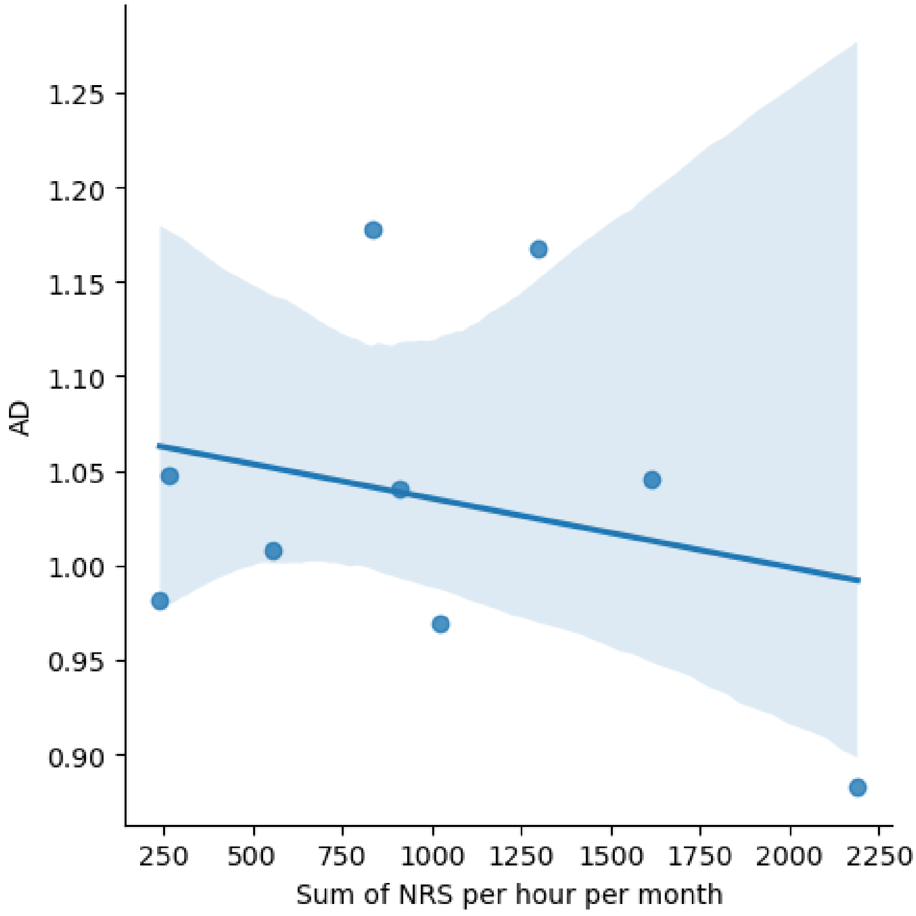

**Negative Cluster: 2**	
Name	Left-Cerebral-White-Matter
CWP	0.00025
Size	153 Voxel
TalX, TalY, TalZ	-13.64, -43.64, 25.48
**Linear Regression**	
Slope	-0.00004
Intercept	1.22951
R-value	-0.28263
P-Value	0.46120
**Spearman Regression**	
Spearman Correlation	-0.01667
P-Value	0.96605

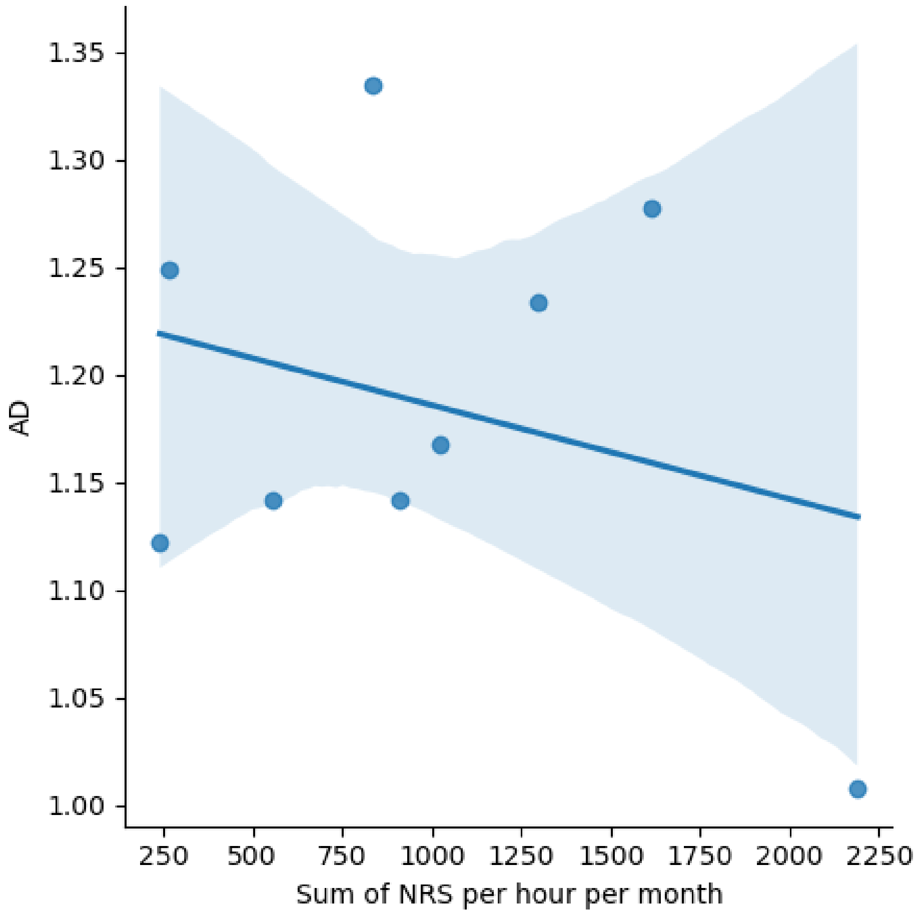

Negative Cluster: 3

Name	Left-Cerebral-White-Matter
CWP	0.00125
Size	140 Voxel
TalX, TalY, TalZ	-28.37, -66.58, 9.62
**Linear Regression**	
Slope	-0.00008
Intercept	1.41480
R-value	-0.12890
P-Value	0.74102
**Spearman Regression**	
Spearman Correlation	-0.06667
P-Value	0.86469

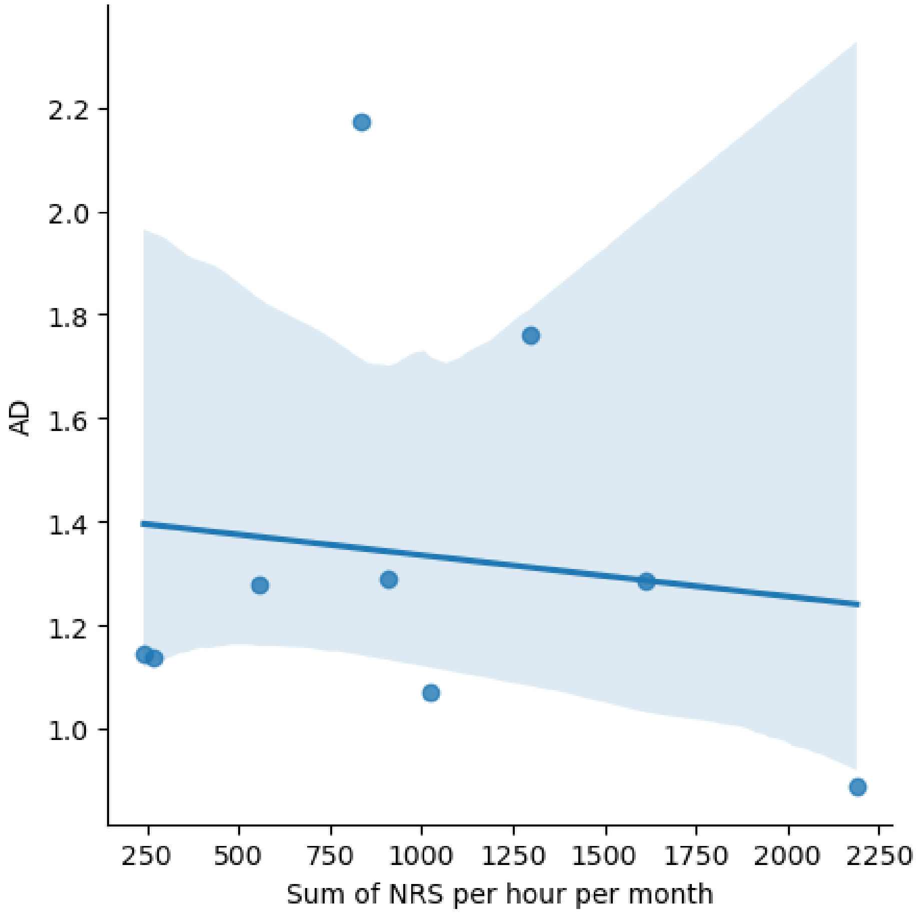

Negative Cluster: 4

Name	ctx-rh-parahippocampal
CWP	0.00583
Size	123 Voxel
TalX, TalY, TalZ	30.05, -26.23, -18.96
**Linear Regression**	
Slope	-0.00013
Intercept	1.20438
R-value	-0.43067
P-Value	0.24719
**Spearman Regression**	
Spearman Correlation	-0.08333
P-Value	0.83121

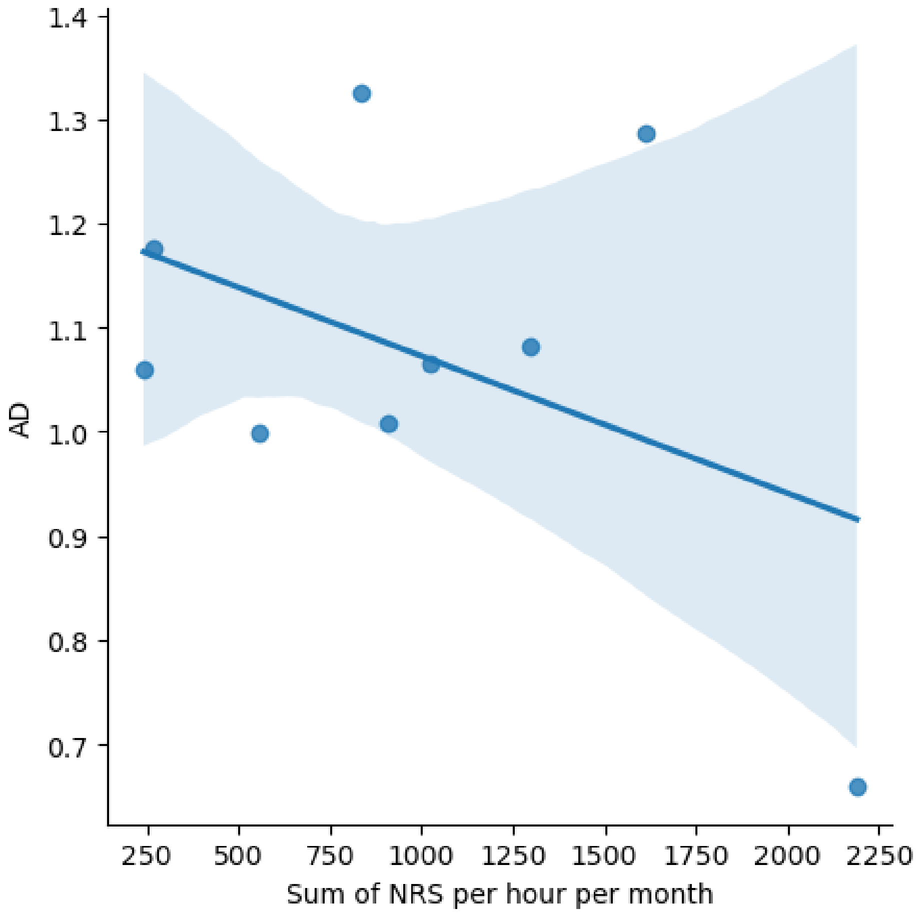

Negative Cluster: 5

Name	Right-VentralDC
CWP	0.01217
Size	113 Voxel
TalX, TalY, TalZ	25.83, -21.58, -5.71
**Linear Regression**	
Slope	-0.00014
Intercept	1.57042
R-value	-0.55493
P-Value	0.12094
**Spearman Regression**	
Spearman Correlation	-0.63333
P-Value	0.06709

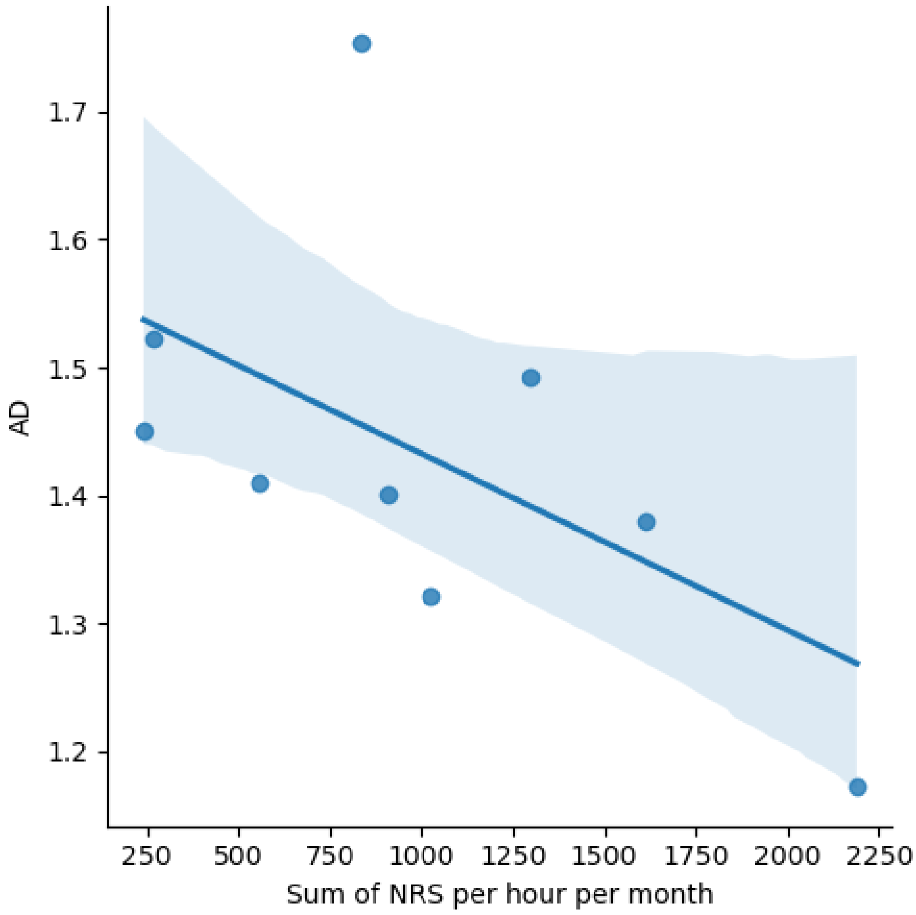

Negative Cluster: 6

Name	Right-Cerebral-White-Matter
CWP	0.01217
Size	113 Voxel
TalX, TalY, TalZ	26.27, -67.52, 6.63
**Linear Regression**	
Slope	0.00008
Intercept	1.42660
R-value	0.09965
P-Value	0.79866
**Spearman Regression**	
Spearman Correlation	0.16667
P-Value	0.66823

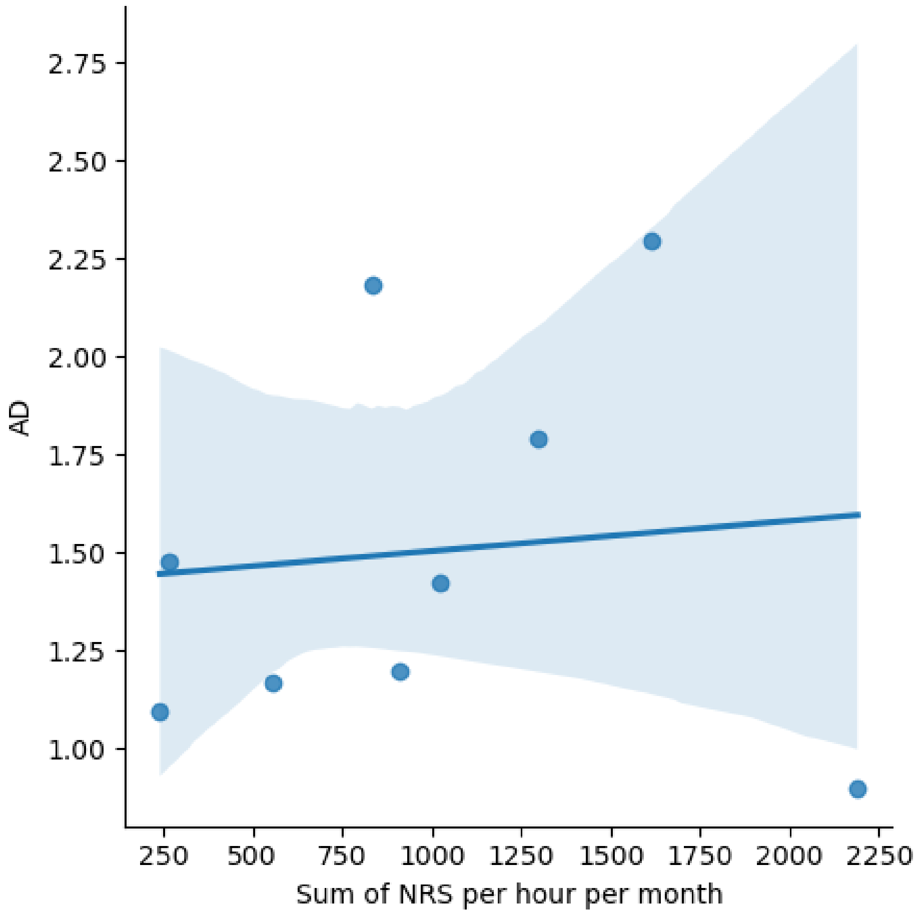

Negative Cluster: 7

Name	Right-Cerebral-White-Matter
CWP	0.01417
Size	111 Voxel
TalX, TalY, TalZ	10.39, -41.59, 15.52

Linear Regression

Slope	0.00000
Intercept	1.60031
R-value	0.00920
P-Value	0.98126

Spearman Regression

Spearman Correlation	0.16667
P-Value	0.66823

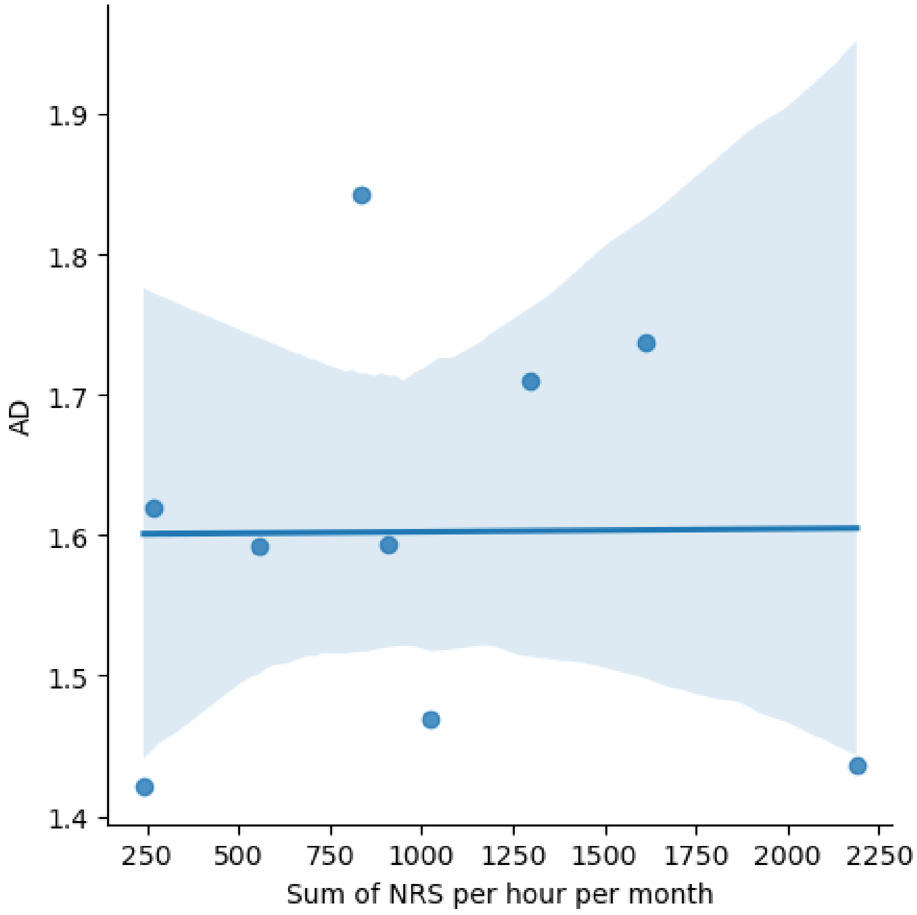

RD

**Negative Cluster: 1**	
Name	Left-Caudate
CWP	0.01742
Size	99 Voxel
TalX, TalY, TalZ	-12.57, 21.59, -7.1
**Linear Regression**	
Slope	-0.00005
Intercept	1.68908
R-value	-0.07257
P-Value	0.85280
**Spearman Regression**	
Spearman Correlation	0.11667
P-Value	0.76501

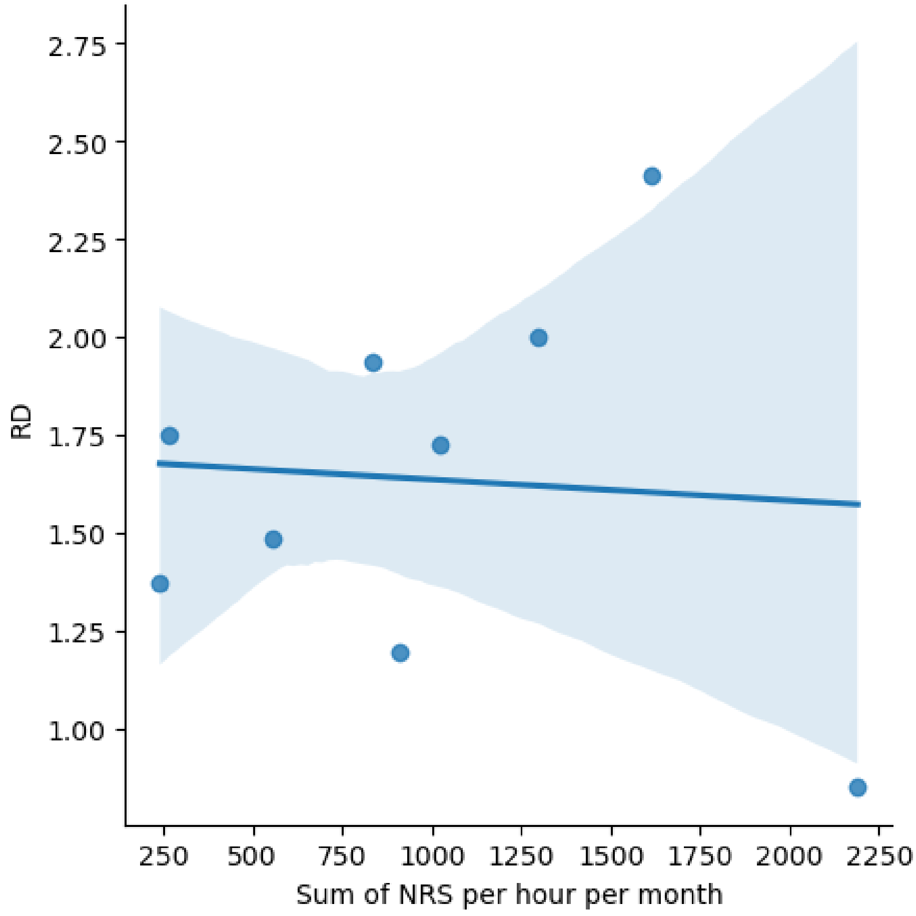

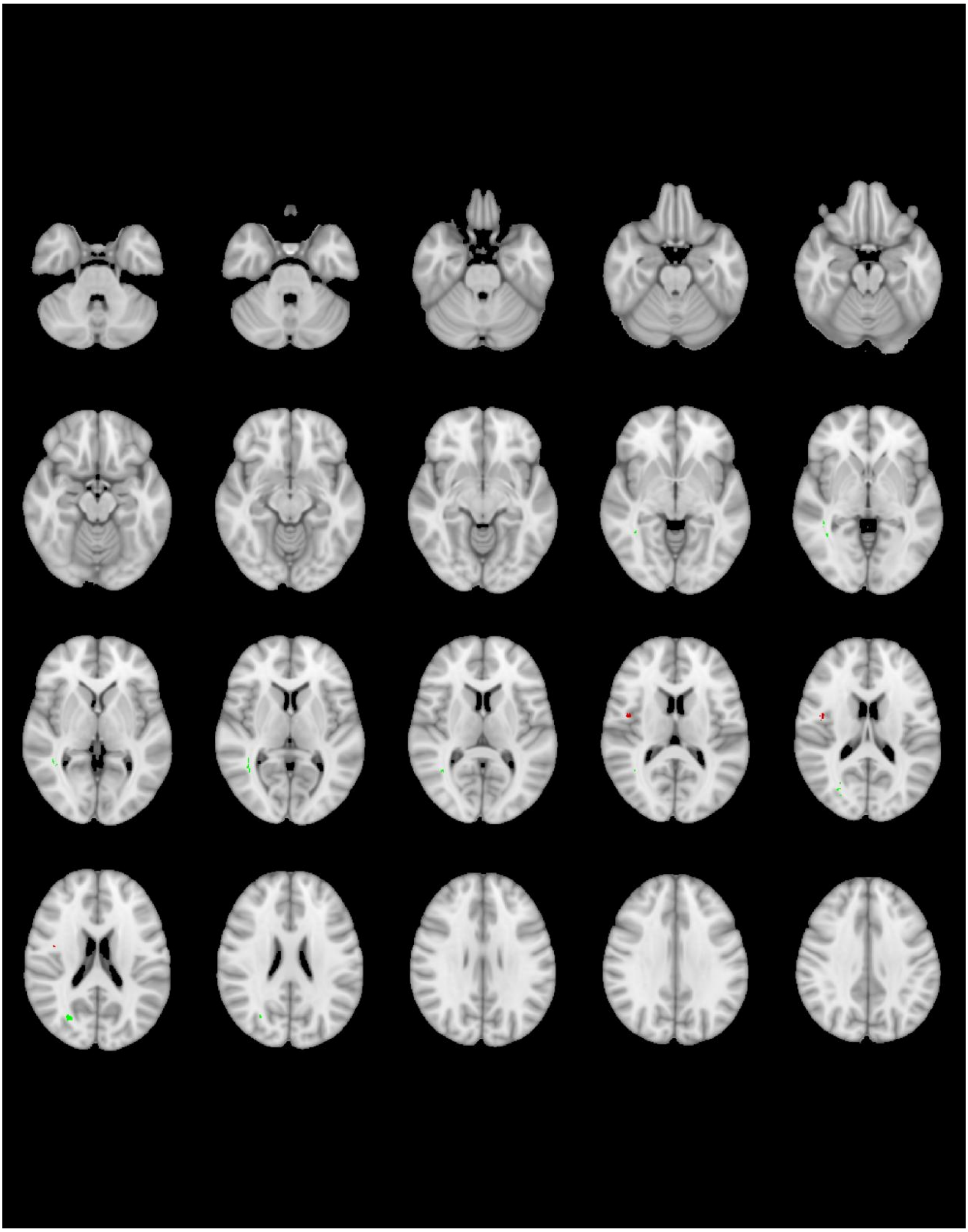

Figure 5 Shown is the negative decadic logarithm of the clusterwise p-values. The threshold for the clusters is a clusterwise p-value < 0.05 (>1.3 for the negative decadic logarithm).
Red marked spots indicate clusters with a positive correlation of the NDI with the hours with headache per month.
Green marked spots indicate clusters with a positive correlation of the ODI with the hours with headache per month.

NDI

**Positive Cluster: 1**	
Name	ctx-rh-precuneus
CWP	0.00108
Size	140 Voxel
TalX, TalY, TalZ	8.58, -62.05, 50.51
**Linear Regression**	
Slope	0.00082
Intercept	0.06991
R-value	0.55642
P-Value	0.11973
**Spearman Regression**	
Spearman Correlation	0.33333
P-Value	0.38071

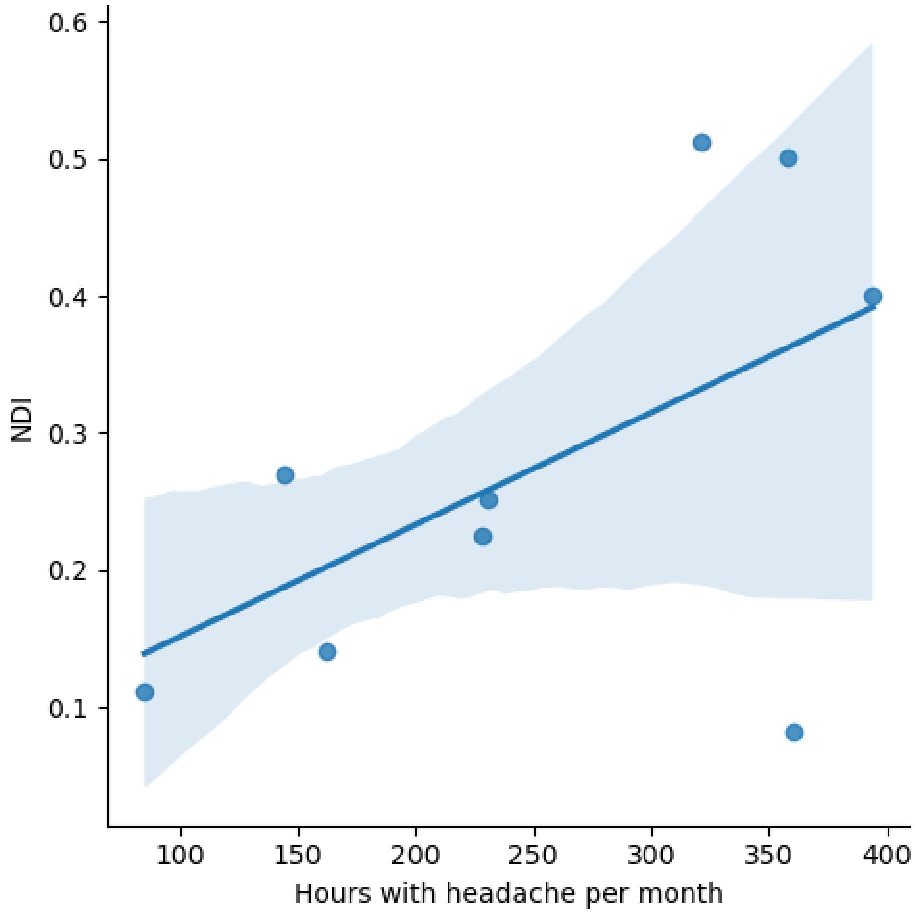

Positive Cluster: 2

Name	ctx-rh-postcentral
CWP	0.03267
Size	100 Voxel
TalX, TalY, TalZ	44.47, -4.49, 10.71
**Linear Regression**	
Slope	0.00015
Intercept	0.40577
R-value	0.13072
P-Value	0.73746
**Spearman Regression**	
Spearman Correlation	0.20000
P-Value	0.60590

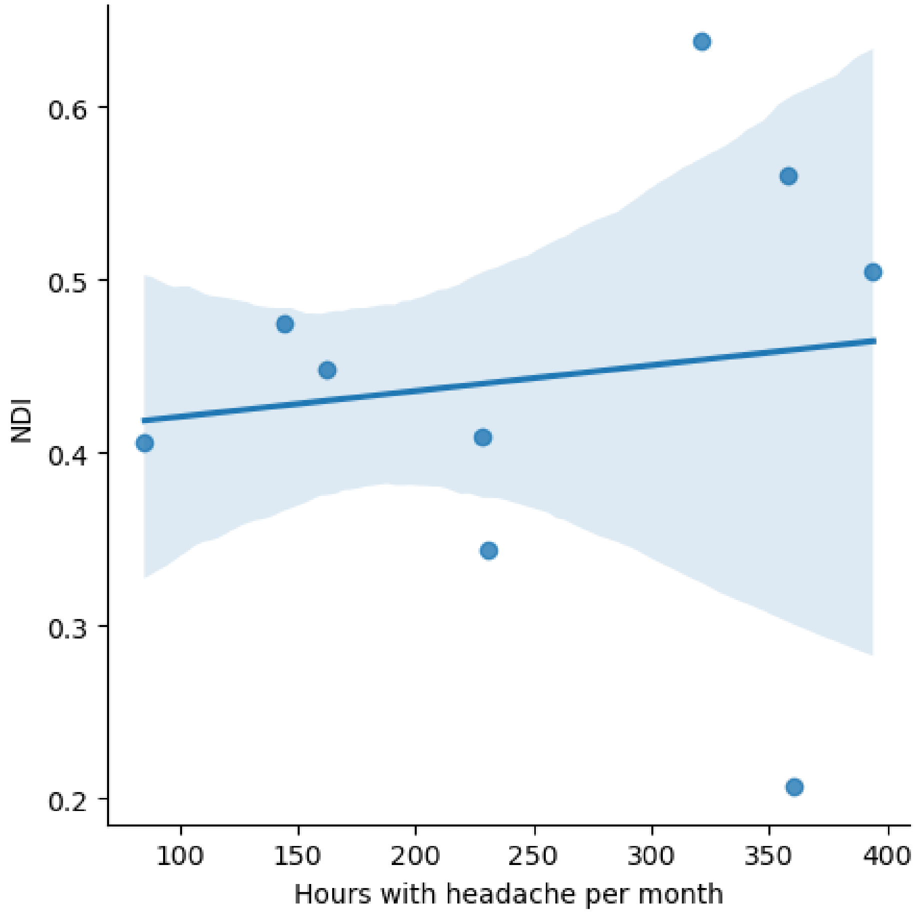

ODI

**Positive Cluster: 1**	
Name	Right-Cerebral-White-Matter
CWP	0.00067
Size	164 Voxel
TalX, TalY, TalZ	39.29, -51.24, 6.11
**Linear Regression**	
Slope	0.00013
Intercept	0.05800
R-value	0.52593
P-Value	0.14585
**Spearman Regression**	
Spearman Correlation	0.28333
P-Value	0.46003

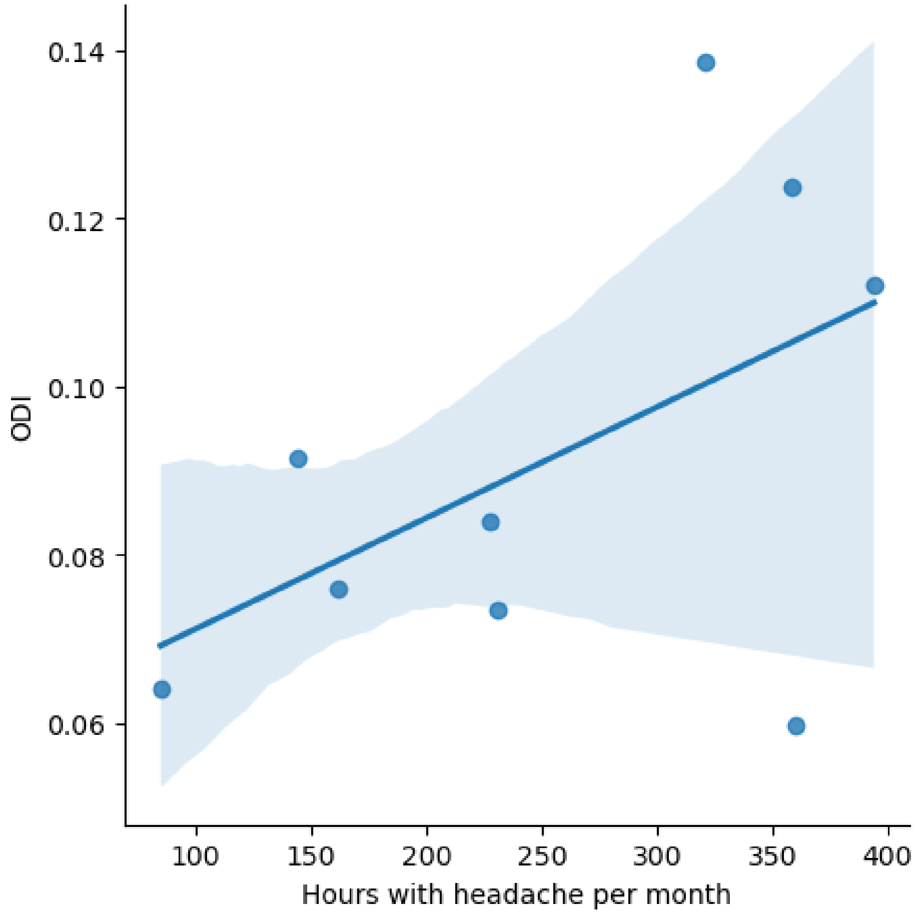

Positive Cluster: 2

Name	Right-Cerebral-White-Matter
CWP	0.00367
Size	142 Voxel
TalX, TalY, TalZ	25.0, -68.71, 20.51
**Linear Regression**	
Slope	0.00023
Intercept	0.04307
R-value	0.59953
P-Value	0.08794
**Spearman Regression**	
Spearman Correlation	0.36667
P-Value	0.33174

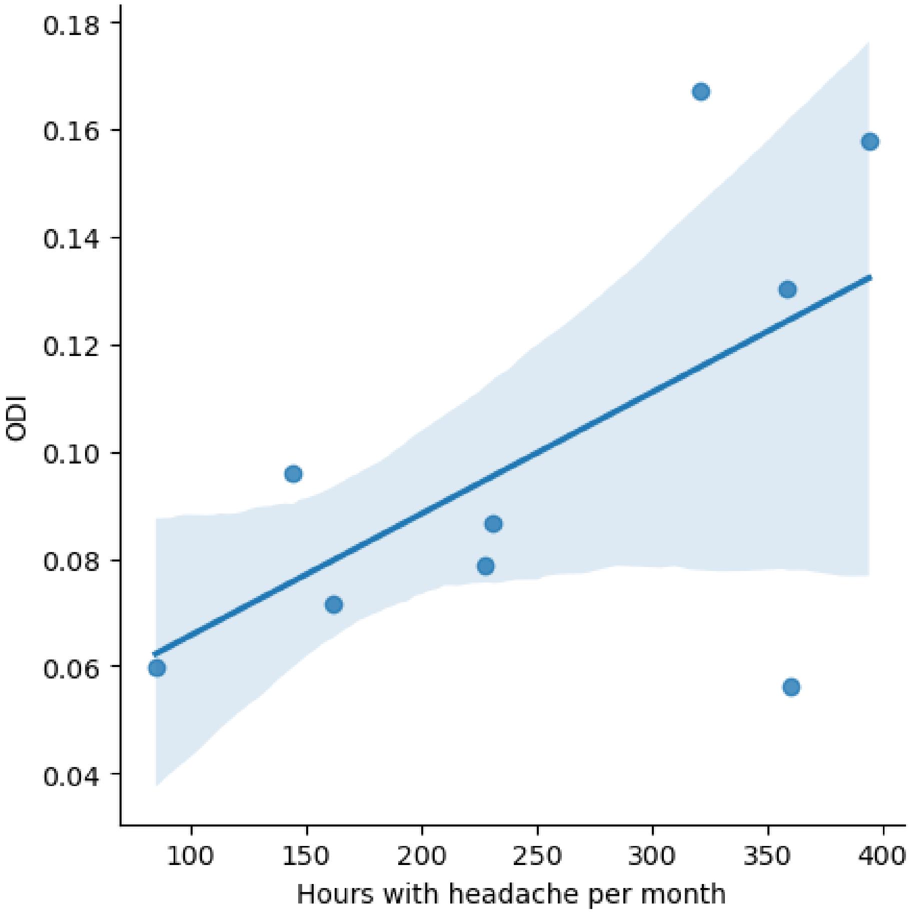

Positive Cluster: 3

Name	Right-Cerebral-White-Matter
CWP	0.01517
Size	123 Voxel
TalX, TalY, TalZ	9.65, -62.25, 46.84
**Linear Regression**	
Slope	0.00054
Intercept	0.14811
R-value	0.48165
P-Value	0.18924
**Spearman Regression**	
Spearman Correlation	0.38333
P-Value	0.30850

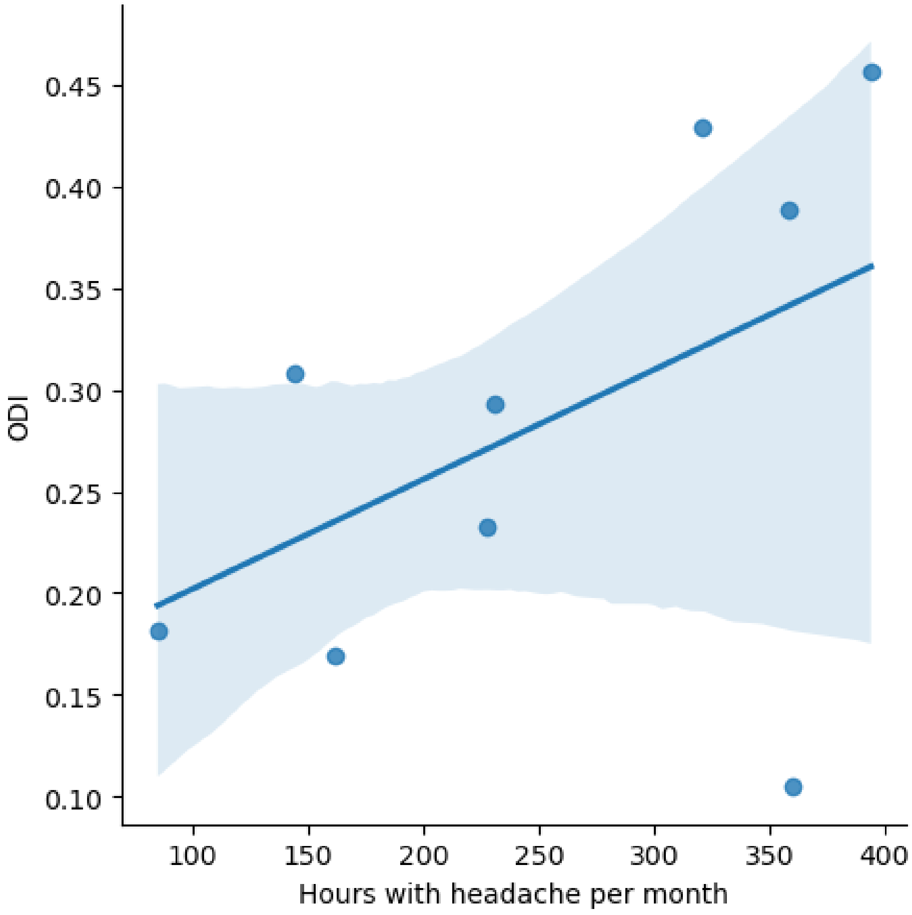

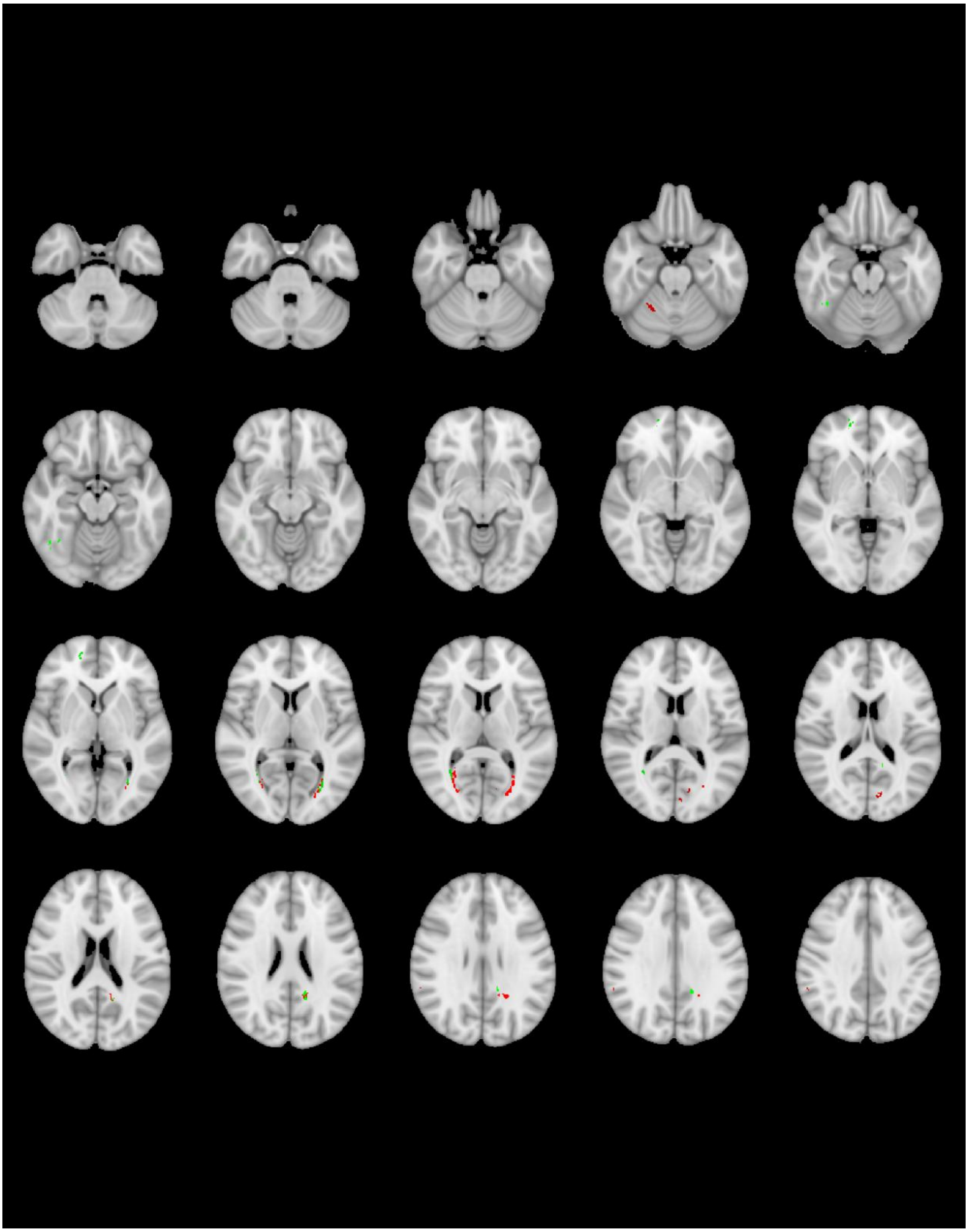

Figure 6 Shown is the negative decadic logarithm of the clusterwise p-values. The threshold for the clusters is a clusterwise p-value < 0.05 (>1.3 for the negative decadic logarithm).
Red marked spots indicate clusters with a negative correlation of the NDI with the hours with headache per month.
Green marked spots indicate clusters with a negative correlation of the ODI with the hours with headache per month.

NDI

**Negative Cluster: 1**	
Name	Left-Cerebral-White-Matter
CWP	0.00008
Size	312 Voxel
TalX, TalY, TalZ	-30.28, -61.81, 7.55
**Linear Regression**	
Slope	-0.00075
Intercept	0.55689
R-value	-0.53255
P-Value	0.13992
**Spearman Regression**	
Spearman Correlation	-0.48333
P-Value	0.18747

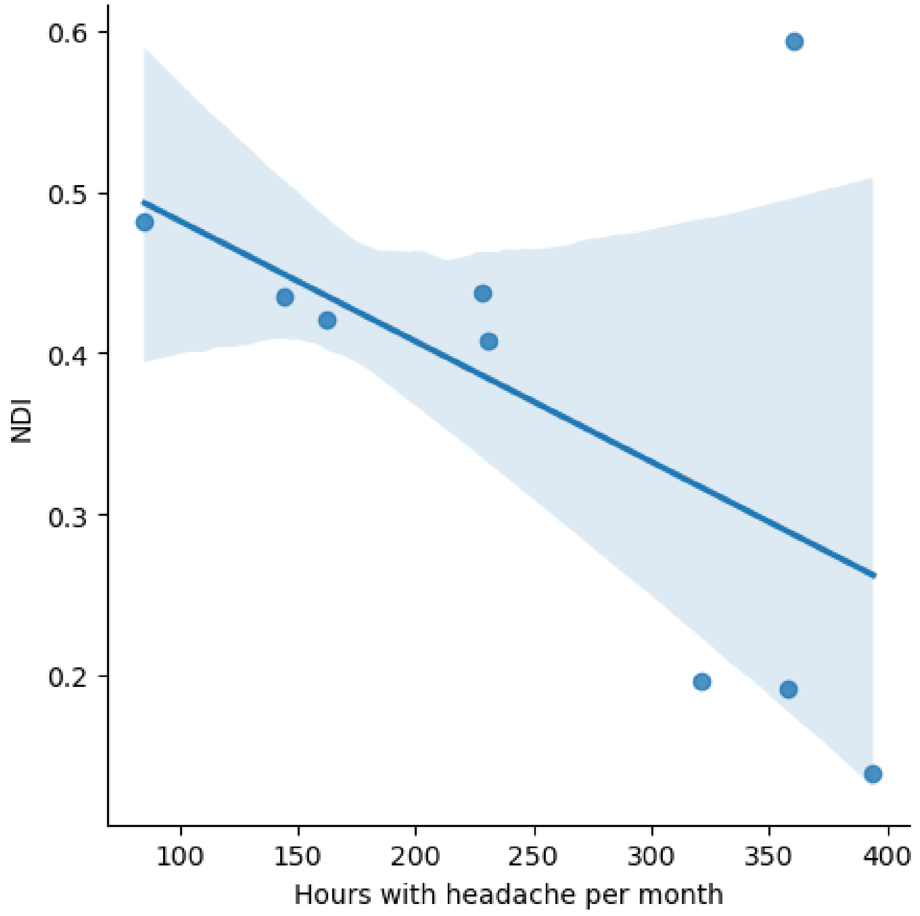

Negative Cluster: 2

Name	Left-Cerebral-White-Matter
CWP	0.00008
Size	236 Voxel
TalX, TalY, TalZ	-13.63, -50.56, 22.99
**Linear Regression**	
Slope	-0.00043
Intercept	0.64511
R-value	-0.61769
P-Value	0.07630
**Spearman Regression**	
Spearman Correlation	-0.51667
P-Value	0.15439

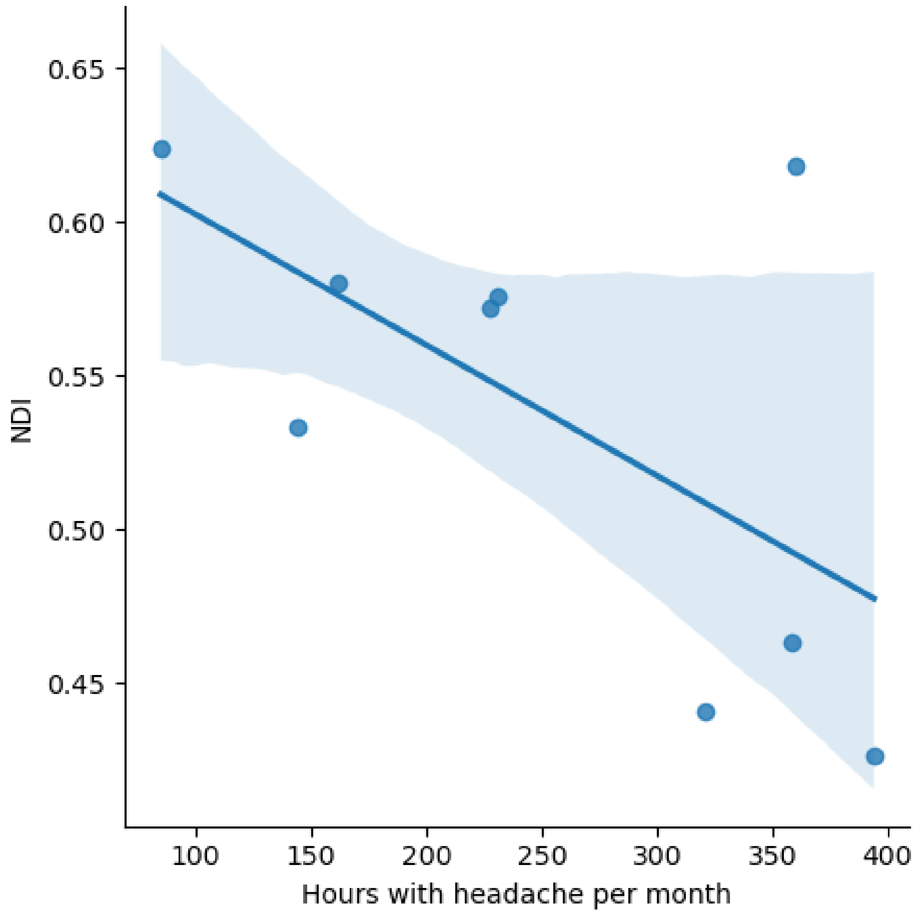

Negative Cluster: 3

Name	Right-Cerebral-White-Matter
CWP	0.00008
Size	236 Voxel
TalX, TalY, TalZ	27.3, -64.68, 5.6
**Linear Regression**	
Slope	-0.00075
Intercept	0.54008
R-value	-0.50902
P-Value	0.16165
**Spearman Regression**	
Spearman Correlation	-0.41667
P-Value	0.26459

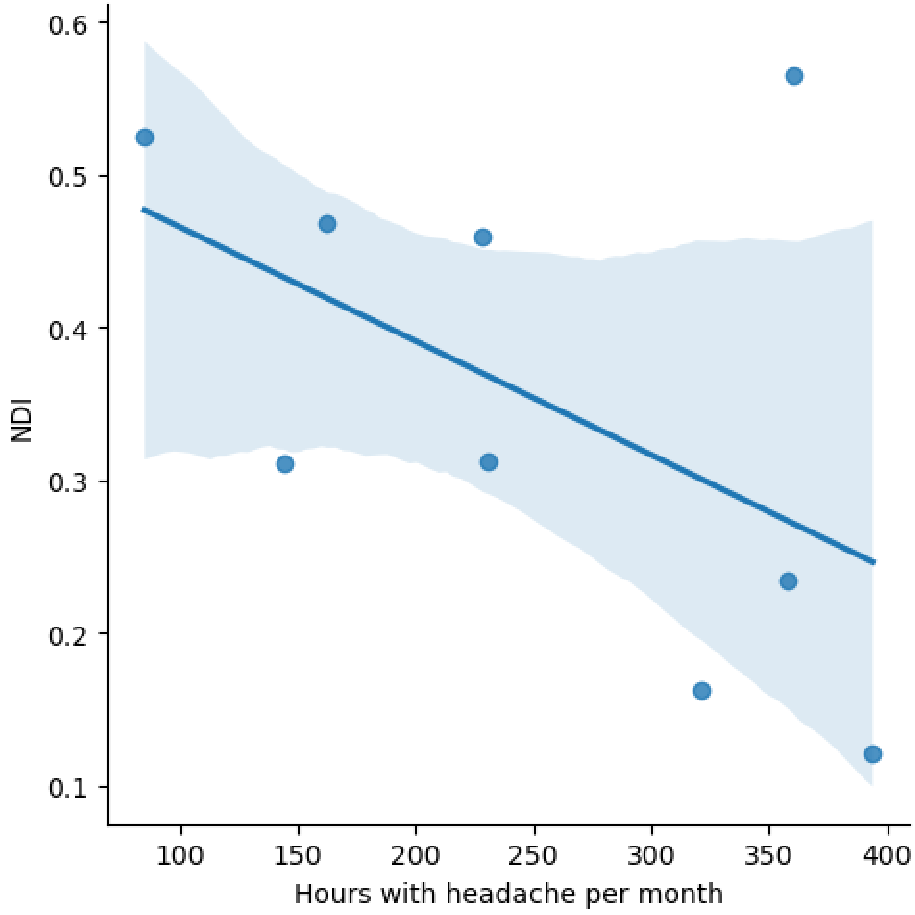

Negative Cluster: 4

Name	Unknown
CWP	0.02525
Size	103 Voxel
TalX, TalY, TalZ	47.61, -43.75, 40.07
**Linear Regression**	
Slope	-0.00068
Intercept	0.50451
R-value	-0.51066
P-Value	0.16008
**Spearman Regression**	
Spearman Correlation	-0.36667
P-Value	0.33174

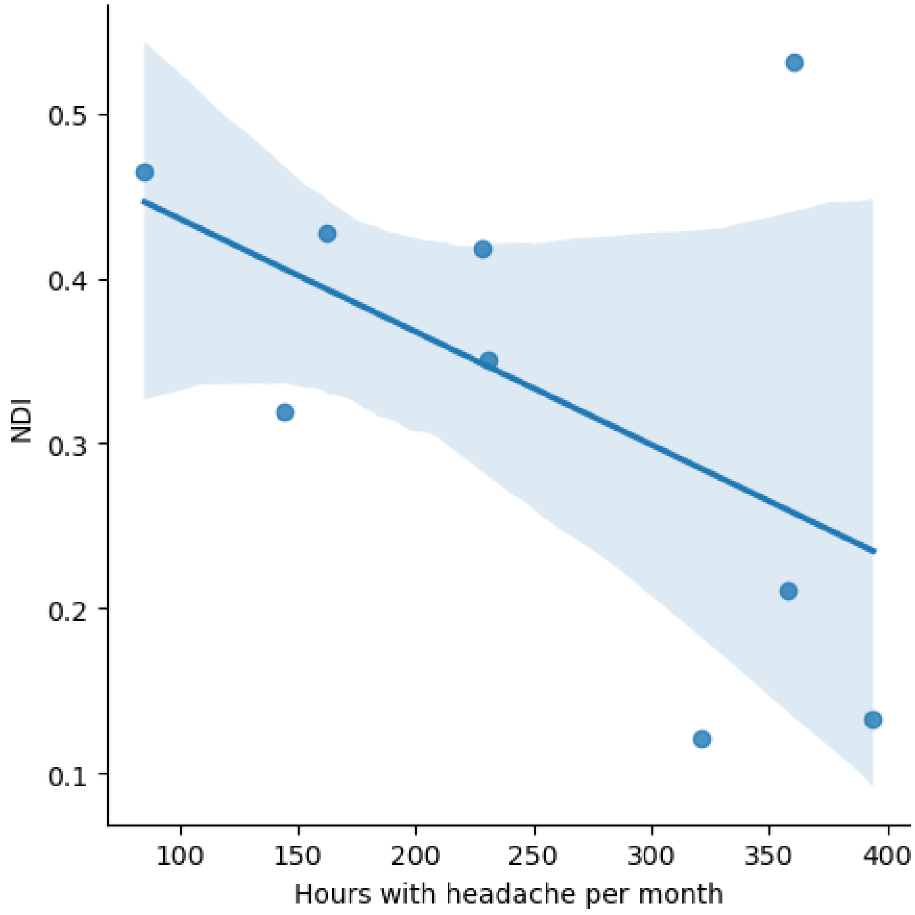

Negative Cluster: 5

Name	ctx-lh-pericalcarine
CWP	0.03892
Size	98 Voxel
TalX, TalY, TalZ	-10.66, -75.3, 14.81
**Linear Regression**	
Slope	-0.00093
Intercept	0.66297
R-value	-0.65819
P-Value	0.05394
**Spearman Regression**	
Spearman Correlation	-0.51667
P-Value	0.15439

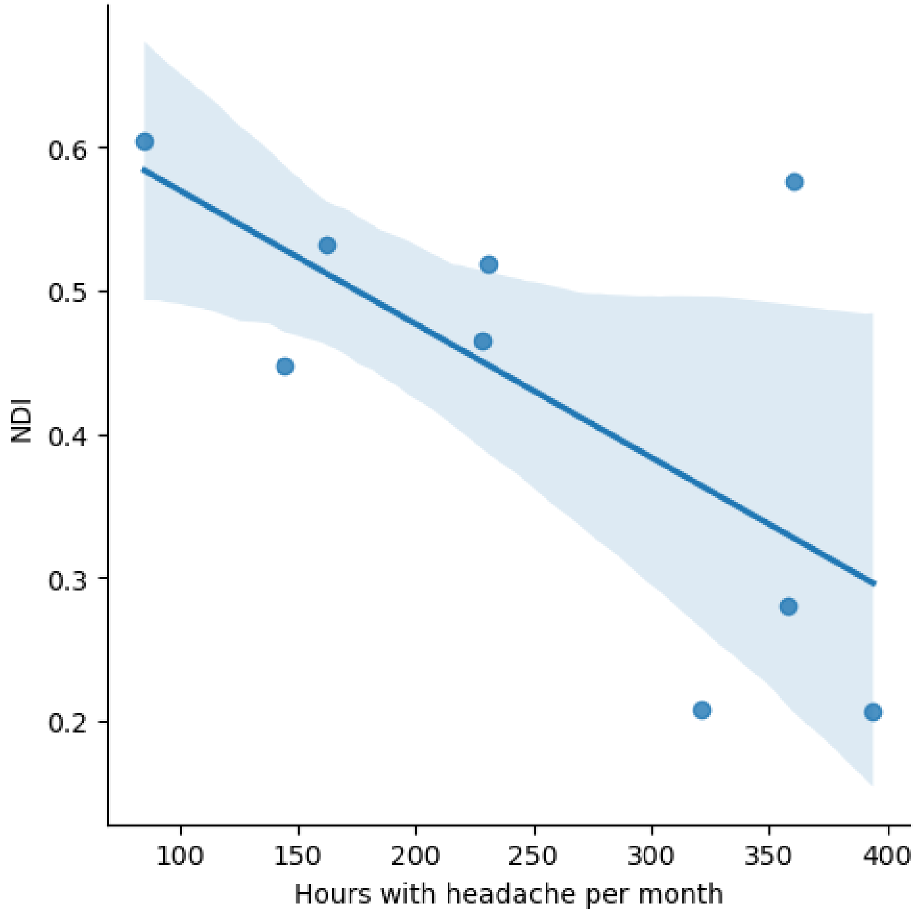

Negative Cluster: 6

Name	Right-Cerebellum-Cortex
CWP	0.04933
Size	95 Voxel
TalX, TalY, TalZ	25.88, -53.28, -17.87
**Linear Regression**	
Slope	-0.00024
Intercept	0.61232
R-value	-0.28574
P-Value	0.45605
**Spearman Regression**	
Spearman Correlation	-0.26667
P-Value	0.48792

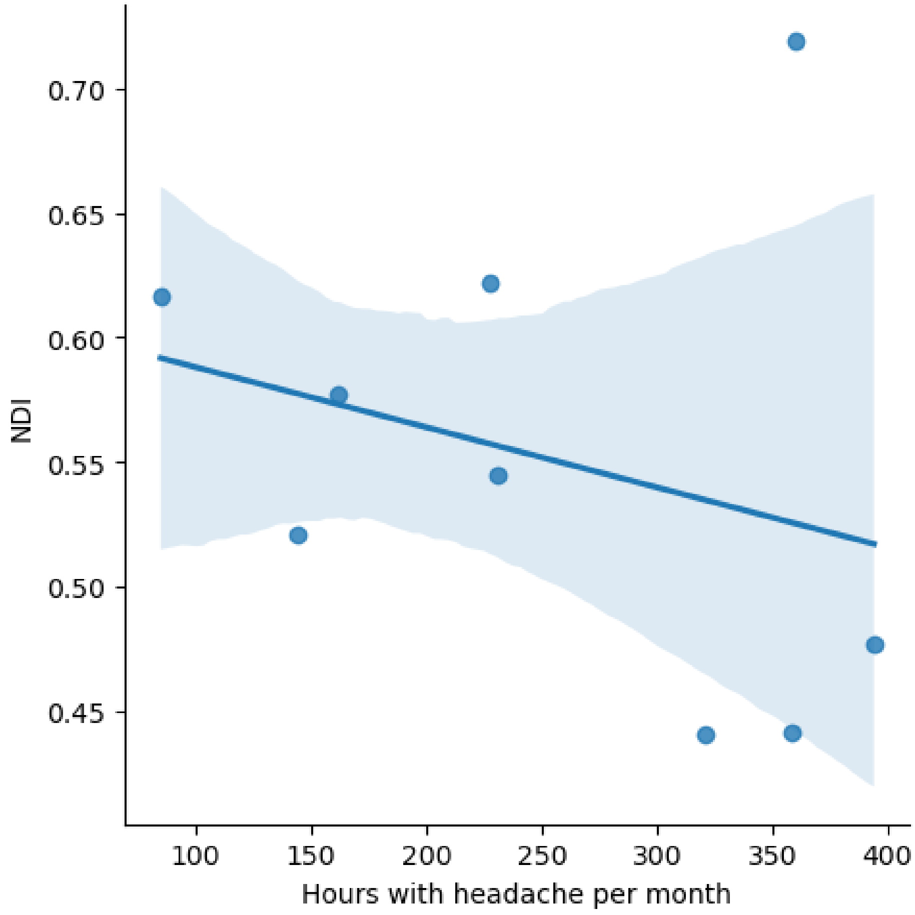

ODI

**Negative Cluster: 1**	
Name	Left-Cerebral-White-Matter
CWP	0.00008
Size	219 Voxel
TalX, TalY, TalZ	-15.86, -15.28, 47.37
**Linear Regression**	
Slope	-0.00009
Intercept	0.19508
R-value	-0.29675
P-Value	0.43809
**Spearman Regression**	
Spearman Correlation	-0.31667
P-Value	0.40640

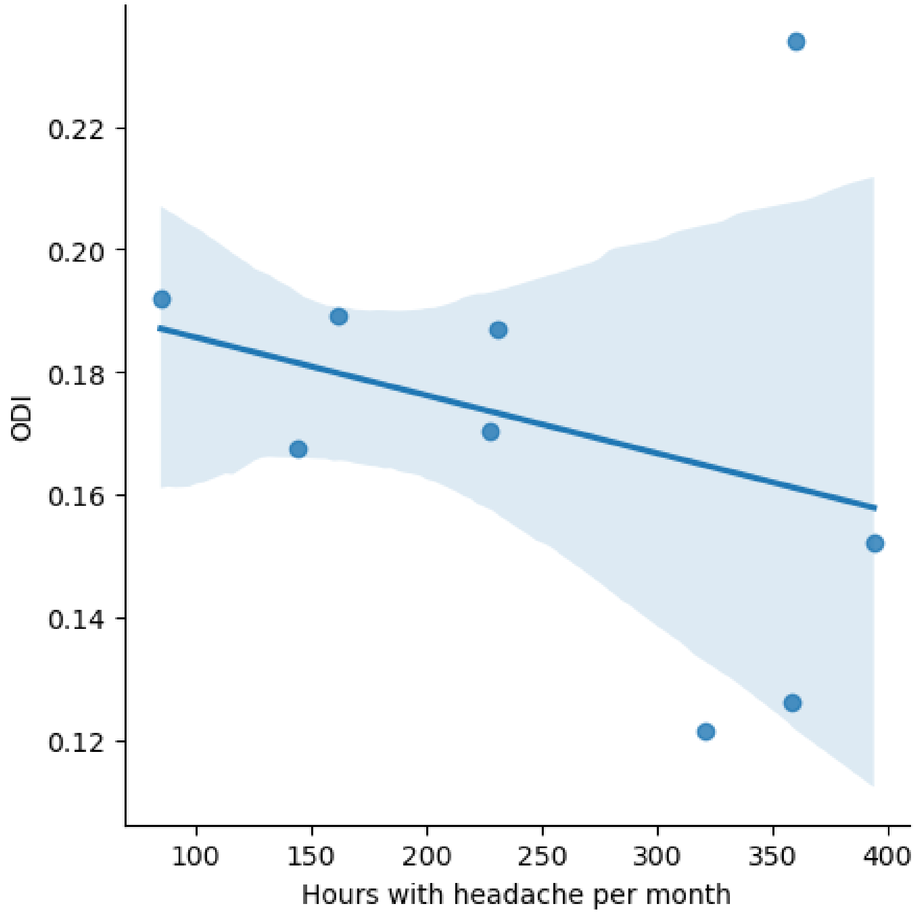

Negative Cluster: 2

Name	Left-Cerebral-White-Matter
CWP	0.00067
Size	163 Voxel
TalX, TalY, TalZ	-14.61, -50.6, 22.06
**Linear Regression**	
Slope	-0.00007
Intercept	0.15435
R-value	-0.19391
P-Value	0.61715
**Spearman Regression**	
Spearman Correlation	-0.26667
P-Value	0.48792

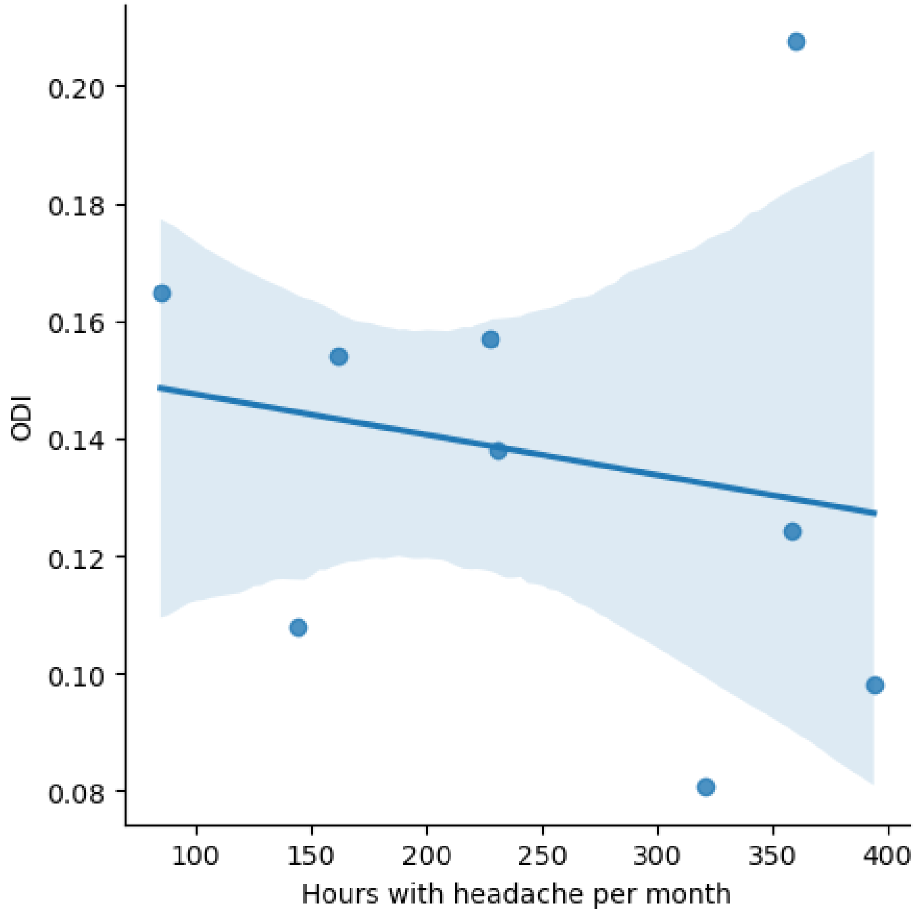

**Negative Cluster: 3**	
Name	ctx-rh-fusiform
CWP	0.00108
Size	159 Voxel
TalX, TalY, TalZ	40.69, -54.28, -14.29
**Linear Regression**	
Slope	-0.00078
Intercept	0.51776
R-value	-0.72802
P-Value	0.02616
**Spearman Regression**	
Spearman Correlation	-0.58333
P-Value	0.09919

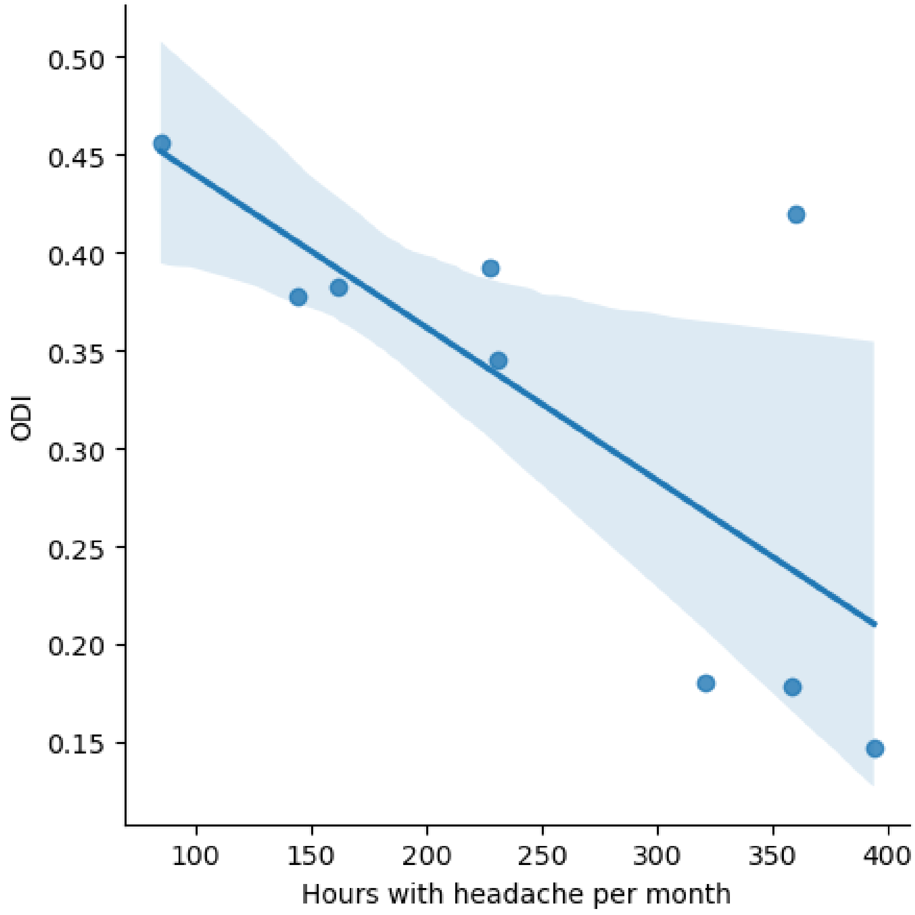

Negative Cluster: 4

Name	Right-Cerebral-White-Matter
CWP	0.02483
Size	117 Voxel
TalX, TalY, TalZ	18.32, 49.43, -4.56
**Linear Regression**	
Slope	-0.00051
Intercept	0.31644
R-value	-0.71698
P-Value	0.02971
**Spearman Regression**	
Spearman Correlation	-0.60000
P-Value	0.08762

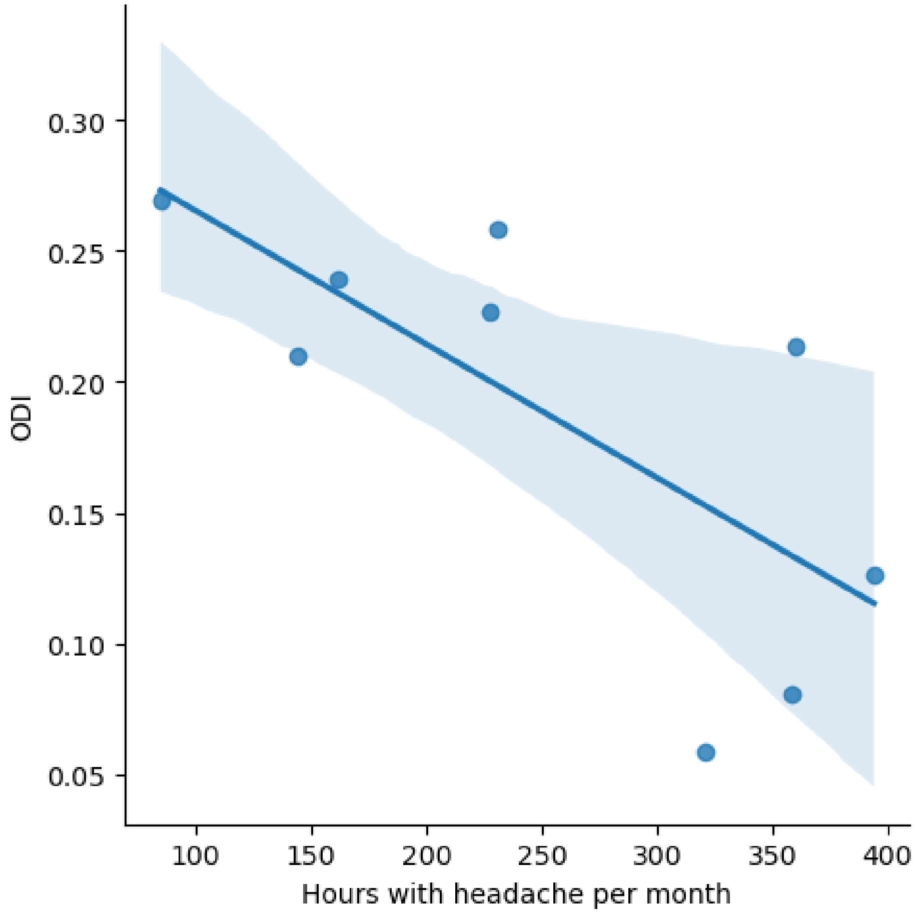

Negative Cluster: 5

Name	Left-Cerebral-White-Matter
CWP	0.03067
Size	114 Voxel
TalX, TalY, TalZ	-26.33, -68.74, 6.04
**Linear Regression**	
Slope	-0.00031
Intercept	0.20645
R-value	-0.51934
P-Value	0.15190
**Spearman Regression**	
Spearman Correlation	-0.38333
P-Value	0.30850

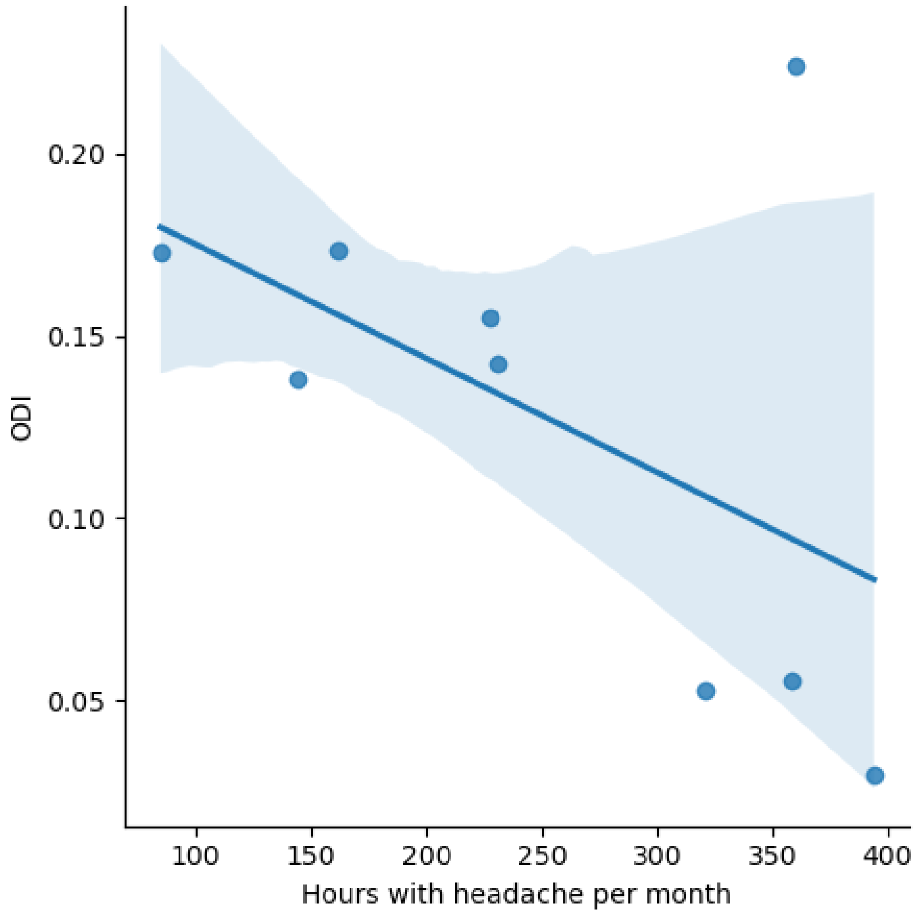

Negative Cluster: 6

Name	Right-Cerebral-White-Matter
CWP	0.03067
Size	114 Voxel
TalX, TalY, TalZ	29.2, -56.57, 12.67
**Linear Regression**	
Slope	-0.00018
Intercept	0.17044
R-value	-0.23990
P-Value	0.53411
**Spearman Regression**	
Spearman Correlation	-0.21667
P-Value	0.57551

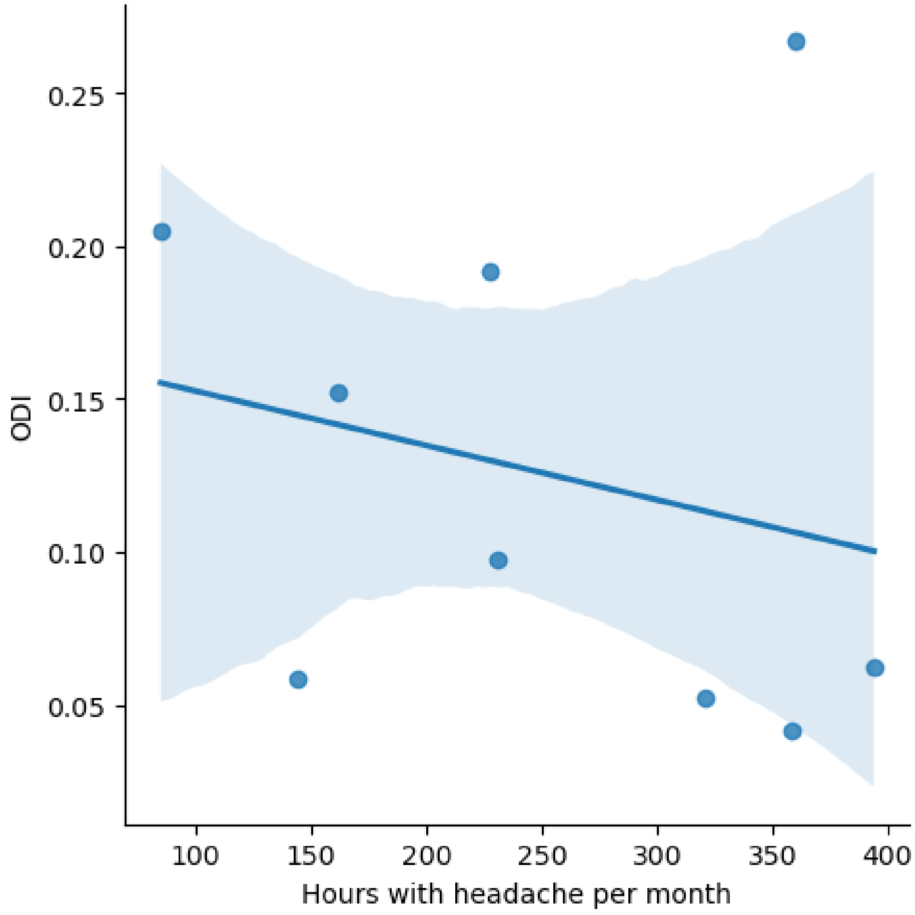

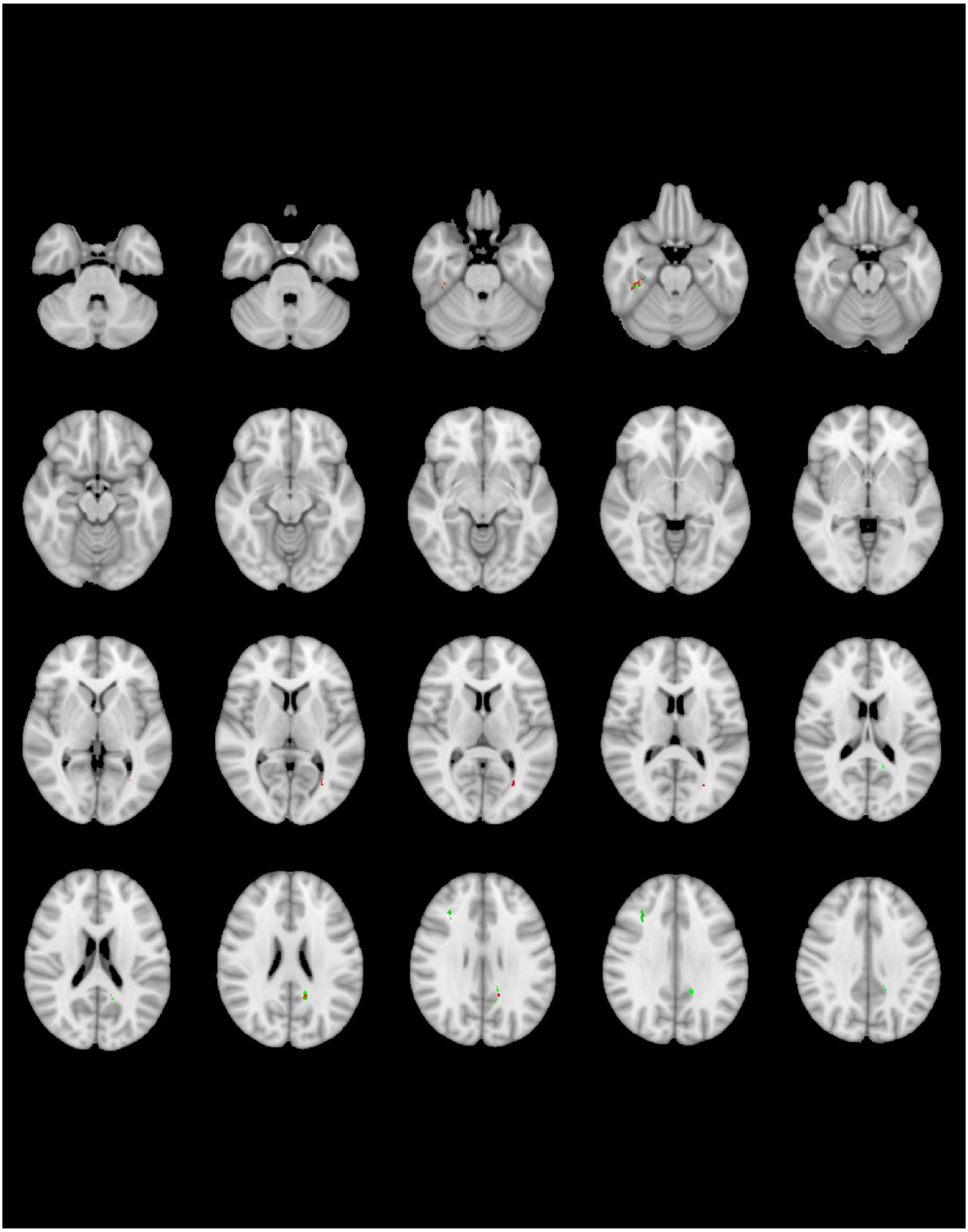

Figure 7 Shown is the negative decadic logarithm of the clusterwise p-values. The threshold for the clusters is a clusterwise p-value < 0.05 (>1.3 for the negative decadic logarithm).
Red marked spots indicate clusters with a positive correlation of the NDI with the sum of NRS per hour per month.
Green marked spots indicate clusters with a positive correlation of the ODI with the sum of NRS per hour per month.

NDI

**Positive Cluster: 1**	
Name	ctx-rh-fusiform
CWP	0.00075
Size	154 Voxel
TalX, TalY, TalZ	38.92, -34.1, -18.53
**Linear Regression**	
Slope	0.00012
Intercept	0.30708
R-value	0.49736
P-Value	0.17310
**Spearman Regression**	
Spearman Correlation	0.15000
P-Value	0.70009

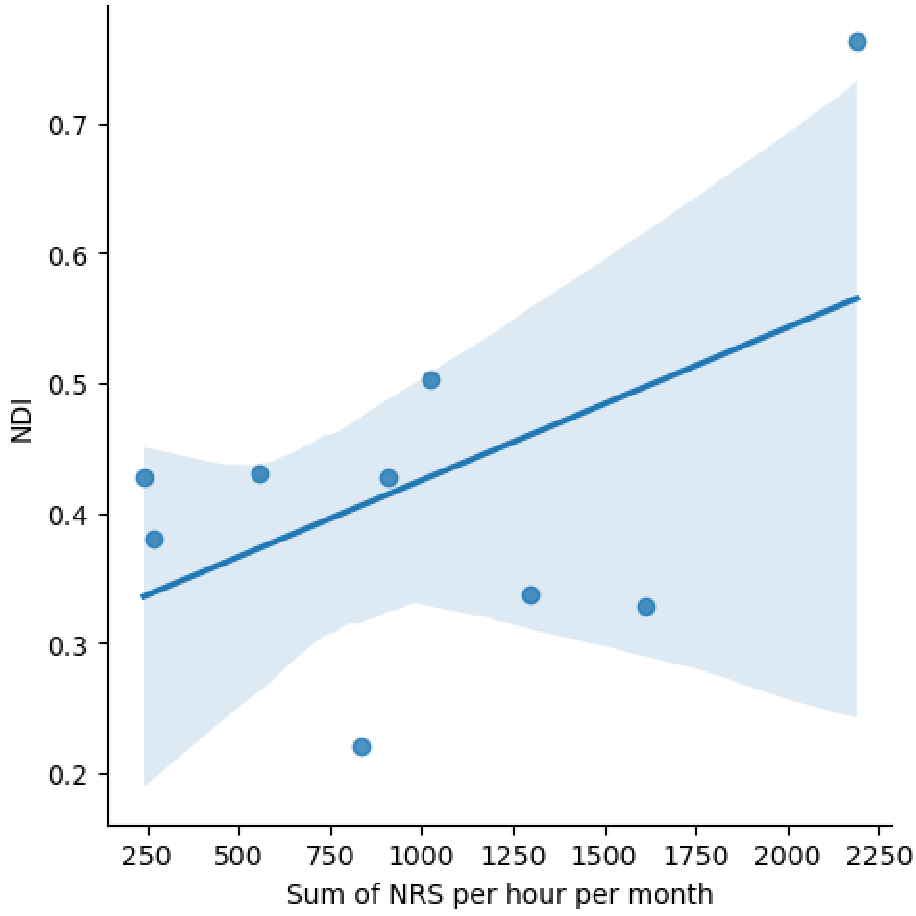

Positive Cluster: 2

Name	Left-Cerebral-White-Matter
CWP	0.01733
Size	109 Voxel
TalX, TalY, TalZ	-13.63, -50.56, 22.99
**Linear Regression**	
Slope	0.00001
Intercept	0.55397
R-value	0.08665
P-Value	0.82458
**Spearman Regression**	
Spearman Correlation	-0.03333
P-Value	0.93216

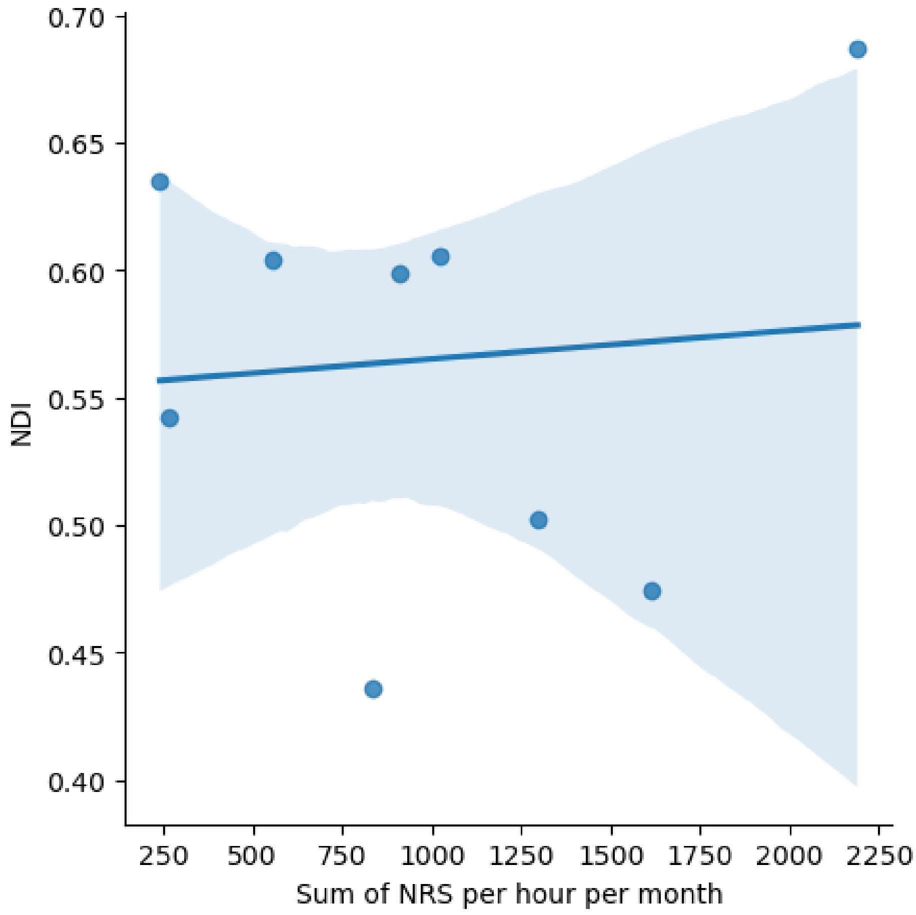

Positive Cluster: 3

Name	Left-Cerebral-White-Matter
CWP	0.02517
Size	104 Voxel
TalX, TalY, TalZ	-29.32, -66.67, 7.77
**Linear Regression**	
Slope	0.00001
Intercept	0.37536
R-value	0.04807
P-Value	0.90225
**Spearman Regression**	
Spearman Correlation	-0.25000
P-Value	0.51649

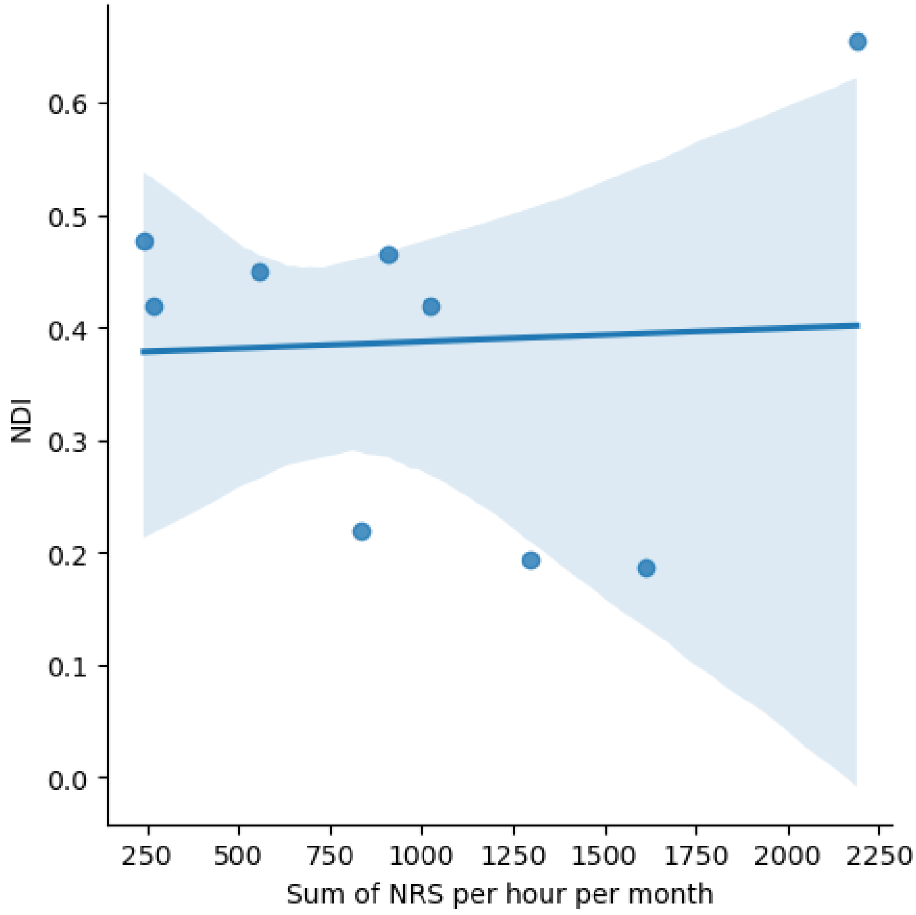

ODI

**Positive Cluster: 1**	
Name	Left-Cerebral-White-Matter
CWP	0.00008
Size	315 Voxel
TalX, TalY, TalZ	-14.61, -50.6, 22.06
**Linear Regression**	
Slope	0.00004
Intercept	0.13820
R-value	0.54072
P-Value	0.13280
**Spearman Regression**	
Spearman Correlation	0.23333
P-Value	0.54570

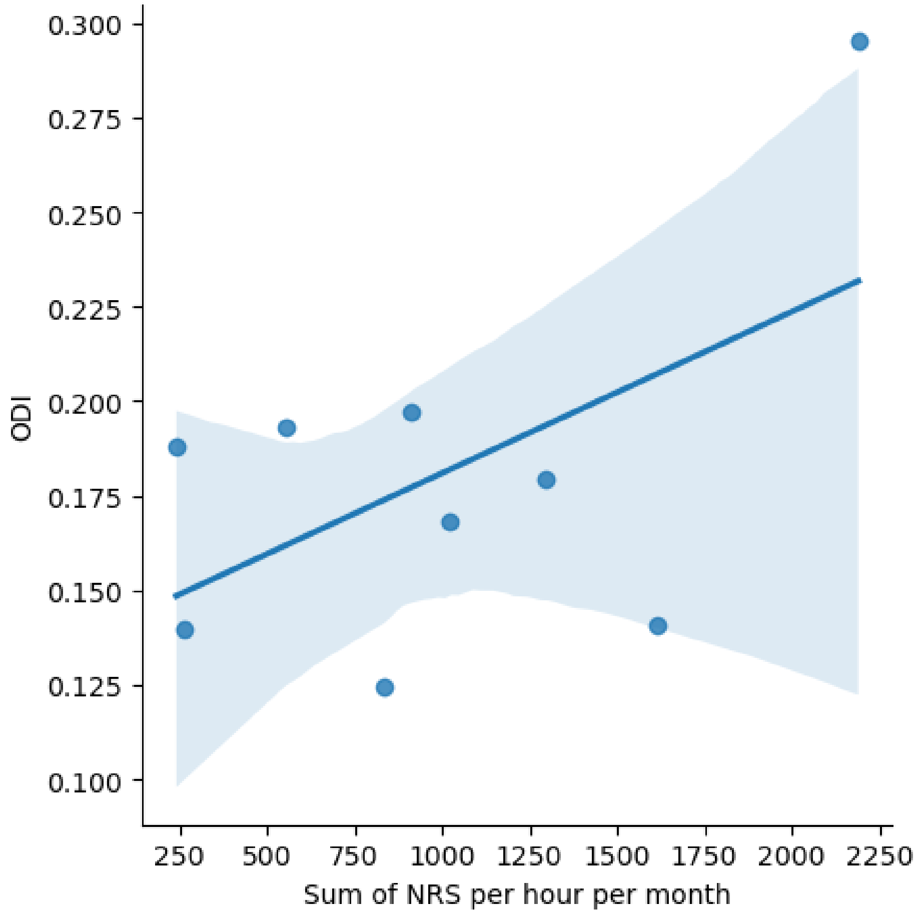

Positive Cluster: 2

Name	Left-Cerebral-White-Matter
CWP	0.00008
Size	207 Voxel
TalX, TalY, TalZ	-15.8, -17.41, 43.76
**Linear Regression**	
Slope	0.00003
Intercept	0.15543
R-value	0.45091
P-Value	0.22316
**Spearman Regression**	
Spearman Correlation	0.13333
P-Value	0.73237

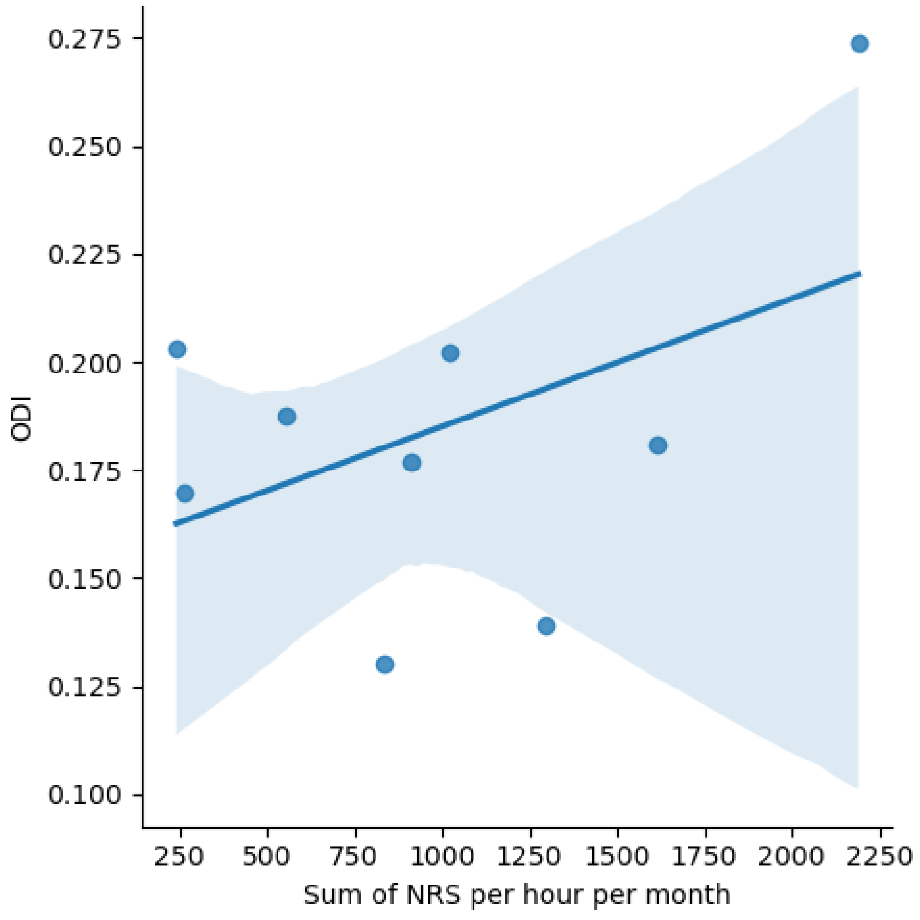

Positive Cluster: 3

Name	Right-Cerebral-White-Matter
CWP	0.02817
Size	116 Voxel
TalX, TalY, TalZ	12.93, -12.77, 47.61
**Linear Regression**	
Slope	0.00006
Intercept	0.16142
R-value	0.74535
P-Value	0.02117
**Spearman Regression**	
Spearman Correlation	0.48333
P-Value	0.18747

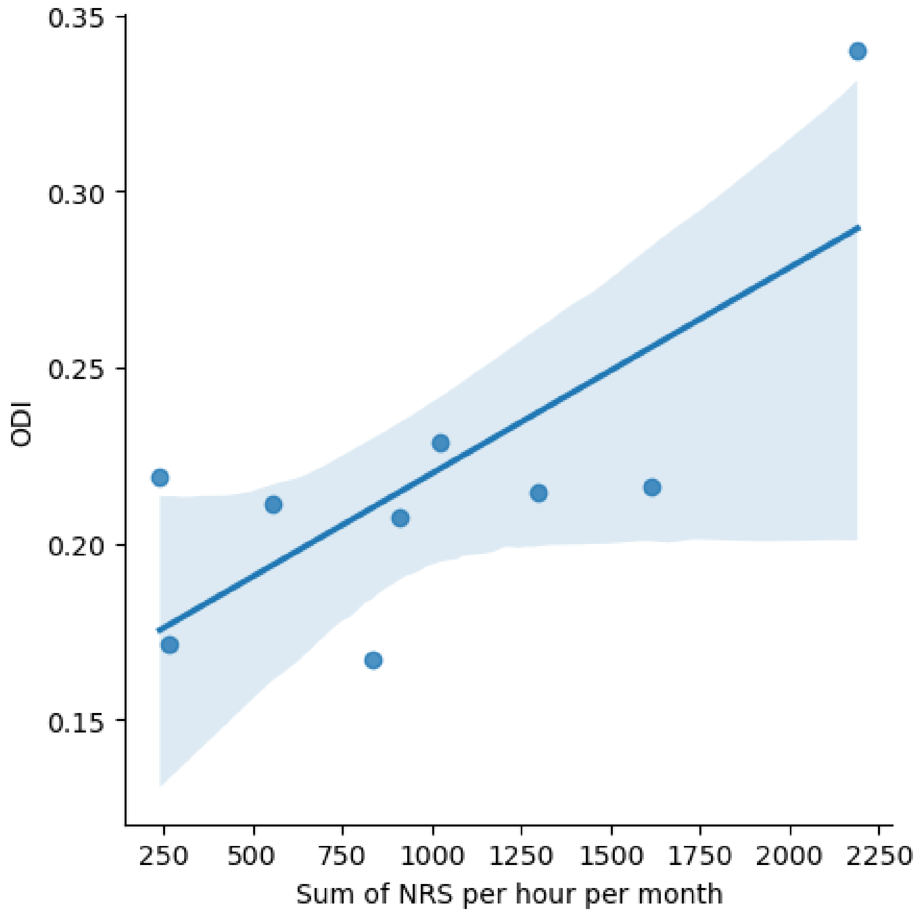

Positive Cluster: 4

Name	Unknown
CWP	0.03717
Size	112 Voxel
TalX, TalY, TalZ	32.05, -31.23, -21.26
**Linear Regression**	
Slope	0.00006
Intercept	0.25617
R-value	0.38187
P-Value	0.31051
**Spearman Regression**	
Spearman Correlation	0.05000
P-Value	0.89835

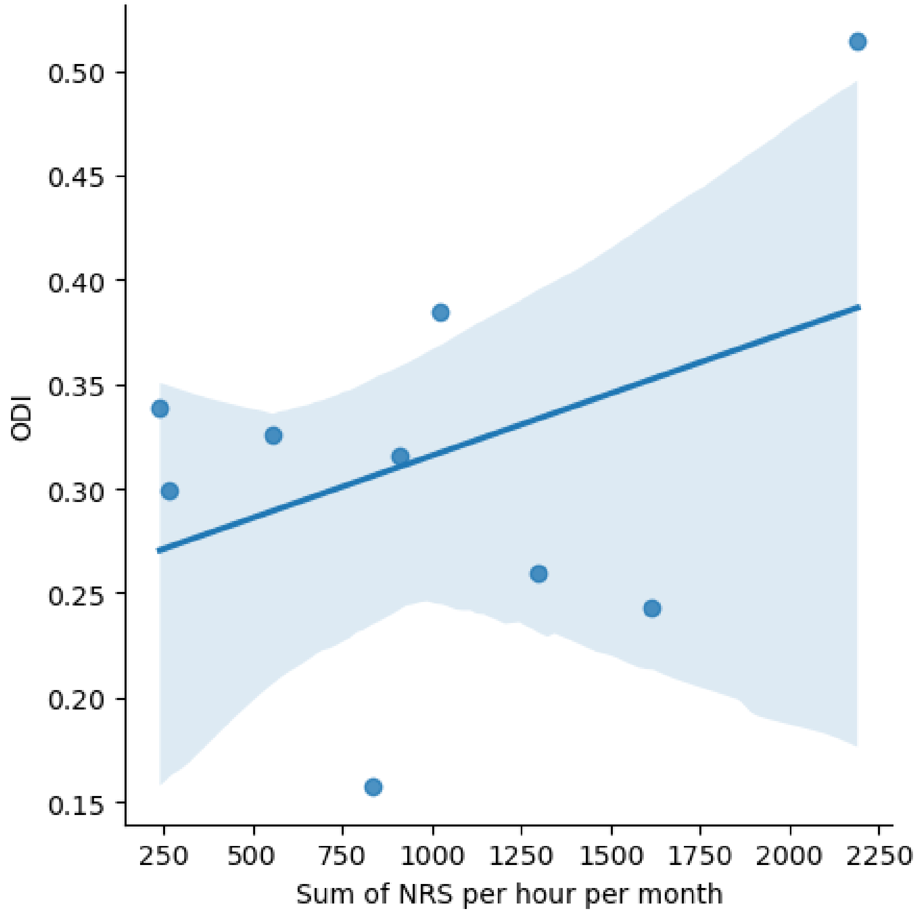

Positive Cluster: 5

Name	Right-Cerebral-White-Matter
CWP	0.04000
Size	111 Voxel
TalX, TalY, TalZ	32.4, 21.82, 27.95
**Linear Regression**	
Slope	0.00007
Intercept	0.16684
R-value	0.61462
P-Value	0.07820
**Spearman Regression**	
Spearman Correlation	0.33333
P-Value	0.38071

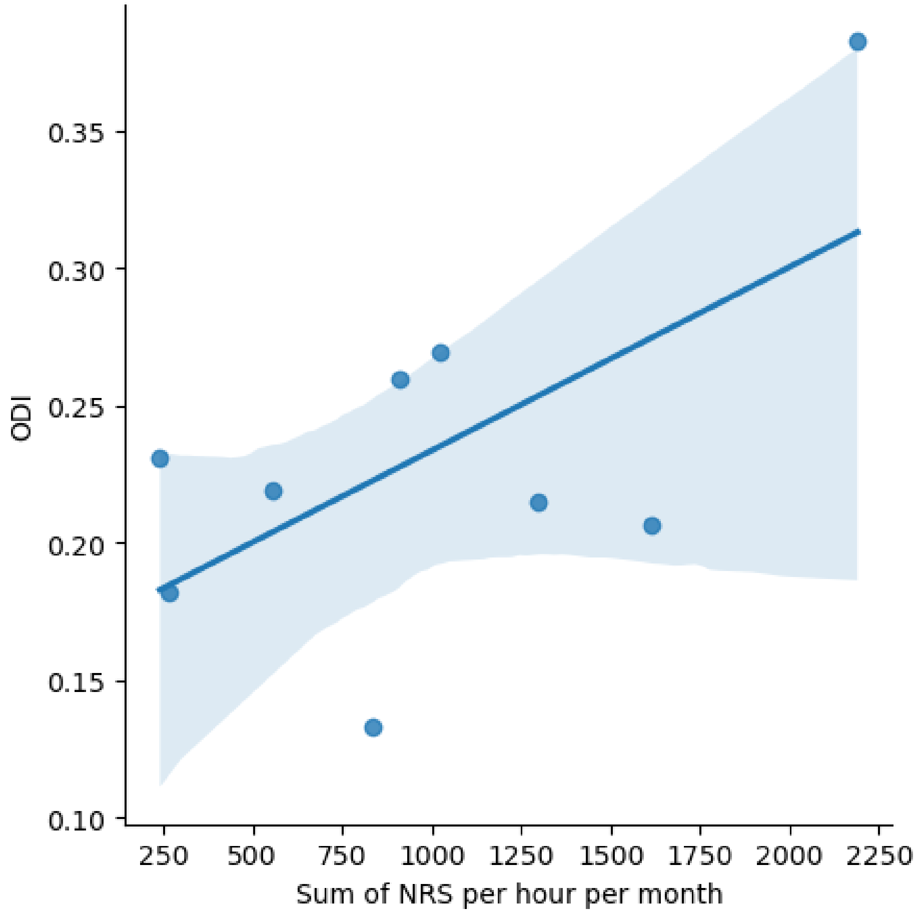

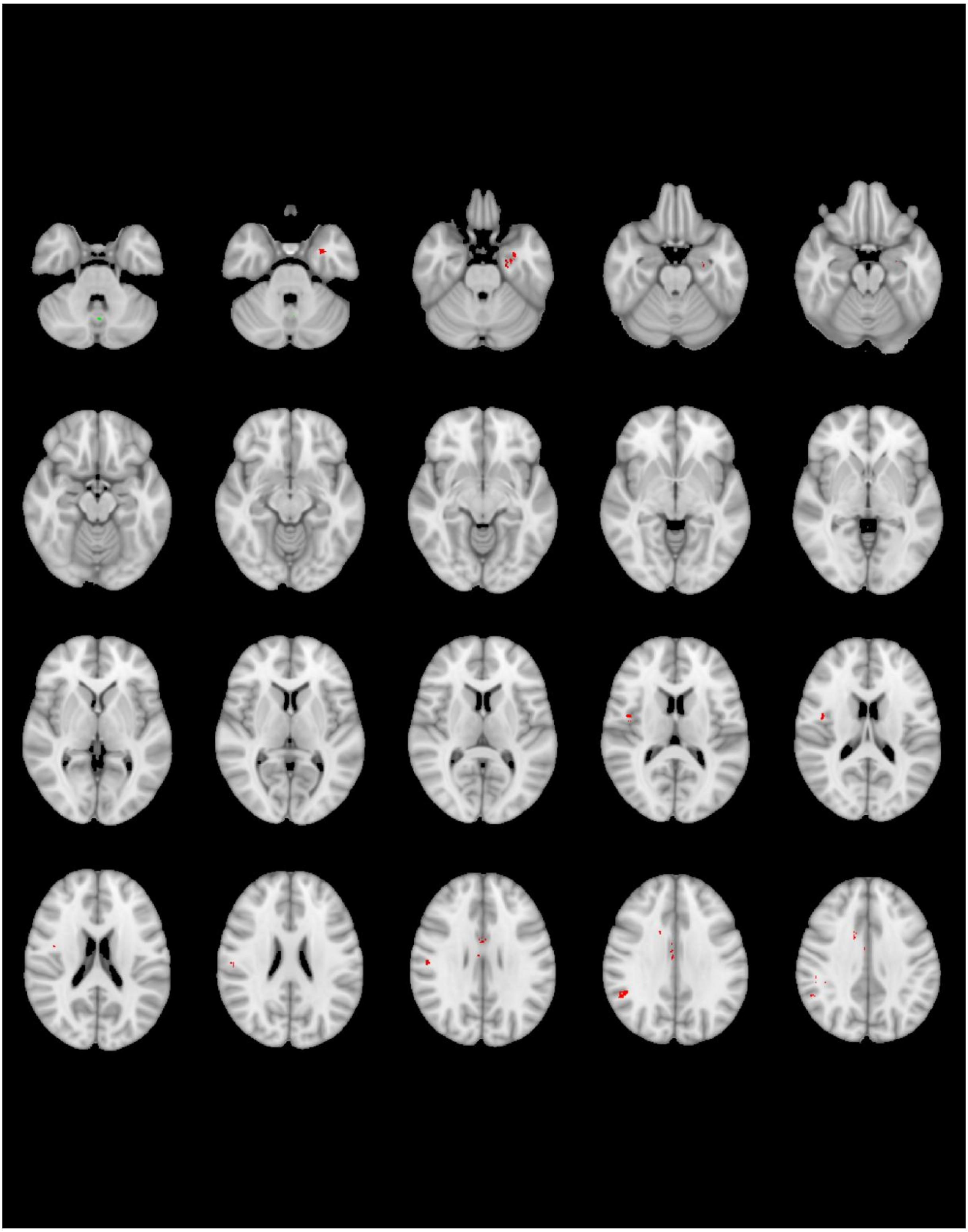

Figure 8 Shown is the negative decadic logarithm of the clusterwise p-values. The threshold for the clusters is a clusterwise p-value < 0.05 (>1.3 for the negative decadic logarithm).
Red marked spots indicate clusters with a negative correlation of the NDI with the sum of NRS per hour per month.
Green marked spots indicate clusters with a negative correlation of the ODI with the sum of NRS per hour per month.

NDI

**Negative Cluster: 1**	
Name	Right-Cerebral-White-Matter
CWP	0.00008
Size	176 Voxel
TalX, TalY, TalZ	50.67, -35.12, 38.83
**Linear Regression**	
Slope	-0.00012
Intercept	0.57435
R-value	-0.62882
P-Value	0.06967
**Spearman Regression**	
Spearman Correlation	-0.51667
P-Value	0.15439

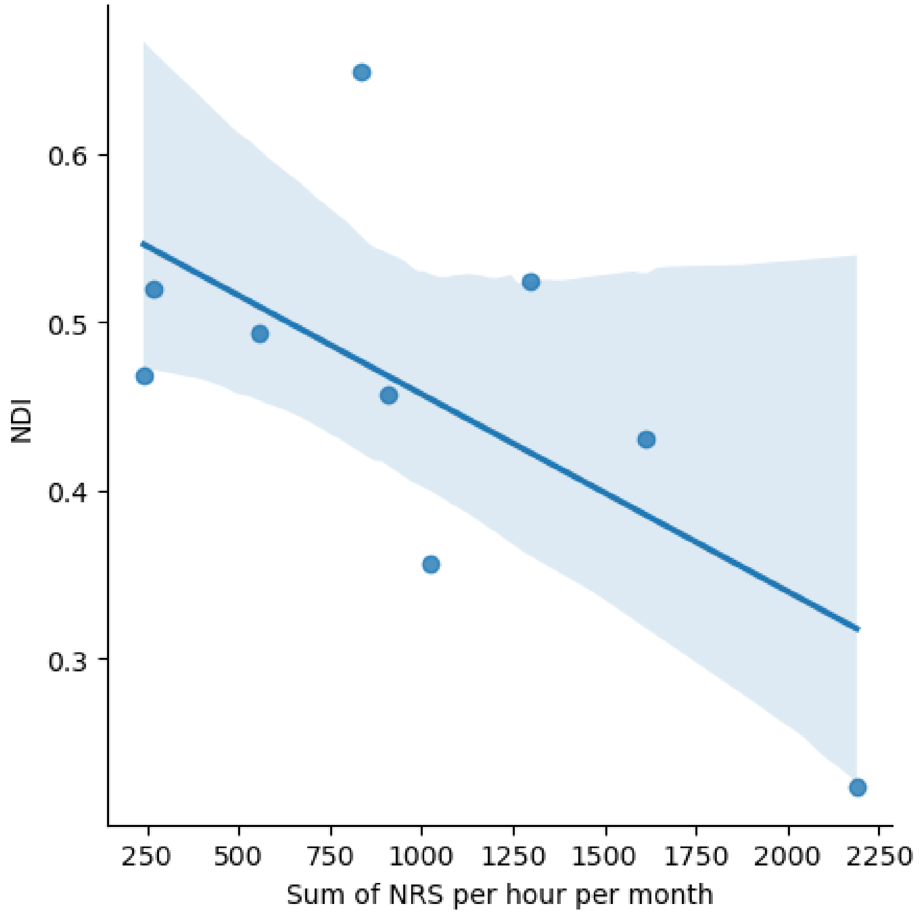

Negative Cluster: 2

Name	Left-Cerebral-White-Matter
CWP	0.00008
Size	176 Voxel
TalX, TalY, TalZ	-29.22, -4.38, -25.59
**Linear Regression**	
Slope	-0.00020
Intercept	0.64842
R-value	-0.83259
P-Value	0.00534
**Spearman Regression**	
Spearman Correlation	-0.73333
P-Value	0.02455

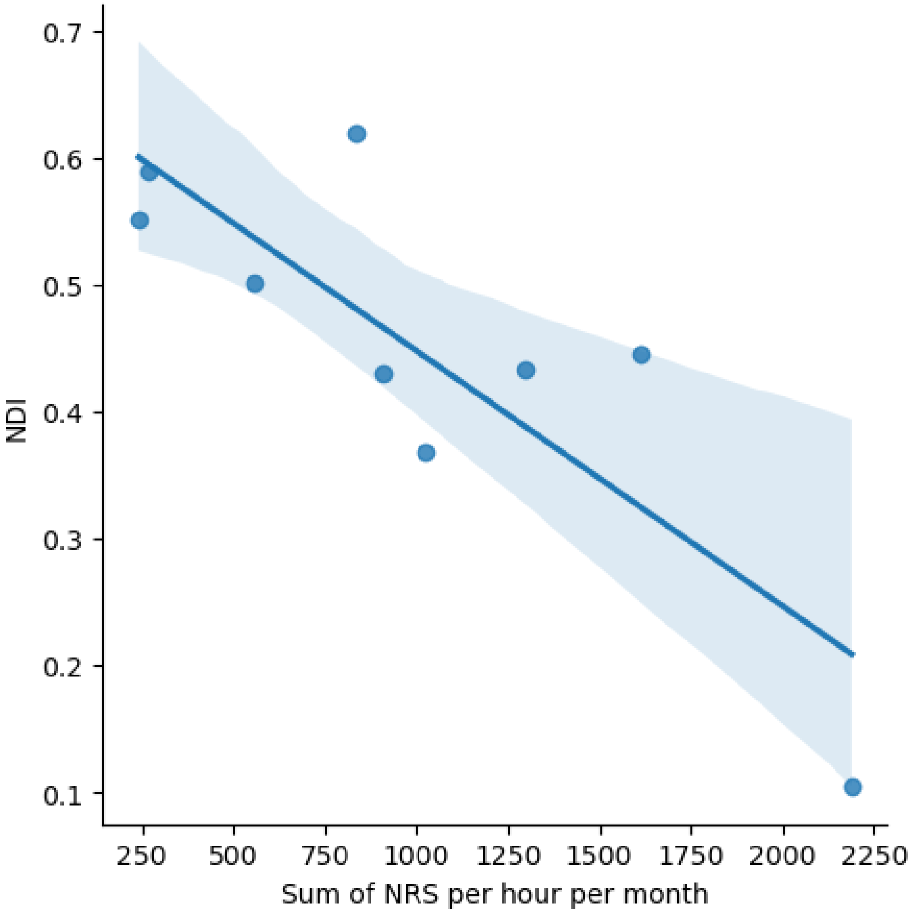

Negative Cluster: 3

Name	Right-Cerebral-White-Matter
CWP	0.00075
Size	149 Voxel
TalX, TalY, TalZ	52.68, -48.08, 32.93

Linear Regression

Slope	-0.00014
Intercept	0.53468
R-value	-0.66926
P-Value	0.04866

Spearman Regression

Spearman Correlation	-0.60000
P-Value	0.08762

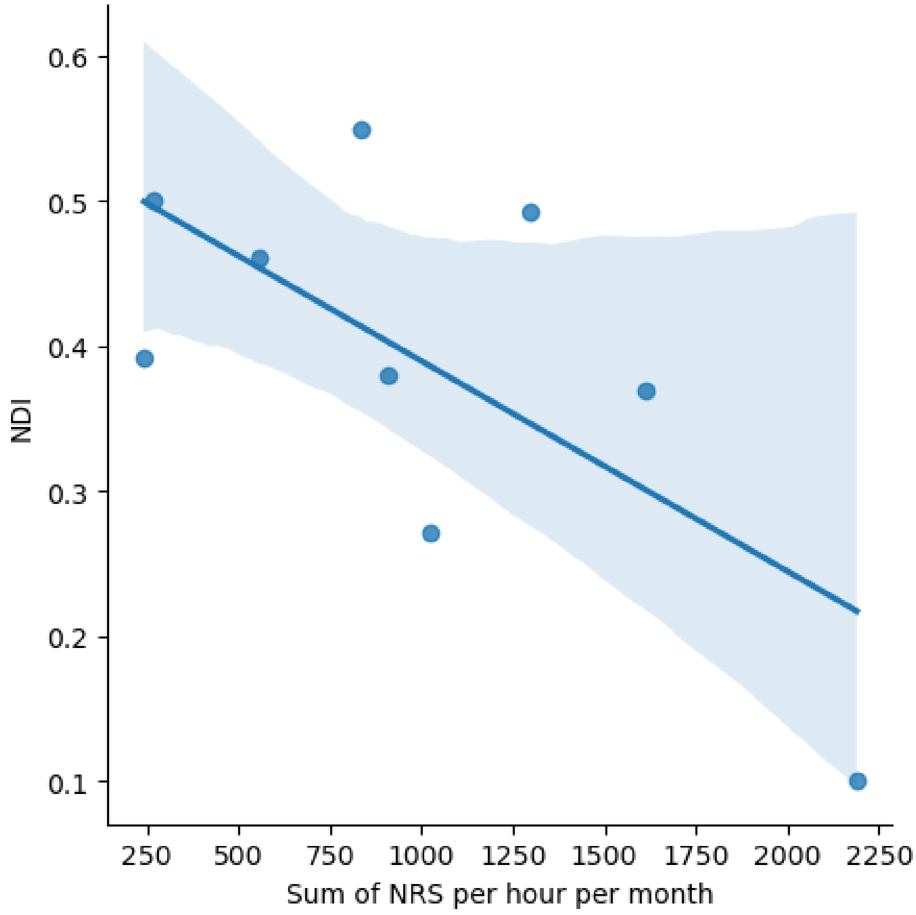

Negative Cluster: 4

Name	ctx-rh-postcentral
CWP	0.00375
Size	124 Voxel
TalX, TalY, TalZ	45.42, -4.4, 12.57
**Linear Regression**	
Slope	-0.00009
Intercept	0.51311
R-value	-0.47179
P-Value	0.19978
**Spearman Regression**	
Spearman Correlation	-0.08333
P-Value	0.83121

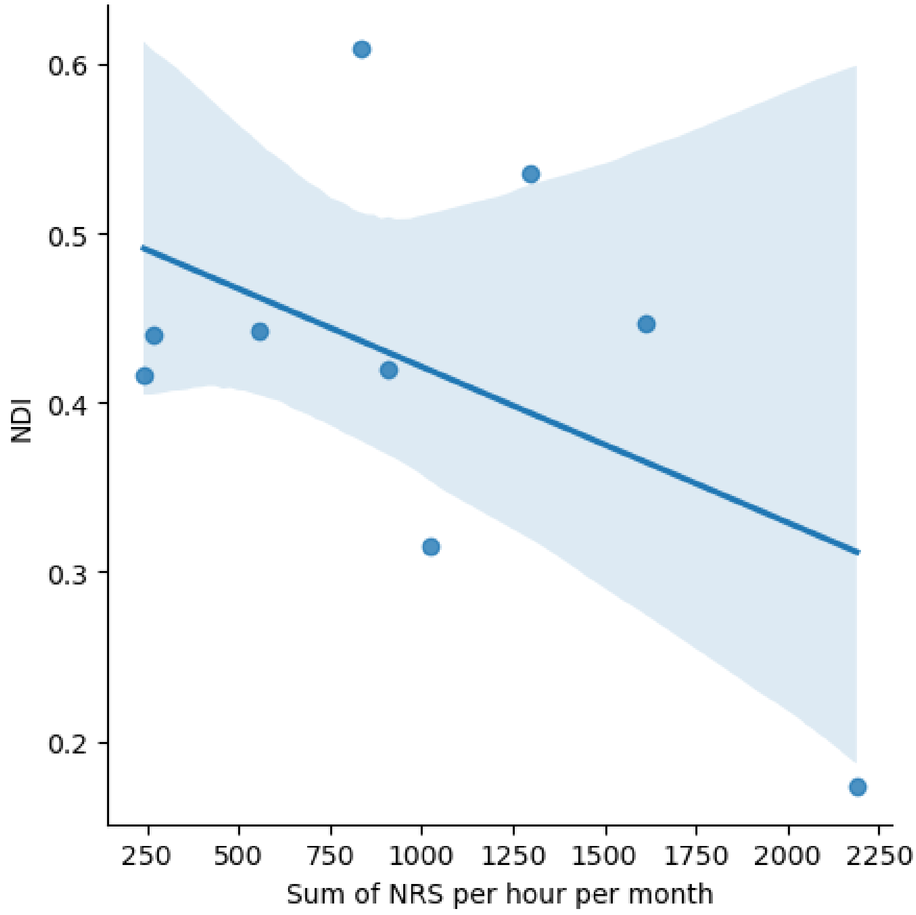

Negative Cluster: 5

Name	Right-Cerebral-White-Matter
CWP	0.00550
Size	120 Voxel
TalX, TalY, TalZ	10.58, -61.1, 50.5
**Linear Regression**	
Slope	-0.00003
Intercept	0.32784
R-value	-0.12474
P-Value	0.74915
**Spearman Regression**	
Spearman Correlation	0.06667
P-Value	0.86469

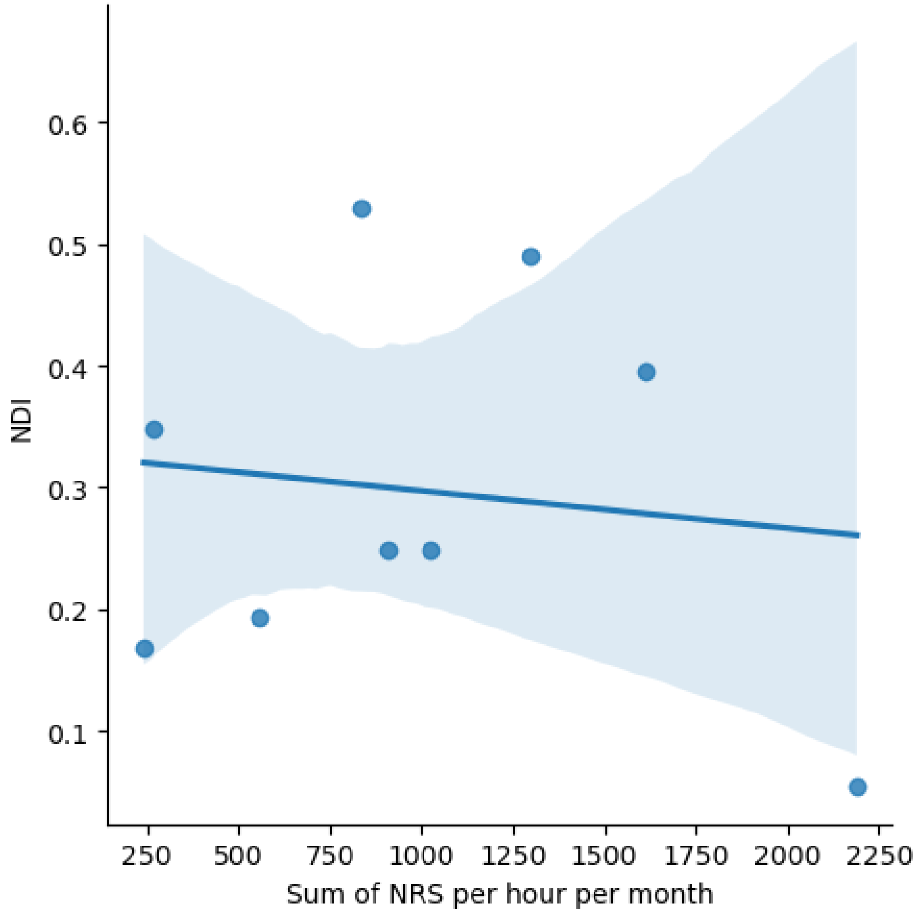

Negative Cluster: 6

Name	Right-Cerebral-White-Matter
CWP	0.00900
Size	114 Voxel
TalX, TalY, TalZ	14.46, 11.42, 28.16
**Linear Regression**	
Slope	-0.00007
Intercept	0.60123
R-value	-0.53795
P-Value	0.13519
**Spearman Regression**	
Spearman Correlation	-0.35000
P-Value	0.35582

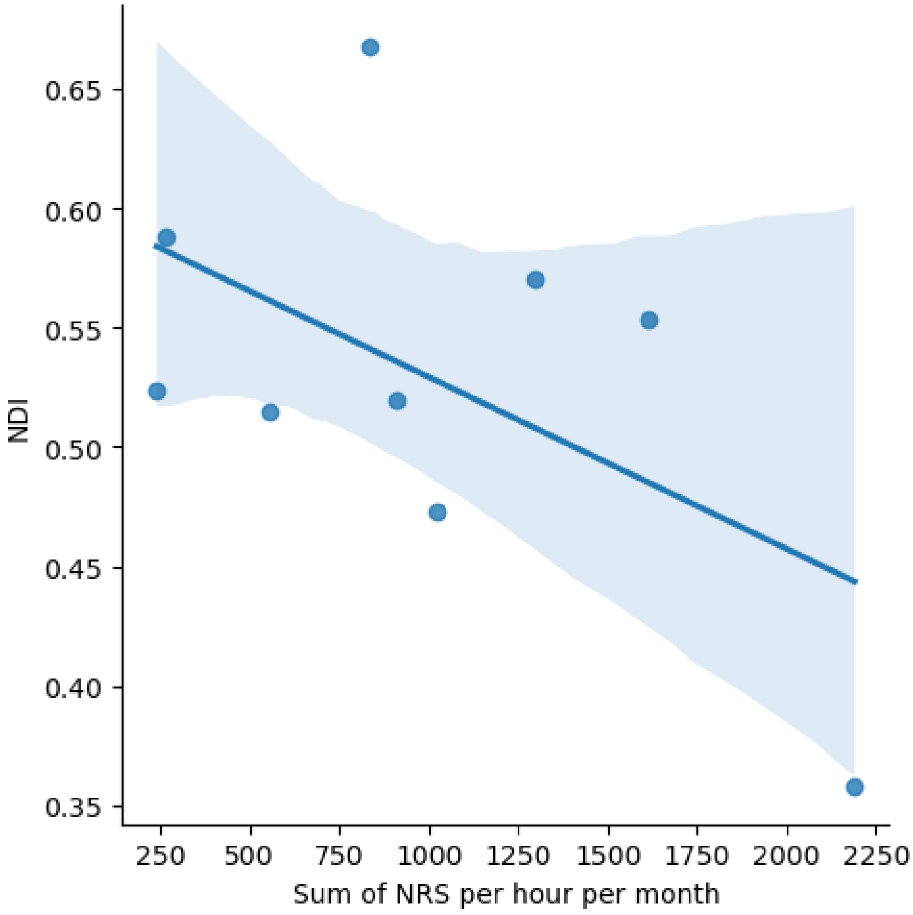

Negative Cluster: 7

Name	Right-Cerebral-White-Matter
CWP	0.01933
Size	106 Voxel
TalX, TalY, TalZ	52.01, -19.31, 26.19
**Linear Regression**	
Slope	-0.00011
Intercept	0.56166
R-value	-0.53050
P-Value	0.14174
**Spearman Regression**	
Spearman Correlation	-0.30000
P-Value	0.43285

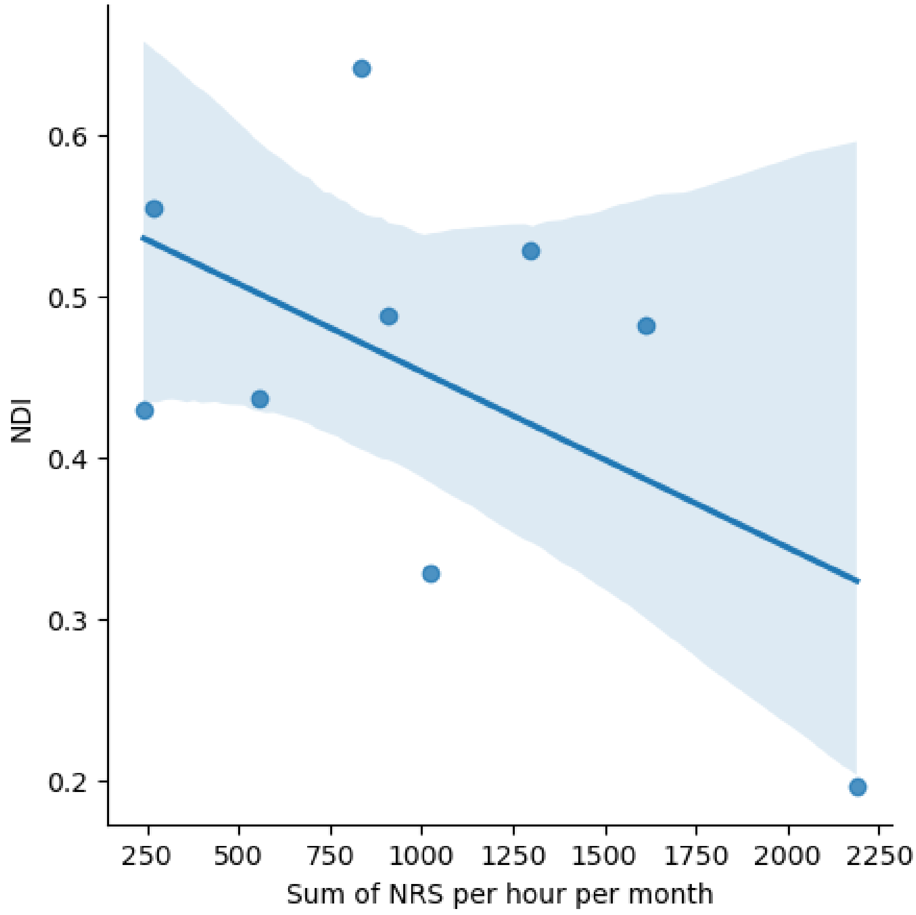

Negative Cluster: 8

Name	Left-Cerebral-White-Matter
CWP	0.01933
Size	106 Voxel
TalX, TalY, TalZ	-2.39, 1.72, 23.74
**Linear Regression**	
Slope	-0.00009
Intercept	0.46616
R-value	-0.57340
P-Value	0.10649
**Spearman Regression**	
Spearman Correlation	-0.20000
P-Value	0.60590

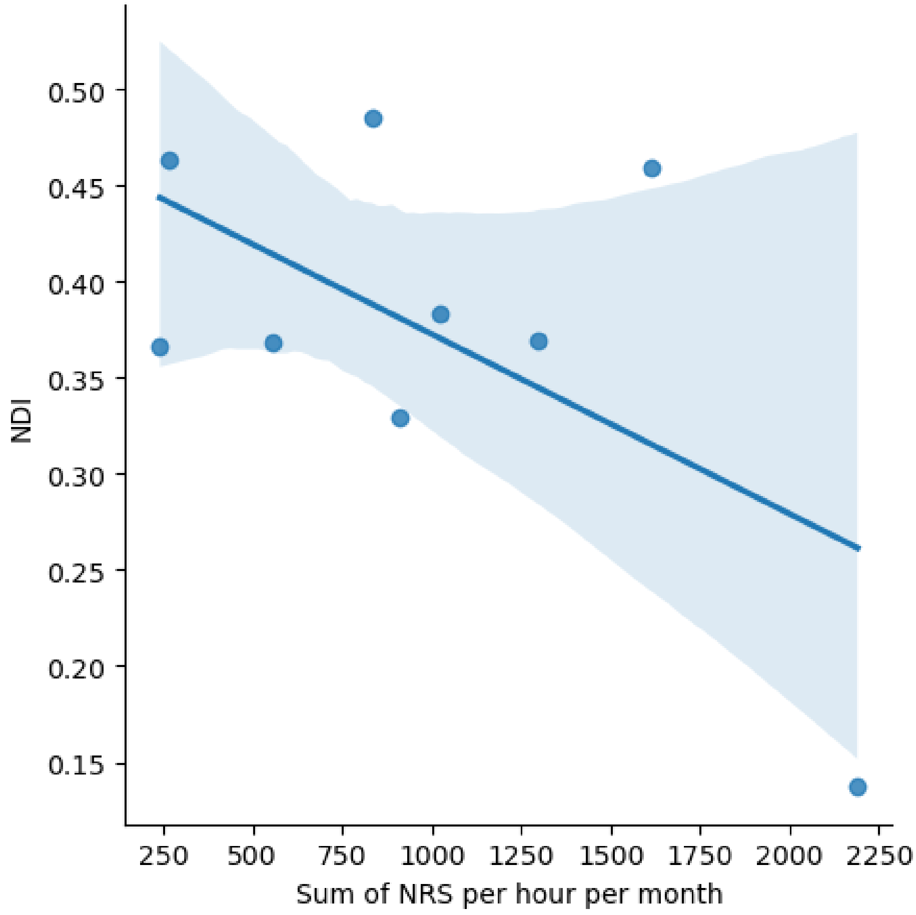

Negative Cluster: 9

Name	Left-Cerebral-White-Matter
CWP	0.03683
Size	99 Voxel
TalX, TalY, TalZ	-21.4, -16.02, -23.33
**Linear Regression**	
Slope	-0.00015
Intercept	0.51644
R-value	-0.77710
P-Value	0.01374
**Spearman Regression**	
Spearman Correlation	-0.56667
P-Value	0.11163

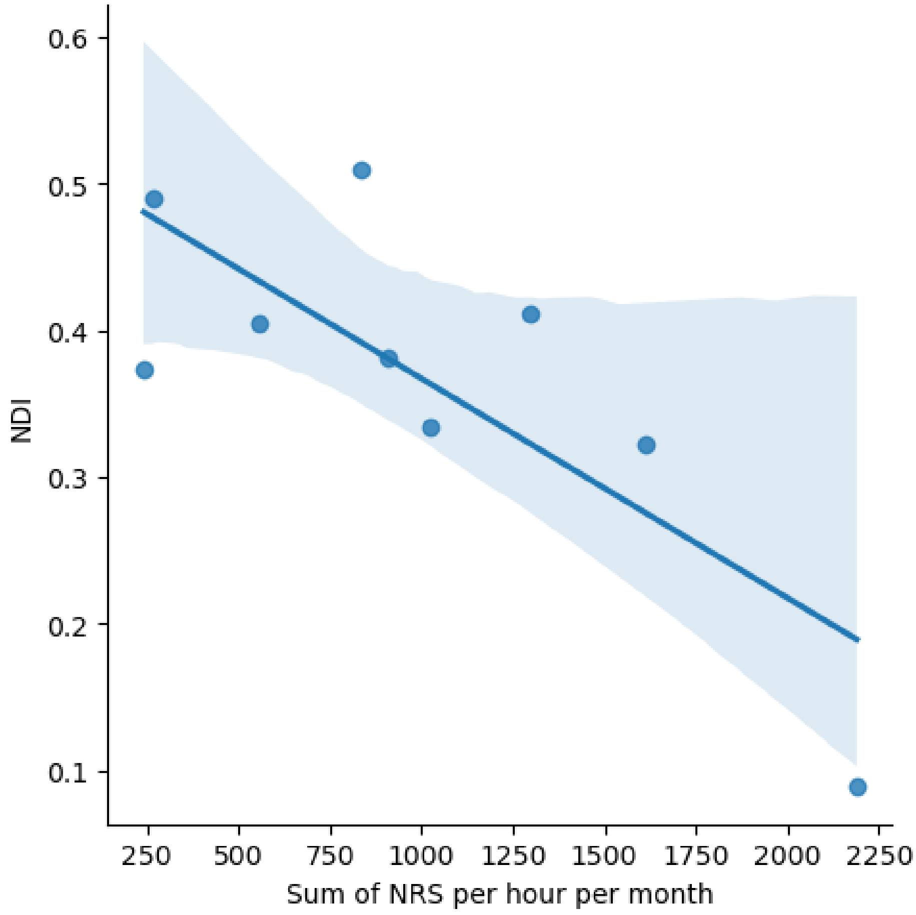

ODI

**Negative Cluster: 1**	
Name	Left-Cerebellum-Cortex
CWP	0.01250
Size	128 Voxel
TalX, TalY, TalZ	0.22, -66.1, -28.58
**Linear Regression**	
Slope	-0.00012
Intercept	0.46988
R-value	-0.79093
P-Value	0.01114
**Spearman Regression**	
Spearman Correlation	-0.66667
P-Value	0.04987

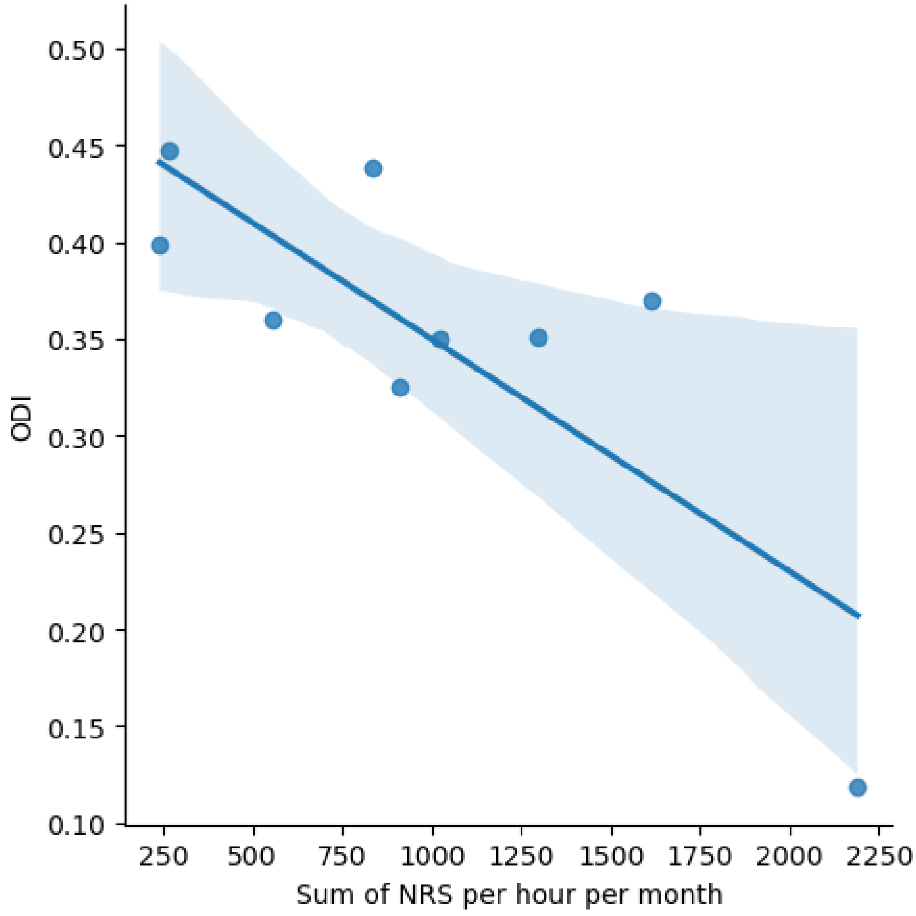

Supplement: Supplementary file 1 [file Datasheet1.pdf]
